# Spatial memory distortions for the shapes of walked paths occur in violation of physically experienced geometry

**DOI:** 10.1371/journal.pone.0281739

**Published:** 2023-02-10

**Authors:** Yu K. Du, Andrew S. McAvan, Jingyi Zheng, Arne D. Ekstrom

**Affiliations:** 1 Department of Psychology, University of Arizona, Tucson, AZ, United States of America; 2 Department of Mathematics and Statistics, Auburn University, Auburn, AL, United States of America; 3 Evelyn McKnight Brain Institute, University of Arizona, Tucson, AZ, United States of America; Kennedy Krieger Institute/Johns Hopkins University School of Medicine, UNITED STATES

## Abstract

An important question regards the nature of our spatial memories for the paths that we have walked and, in particular, whether such distortions might violate the topological properties of the shape of the paths (i.e., creating an intersection when two paths did not intersect or vice versa). To investigate whether and how this might occur, we tested humans in situations in which they walked simple paths and idiothetic and visual cues either matched or mismatched, with the mismatching cues creating the greatest potential for topological distortions. Participants walked four-segment paths with 90° turns in immersive virtual reality and pointed to their start location when they arrived at the end of the path. In paths with a crossing, when the intersection was not presented, participants pointed to a novel start location suggesting a topological distortion involving non-crossed paths. In paths without a crossing, when a false intersection was presented, participants pointed to a novel start location suggesting a topological distortion involving crossed paths. In paths without crossings and without false intersections, participants showed reduced pointing errors that typically did not involve topological distortions. Distortions more generally, as indicated by pointing errors to the start location, were significantly reduced for walked paths involving primarily idiothetic cues with limited visual cues; conversely, distortions were significantly increased when idiothetic cues were diminished and navigation relied primarily on visual cues. Our findings suggest that our spatial memories for walked paths sometimes involve topological distortions, particularly when resolving the competition between idiothetic and visual cues.

## 1. Introduction

Numerous studies suggest that our spatial memories for places that we have navigated are distorted versions of what we experience in the real-world [1–9]. While such distortions are likely more pronounced for environmental configurations or paths that are new to us, previous work suggests that even configurations and paths with which we are familiar involve distortions from their true physical configuration and shape [3,4,9]. For example, past work has suggested that we may retrieve locations and objects at different positions than their true ones based on 1) how we imagine the surrounding geometry [5], 2) experiencing unreliable landmarks [10], 3) accumulated error in our estimates about position from self-motion cues [1,11], and 4) cue competition [12–14]. Exactly what factors effect spatial memory distortions remain unclear yet are important to better understand how we represent space as we navigate and learn it.

Previous studies of human spatial navigation suggest instances in which such distortions for remembered locations or routes may violate one or more geometric principles. For example, several studies report distortions in spatial memory that involve geometrically impossible spatial reconstructions when participants remember familiar spatial layouts, for example, violating the property of triangle inequality [3,7] or symmetry [2]. Other studies have looked at navigating impossible virtual environments that loop back on themselves in ways that violate physical reality [15–18] or navigating an environment containing wormholes [19–21]. For example, when participants learned regular virtual environments and then experienced wormholes that teleported them to a distinct location, the wormholes induced significant non-Euclidean distortions despite the fact that the majority of participants were unaware of the wormholes’ presence [21]. The fact that such violations of physical space–the wormholes involved translations and rotations that violate the axioms described above–were readily accommodated by participants’ routes and pointing patterns, supported the idea that such spatial representations might better resemble “labeled graphs.” According to labeled graph theory, paths are tagged with local distances but not globally fixed in terms of their metric relationships, allowing for violations of Euclidean axioms [22,23].

Although past research has shown that humans may form distorted memories that violate Euclidean geometry in some situations, it has not been thoroughly examined whether our spatial memories consistently maintain the same *topological* relationships. As an important underlying aspect of spatial relationships in our real life, topological relationships are essential to successfully navigating our daily environments. For example, in a grid-like city, some streets intersect with each other (i.e., at 90°) while others are parallel to each other. Intersecting vs. parallel are different topologically (i.e., the “connection” differs based on the presence of an intersection; a node vs. the lack of one). On one hand, it seems unquestionable that the topological relationships of the physical environment should be robust in our spatial memories regardless of Euclidean geometric manipulations (e.g., shortening a path) because topology tends to be fixed in everyday life. For example, it would be very unusual for two parallel streets to gain an intersection (unless significant construction occurred), with such knowledge about topological spatial relationships likely acquired early in life [24].

On the other hand, such topological knowledge may nonetheless be flexible in some instances. Previous studies [16,19–21] showed that people might extend or shorten certain parts of a path in memory to accommodate the existence of wormholes in the environment, sometimes violating topological principles (e.g., extending a path to connect with another disjoint path would change the connectivity). Interestingly, people often may not realize such violations. For example, they might draw a sketch map after the experiment showing that a path was connected to another path from the beginning, although this was not true [21]. These observations suggest that spatial memory distortions may violate some topological relationships of the environment in some instances, although when and how this happens remains largely untested.

**Fig 1:**
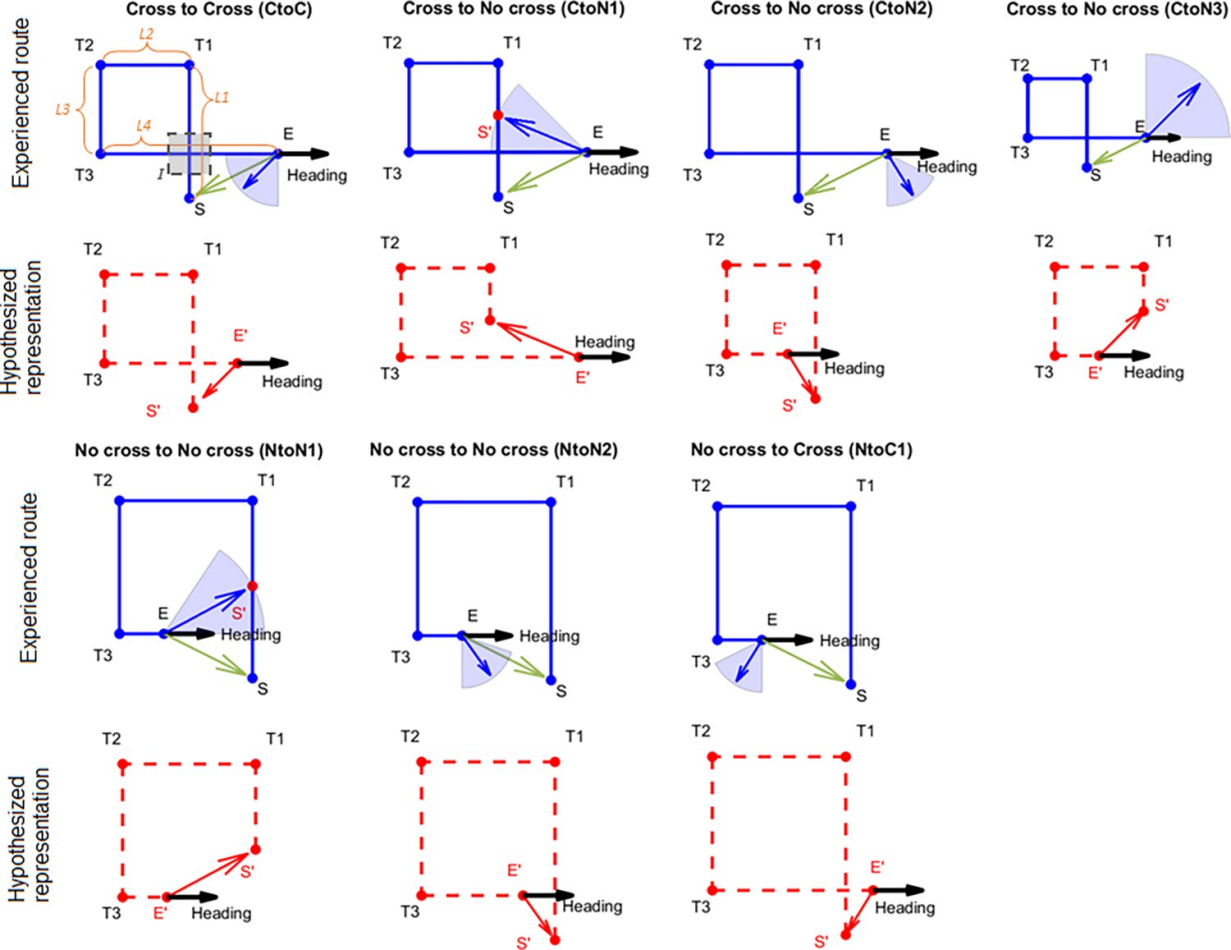
Predicted pointing directions according to each hypothesis in Experiment 1. The example crossed path is Path 3 and the example uncrossed path is Path 7. Blue lines indicate the actual path. Red dashed lines indicate the hypothesized represented path. Green arrows indicate the geometric correct pointing direction at the end of the path (E). Blue shaded areas indicate the actual predicted pointing direction ranges based on the red hypothesized representations immediately below. Blue arrows indicate the mean of the actual predicted ranges for the red hypothesized representations below. Red arrows indicate predicted pointing direction at the represented end (E’). S’: Estimated start position. Top left panel: The grey shaded area shows the hidden intersection area. L1-4: Leg 1–4 according to Euclidean geometry. I: Intersection.

Of note, the studies mentioned above used complex mazes including many routes, turns, and locations to be memorized. Extending a path would likely connect it to another path and thus change the connectivity between them. It is possible that participants extended the path they were walking in their representation (violating Euclidean axioms) without noticing the unavoidable violation of topological principles. In a less complex environment, when remembering one’s start location necessitates resolving conflicts between visual and idiothetic cues, it is unclear whether topological violations would still manifest. Therefore, the current study aims to examine whether visual-idiothetic conflicts in a simple route can lead to memory distortions with topological violations. In particular, we examined changes in path connectivity. For example, a path with four segments and three 90° turns can either be a crossed path or a non-crossed path, depending on the lengths of the segments. The connectivity in a crossed path is different from that in an uncrossed path: the first and the fourth segments in the former case intersect with each other whereas they do not intersect or connect in the latter case. Spatial memory distortions should not alter the postulate that the distance between two points should remain positive. For example, such distortions could involve lengthening or shortening the shape of a path (e.g., Fig 1, Cross to Cross). They should not, however, involve creating an uncrossed shape from a crossed one (e.g., Fig 1, Cross to No cross). This is because the intersecting node in the physically crossed path is a single point of zero distance but in the uncrossed path is essentially “deleted,” creating a positive distance and violating metric postulates about physical space.

In addition, it is notable that several of the studies that have shown violations of Euclidean geometry have involved desktop virtual reality [18]. In these cases, one possibility is that participants’ spatial memories may be influenced primarily by what they experience visually. Because desktop virtual reality involves impoverished idiothetic cues (body-based movements are absent), it is possible that participants are simply relying on visual cues to a greater extent than idiothetic cues, leading to the violations [25]. In immersive VR, when idiothetic and visual input mismatch sufficiently, one possibility is that participants may ignore idiothetic cues and focus solely on visual cues, particularly because path integration computations accumulate error as a function of walked distance [1,26–28]. Therefore, it is important to test spatial distortions for the shapes of paths under a range of different situations. These include situations in which visual and idiothetic cues mismatch, situations in which they match, situations in which primarily idiothetic cues dominate (i.e., with limited visual input), and situations in which primarily visual cues dominate (i.e., desktop VR).

In the current study, we had participants navigate four-sided shapes that either involved crossed or uncrossed paths. Such hallway shapes would be familiar from navigating in everyday buildings. Participants walked the paths in visually rendered hallways in which the expected intersections were either correctly rendered or mismatched with their walked path. To determine the shape that participants thought that they walked, we had them point back to their start position, either by simply determining the direction of the start point relative to the endpoint (Experiments 1 and 4) or by indicating both direction and distance (Experiments 2 and 3). The direction and location that they selected as where they thought they started were used as our primary dependent measure. By having participants point to their remembered starting location, we could determine whether the remembered start point involved either a crossed or uncrossed path based on what they had just navigated. Having them point to their remembered start location was advantageous compared to map drawing or having them retrace their paths as this would potentially bias how they might encode future paths on later trials. In addition, several studies suggest that pointing tasks provide insight into participants’ spatial knowledge comparable to what they might draw with a map or walk as a route [29,30] and are sensitive to memory distortions related to spatial geometry [5,6,15,31,32].

**Fig 2:**
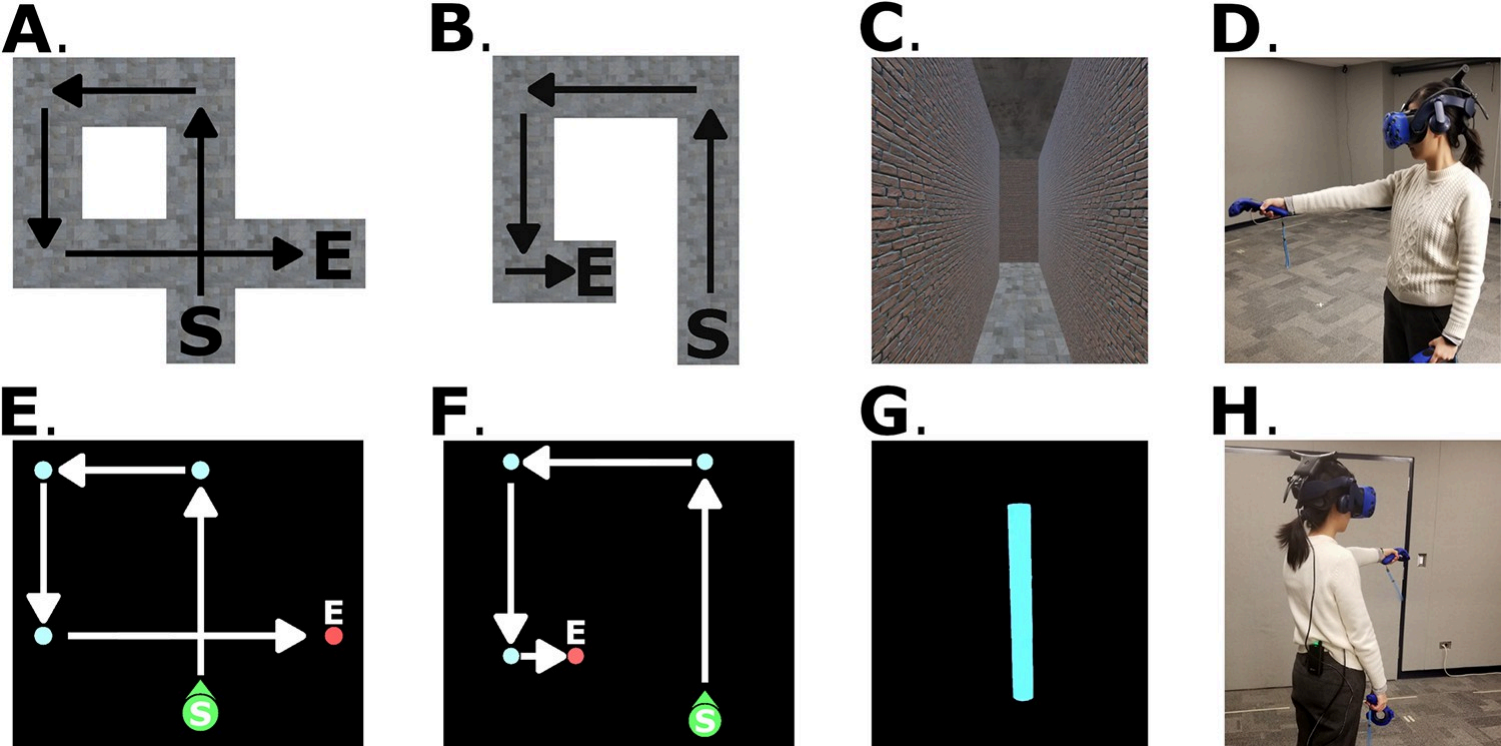
Example paths in Experiment 1 for the (A) hidden intersection cross (HI-C), (B) no intersection no cross (NI-NC), (E) pole-guided cross (PG-C), and (F) pole-guided no cross (PG-NC) conditions. C & G show the participant’s perspective from within the virtual environment (brightness adjusted for illustration purposes). D & H show apparatus and the testing room. S: Start. E: End. Arrows indicate the walking direction.

In Experiment 1a, we compared a situation in which participants (the hallway navigation group) crossed their path on the fourth leg of a hallway but did not see the expected intersection (i.e., the removal of the intersection was not something they were informed of, the “hidden intersection cross” condition, HI-C; Table 1, Fig 2A) with a situation in which the path itself never crossed itself (the “no intersection—no cross” condition, NI-NC; again, participants were not informed about the nature of the condition; Fig 2B). In the former case, if participants follow their idiothetic (path integration) cues, they should ignore the missing visual information and point to their start location based on the paths they walked. Alternatively, they might follow the mismatching visual cues and point to a start location that involves creating an uncrossed path (or some intermediate). In Experiment 1b, we tested whether distortions might occur based on primarily on idiothetic cues by presenting poles in darkness to guide navigation instead of hallways (the pole-guided group; Fig 2E and 2F). In Experiment 1c, the same conditions as Experiment 1a were used in a blocked-design order instead of a random-mixed order for the purpose of replication.

**Table 1:** Experimental design and conditions.

Intersection visibility	Intersection hidden or not existing		True/false intersection shown	
Path type	Cross	No cross	Cross	No cross
**Experiment 1a & 1c** Hallway mixed- and blocked-design groups	**Hidden intersection cross (HI-C)**	**No intersection no cross (NI-NC)**	N/A	N/A
	Participants walked a hallway; walked a crossed path; true intersection hidden (not shown)	Participants walked a hallway; the walked path did not cross (non-intersecting); no false intersections shown		
**Experiment 1b** Pole-guided group	**Pole-guided cross (PG-C)**	**Pole-guided no cross (PG-NC)**	N/A	N/A
	Participants were guided by four poles at three turning points and the end; walked a crossed path; true intersection hidden (not shown)	Participants were guided by four poles at three turning points and the end; the walked path did not cross (non-intersecting); no false intersections shown		
**Experiment 2 (mixed-design)**	**Hidden intersection cross (HI-C)**	**No intersection no cross (NI-NC)**	**True intersection cross (TI-C)**	**False intersection no cross (FI-NC)**
	Participants walked a hallway; walked a crossed path; true intersection hidden (not shown)	Participants walked a hallway; the walked path did not cross (non-intersecting); no false intersections shown	Participants walked a hallway; walked a crossed path; true intersection shown	Participants walked a hallway; the walked path did not cross; false intersections shown
**Experiment 3 (blocked-design)**	N/A	N/A	**True intersection cross (TI-C)**	**False intersection no cross (FI-NC)**
			Participants walked a hallway; walked a crossed path; true intersection shown	Participants walked a hallway; the walked path did not cross; false intersections shown
**Experiment 4 (desktop VR; blocked-design)**	**Hidden intersection cross (HI-C)**	**No intersection no cross (NI-NC)**	**True intersection cross (TI-C)**	**False intersection no cross (FI-NC)**
	Participants navigated a hallway with a joystick; navigated a crossed path; true intersection hidden (not shown)	Participants navigated a hallway with a joystick; the path did not cross (non-intersecting); no false intersections shown	Participants navigated a hallway with a joystick; navigated a crossed path; true intersection shown	Participants navigated a hallway with a joystick; the walked path did not cross; false intersections shown

In Experiment 2, we focused on whether participants would ignore the mismatching visual intersections and point based on their idiothetic cues when encountering an intersection that could not possibly occur in uncrossed hallways (the “false intersection no cross” condition, FI-NC). Alternatively, they might point in a way that involves a false intersection from memory based on the mismatching visual cues (or some combination of the two). In Experiment 3, we used a blocked design to replicate the results of the FI-NC condition and one of the control conditions involving a “true” intersection (TI-C) in Experiment 2. We compared the angular pointing responses with hypothesized pointing intervals to try to understand the nature of the possible memory distortions to determine whether and how participants might reconstruct their memories for the paths they walked. We did this to try to better determine whether the randomization of different conditions in Experiments 1&2, might be influencing each other, in particular, whether the true intersection—cross condition (TI-C) might have been influenced by the hidden intersection–cross condition (HI-C) and vice versa as they involved the same crossed paths. In Experiment 4, we employed an identical design to Experiment 2 but using desktop virtual reality in which body-based cues were absent, degrading the fidelity of idiothetic cues and selectively emphasizing visual cues. This was included to determine whether any topological distortions might arise from a simple preference for visual input or whether the distortions in Experiments 1–3 came about instead from competition and integration of idiothetic and visual input [12–14,33,34].

## 2. Experiment 1

Experiment 1 included three sub-experiments which were tested sequentially. Therefore, we present the methods and the results under the name of Experiment 1a, 1b and 1c for each group, respectively (Table 1).

### 2.1. Methods

#### 2.1.1. Participants

We recruited 84 participants (53 female, 31 male) from the University of Arizona undergraduate Psychology program and surrounding Tucson city area whose ages ranged from 18 to 46 years old with a mean age of 20.21. Participants were assigned into three separate groups: the hallway navigation mixed-design group (n = 32), the pole-guided navigation group (n = 21), and the hallway navigation blocked-design group (n = 31). The three groups were tested independently of one another with the hallway navigation mixed-design group tested first, followed by the other two groups. All participants received either course credit for their participation or were compensated at a rate of $20 per hour. All participants had normal or corrected-to-normal color vision, normal or corrected-to-normal hearing, and reported no history of cardio-vascular problems or motion sickness. Written consent was obtained before the experiment, and the methods were approved by the Institutional Review Board (IRB) at the University of Arizona. We based our sample size (21, allowing for some attrition) on a previous study by Warren et al. [21] that also involved participants navigating with an HMD in spaces that involved violations of Euclidean axioms. Because of the novelty of our design and hypotheses (outlined above and in the methods), our exact effect sizes were unknown.

#### 2.1.2. Apparatus

The experiment was conducted in a room approximately 6 m × 6 m in size with the navigable virtual environment being approximately 5 m × 5 m in size. The virtual environment and experimental tasks were built in Unity 3D (Unity Technologies ApS, San Francisco, CA) using the Landmarks virtual reality navigation package [35]. Participants experienced the fully immersive virtual environments via the use of an HTC Vive Pro head-mounted display (HMD) fitted with an HTC Wireless Adapter (HTC, New Taipei City, Taiwan) that allowed for untethered, free ambulation (Fig 2D and 2H). The Vive Pro displayed stimuli at a resolution of 1140 × 1600 pixels per eye with a 110° field of view (FOV) refreshed at a rate of 90 Hz. The Wireless Adapter allowed for participants’ orientation and position to be tracked for up to 7 m, with data being delivered over a 60 GHz radio frequency. To record responses from participants and allow for interaction with the virtual environment, we used two handheld HTC Vive controllers (HTC, New Taipei City, Taiwan). The tasks were run on a custom-built computer with an NVIDIA GeForce Titan Xp graphics card (NVIDIA Corp., Santa Clara, CA).

#### 2.1.3. Design

All three groups received a total of 32 trials with the same eight path shapes in two conditions (Cross or No cross; Table 1). In total, each participant experienced 16 cross and 16 no cross trials. There were four different path shapes for each condition (Fig 2A and 2B), S1–S3 Figs, S1 Table). Each path had four segments (“legs”) with three clearly visible 90° turns (left/right) requiring moving the body 90°. Each path shape was presented for four trials in total.

For the two hallway navigation groups (Experiment 1a and 1c), both received the same stimuli which were eight different hallway models (i.e., 4 path shapes × 2 left/right turning directions) in the experimental program for each condition. Each of these eight hallway models was presented twice with different orientations in the virtual environment. For each participant, the two presentation orientations of each hallway model were randomly chosen from four orientations with respect to the physical room (i.e., 0°, 90°, 180°, or 270° rotated clockwise from the model’s original orientation). For these hallway models, different textures and colors were applied to the walls to ensure that each hall was distinct and provided a sense of optic flow to participants (Fig 2C). For the cross condition, hallways with an intersection (i.e., crossing between the first and the last segments of the path) were presented (termed “hidden intersection cross”, HI-C; Table 1, Fig 2A). For the no cross condition, hallways without such intersections were presented (termed “no intersection no cross”, NI-NC; Fig 2B).

The only difference between the two hallway navigation groups was the trial presentation order. The mixed-design group (Experiment 1a) received all trials in a mixed, randomly determined order whereas the blocked-design group (Experiment 1c) received the two conditions in a sequential order. For Experiment 1c, the condition order (i.e., which condition was first tested) was counterbalanced across participants. Within each block, the trials representing a specific condition were presented in a random order such that the paths were completely randomized. For all groups, trials with the same path shape were never presented immediately one after another.

Participants in the pole-guided navigation group (Experiment 1b) received the same paths (in random orders) except that no visual (optic flow) information was presented to them. Instead, virtual slim cylinders (“poles”) were presented in sequence at each of the three turns and the end of the hall (Fig 2E, 2F and 2G). The poles were 2.13 m in height and 0.21 m in diameter. As a control group, there were no visual walls, texture, halls, or floor presented but only four poles to guide walking in the darkness. We termed the conditions for this group as “pole-guided cross” (PG-C) and “pole-guided no cross” (PG-NC; Table 1) because there was no “intersection” with walls to be shown or be hidden. We conducted the hallway vs. pole-guided manipulation as between-subject to ensure that seeing or walking a path and solely experiencing idiothetic cues related to walking the path did not influence whether visual or body-motion cues were differentially emphasized.

To test our hypothesis regarding how visual expectations would affect participant memories for paths they walked in Experiments 1a & c, we did not render the final intersection in the hidden intersection—cross (HI-C) condition for the hallway navigation groups. Specifically, when participants walked through these “hidden intersection” hallways, the last path was not shown to be intersecting the first path and vice versa (Fig 2A). Because only poles were shown in the pole-guided group (Experiment 1b), which appeared to show participants where to walk, there would be no visual expectation of crossing a path.

Two additional path shapes were used in the practice prior to the experimental trials to familiarize the participants with the paradigm. Both had two paths of equal distance (2.55 meters), with one containing a single 90° left turn and the other containing a single 90° right turn. For the pole-guided group, the practice paths were presented by showing the poles. For the other two groups, the paths were presented by showing hallways (which used a wall texture different from those in the formal trials).

#### 2.1.4. Procedure

All three groups received the same procedure. After reading and signing the consent form, participants were blindfolded and led into the testing room. Then, they were fitted with the wireless HMD, the handheld controller(s), and a clip-on battery pack that powered the wireless HMD (Fig 2D and 2H). Once immersed in VR, the participants were led through four practice trials. The practice and experimental trials followed the same procedures (see example videos at https://osf.io/3aygm/).

On each trial, participants were instructed to rotate slowly in place while verbally calculating subtractions for approximately 10 seconds (e.g., “Please rotate leftward in place and verbally count backward from 100 by 3”). This procedure was intended to disorient participants from any previously gained spatial knowledge. The participants were then presented with a darkened environment and were asked to search for and walk to a red platform with a directional arrow. When they arrived at the platform, it turned green, and then they were asked to face the same direction as the arrow. The platform indicated the start of the path, and the participants were asked to remember this position while walking. When participants pressed a button on the controller, the platform disappeared and the hallway or the pole appeared.

After that, for the hallway navigation groups, the participants stood at the start of the hallway and walked the length of the four hall segments until they arrived at the end (indicated by an ending wall). Once at the end, they then used the controller to point in the direction of the start of the hallway. The participants were instructed to point back to their starting location as accurately as possible, and that response time would not be recorded; only the response direction angle was recorded (in a range of 0° to 360°) in Experiment 1. The participants were allowed to turn their bodies and see the last segment of the hall while pointing but were not allowed to move away from the end location. The walls blocked them from seeing the other segments. After making their pointing response, the hallway disappeared, and a new trial began.

For the pole-guided group, when the participants stood at the starting platform and presses the button on the controller, a blue pole was shown in front of them indicating the first turning point of the path. Once they walked to the first pole, it disappeared, and the next sequential pole appeared. One of the controllers in their hands would vibrate to indicate which direction to turn until the participants were facing the next pole. They walked to each pole until they arrived at a red pole which indicated the end of the path. Participants then performed the same pointing task as the hallway group. None of the poles was visible during the pointing. This procedure excluded extra visual piloting cues, minimized optic flow during walking, and was designed such that participants focused on idiothetic cues involved in walking. Please see [15,36] for similar manipulations designed to elicit idiothetic encoding.

### 2.2. Hypotheses

We aimed to test whether the absence of expected visual cues (in this case, an intersection) would affect how participants reconstructed the paths they took and thus would manifest in their pointing responses. Humans are sensitive to cardinal directions such as 90° in spatial tasks [5,29,37,38]. Since rich visual and idiothetic cues were provided in the walking phase for the 90°-angle corners on both sides of the hall (see Fig 2 and example videos), it was unlikely that participants over- or under-estimated the turning angle for the hallway navigation groups. Hence, the memory distortions would be more likely to come from the walking length estimation.

Since there were two path types, cross vs. no cross, and the main difference between them was whether there was an intersection (I; a crossing) between the first (L1) and the fourth segment (L4) of the path, it is possible that the cross paths were represented as their actual shapes (i.e., a crossed shape with an intersection). Alternatively, the cross paths could be represented as the other path type (i.e., a no-cross shape without any intersection). Particularly for the hallway navigation mixed-design group (Experiment 1a), participants were likely to be influenced by the mixed presenting order of the no-cross paths with the cross paths and thus tend to represent the cross paths as no-cross shapes. We term the first possibility the “cross to cross hypothesis” (Fig 1, “Cross to Cross (CtoC)”) and term the second possibility the “cross to no cross hypothesis” (Fig 1, “Cross to No cross (CtoN)”).

Similarly, for the uncrossed paths, it is also possible that the uncrossed paths were represented as their actual shapes (i.e., an uncrossed shape without any intersection). Alternatively, the uncrossed paths could be represented as the other path type (i.e., a crossed shape with an intersection). We term the first possibility the “no cross to no cross hypothesis” (Fig 1, “No cross to No cross (NtoN)”) and term the second the “no cross to cross hypothesis” (Fig 1, “No cross to Cross (NtoC)”).

These hypotheses are expanded upon below:

#### 2.2.1. Cross to cross hypothesis

One possibility is that participants’ responses would largely reflect distortions consistent with Euclidean (metric) axioms, as predicted by cognitive map theory [39–41], and might be expected for such rectangular trajectories. Therefore, participants would point within the vicinity of where they started (Fig 1, green arrows) and therefore maintain a crossed path as a crossed path in terms of where they remembered the start location to be. Such distortions are likely to include some variations due to execution noise (caused by actions like turning around) [26]. In this case, we would expect that participants would ignore the missing visual intersection when they crossed their path. The path’s topology remains the same in memory as in the physical space. For example, a 3.4 m long L1 may be represented as 3 m or even shorter such that the estimated start position (S’) is closer to the physically walked intersection than its actual position. Similarly, a 3.4 m long L4 may be represented as 3 m or even shorter such that the estimated end position (E’) is closer to the physically walked intersection than its actual position. If allowing some shortening in both the L1 and the L4 of the path and the existence of the intersection (I) in the representation, the path could be represented as a “crossed-shape path with an intersection” (Fig 1, “Cross to Cross (CtoC)”, red dashed diagram). Then, the pointing directions from the end (E) to the start (S) would be expected to be between the vectors I-S (90°) and E-I (180°; where I is the intersection between L1 and L4 according to physical geometry; Fig 1, “Cross to Cross (CtoC)”, the blue area; S2 Table), which includes the geometrical correct pointing direction. We refer to this possibility as the “Cross to Cross (CtoC)” hypothesis. This model would therefore involve “crossing” but would allow for some underestimation of L1 and L4 due to noise. This would be consistent with the idea that participants might shorten a path to varying degrees under normal conditions based on working memory errors yet maintain global geometry [1].

#### 2.2.2. Cross to no cross hypothesis

If not seeing the expected intersection distorted spatial memory, participants might point to a start location consistent with uncrossed paths when in fact the physically walked paths were crossed. The path’s topology will be different in memory. There are several ways that participants might do this. The most likely (and parsimonious) way would be mentally shortening either the first leg they walked (L1) or the fourth leg (L4), or both, with either one involving remember the start location significantly deviated from its “true” location in physically walked space. We propose three hypotheses (CtoN1, CtoN2, CtoN3) to illustrate this possibility.

*2*.*2*.*2*.*1*. *CtoN1*. The first hypothesis proposes that the participants would possibly underestimate L1 of the path but accurately represent L4 (Fig 1, “Cross to No cross (CtoN1)”). The estimated position of the start (S’) would be represented somewhere between the first turn (T1) and the intersection (I) along L1. Then the pointing directions would be more likely to fall in a range between the vectors E-I (180°) and E-T1 (S2 Table). It is also possible that the participants might overestimate L3, which would be effectively the same as underestimation of L1. We refer to both possibilities as “Cross to No cross 1 (CtoN1)” hypothesis.

*2*.*2*.*2*.*2*. *CtoN2*. The second hypothesis involves accurately representing L1 but underestimating L4; by mentally shortening L4, participants could also reconstruct the paths they walked to exclude crossing (Fig 1, “Cross to No cross (CtoN2)”). If this were to occur, then the estimated position of the end (E’) would be represented at somewhere between the third turn (T3) and the intersection (I) along L4. Then the pointing directions would be more likely to fall in a range between the vectors T3-S and I-S (90°; S2 Table). It is also possible that the participants overestimated L2, which would be effectively the same as underestimation of L4. We refer to both possibilities as “Cross to No cross 2 (CtoN2)” hypothesis.

*2*.*2*.*2*.*3*. *CtoN3*. It is possible that both L1 and L4 were underestimated in length, which also predicts a no-cross path shape (Fig 1, “Cross to No cross (CtoN3)”). If this were to occur, then the estimated position of the start (S’) would be represented at somewhere between T1 and the intersection along L1, and the estimated position of the end (E’) would be represented at somewhere between T3 and the intersection along L4. The pointing directions would be more likely to fall in a range between the vectors I-T1 (-90°) and T3-I (0°; S2 Table). It is also possible that the participants overestimated both L2 and L3, which would be effectively the same as underestimation of L1 and L4. We refer to both possibilities as “Cross to No cross 3 (CtoN3)” hypothesis.

#### 2.2.3. No cross to no cross hypothesis

For the uncrossed paths, it is possible to underestimate or overestimate some segments of the path to represent the path shape as not crossing (i.e., no intersection/crossing). Similar to the CtoC hypothesis, this hypothesis predicts that the pointing directions should include the geometric correct direction (Fig 1, green arrows). The path’s topology remains the same in memory as in the physical space. There are also a few ways that participants might do this. The most likely (and parsimonious) way would be mentally shortening either the first leg they walked (L1) or the fourth leg (L4), or both. To make the predicted pointing ranges different from each other and comparable to other predicted ranges, we propose two possibilities for the no cross to no cross hypothesis instead of combing them and proposing the combined predictions.

*2*.*2*.*3*.*1*. *NtoN1*. It is possible that participants would underestimate L1 (or overestimate L3) but accurately estimate L4 so that they would estimate the start (S’) to be closer to T1 along L1 to avoid any potential intersections between the extension of L4 and L1 (Fig 1, “No cross to No cross (NtoN1)”). The predicted pointing directions would be in the range between the vectors of E-T1 and the heading direction (0°; S2 Table). We refer to this possibility as “No cross to No cross 1 (NtoN1)” hypothesis.

*2*.*2*.*3*.*2*. *NtoN2*. Another possibility is that participants would under- or overestimate L4 so that they would estimate the end (E’) to be farther away from or closer to L1 along L4 (Fig 1, “No cross to No cross (NtoN2)”). The predicted pointing directions would be in the range between the vectors T3-S and I’-S (90°; where I’ is the intersection between L1 and L4 if L4 is extended; S2 Table). This range includes the geometric correct pointing direction. We refer to this possibility as “No cross to No cross 2 (NtoN2)” hypothesis.

#### 2.2.4. No cross to cross hypothesis

Although there was no missing visual information in the no cross paths, to understand what sorts of distortions might still occur under situations involving a matching of visual and idiothetic cues, it might still be possible that participants overestimated the fourth leg of the path so that they represent the uncrossed path as a crossed path, especially for the hallway navigation mixed-design group (Experiment 1a). The path’s topology will be different from the physical space. According to this hypothesis, L4 would be represented to be extended to cross L1 and there would be an intersection between L1 and L4 (Fig 1, “No cross to Cross (NtoC1)”). Since the maximum leg length in all paths was 3.4 m (S1 Table), the extended representation of L4 should be about 3.4 m long. Therefore, we hypothesize the maximum extension of L4 would be twice as long as E-I’ (where I’ is the intersection between L1 and L4 if L4 is extended), which makes the extended representation of L4 in the length of 3.4 m, 3.4 m, 4.25 m, and 2.55 m for each path (Path 5–8), respectively. Then the predicted pointing directions should be in the range between the vectors E’-S and I’-S (90°; S2 Table). It is also possible that the participants underestimated L2, which would be effectively the same as overestimation of L4. We refer to both possibilities as “No cross to Cross (NtoC1)” hypothesis.

#### 2.2.5. Summary of the hypotheses

The predictions for pointing directions are summarized in S2 Table and are illustrated with one path in Fig 1. For models, the predictions of the hypotheses for pointing directions are different and do not overlap with each other. For each path, the mean prediction for each hypothesis (Fig 1, blue arrows) was the circular mean across 1000 random angles within the predicted range.

### 2.3. Data analysis

Using circular statistics [42], we analyzed the response pointing direction data across trials for each path and then across participants for each condition. For each trial that expected a pointing direction towards the participant’s left side (i.e., the correct pointing direction was in a range from 180° to 360°; trials with right-turn paths), we transformed the response pointing direction (ranging from 0° to 360°) to a range from −180° to 180° in which 0° was their facing direction at the end of the path. The sign on these values was then flipped to ensure these trials were comparable with the corresponding not-flipped trials for the same path (i.e., left-turn paths). After flipping, we calculated the circular average of the response pointing direction across the four trials with the same path. Consequently, we obtained the response pointing direction for each path and each experimental condition for each participant. The signed pointing angular error according to Euclidean geometry (denoted as AE_G_) was calculated as (response direction–predicted direction) and was then transformed into a range from -180° to 180°.

After averaging the pointing directions across trials for each path and each participant, we excluded outliers of the pointing directions for each condition and group. For each group, the total number of data points were 4 (path shapes) × sample size for the group in this outlier detection procedure. C-statistics were calculated for each data point. The *C-statistic* for the *i*^th^ observation is (*r*_*-i*_—*r*)/r, where *r* is the mean resultant length for all observations (i.e., length of mean vector; *r* = |the resultant vector of all observations|/sample size) and *r*_*-i*_ is the mean resultant length for all observations except the *i*^th^ one. The C-statistic indicates the discordancy of circular data [43,44]. A larger value of C-statistic indicates that the data point is further away from the mean and more likely to be an outlier. The distribution of the C-statistic greatly depends on the variance of the sample which is indicated by the statistic *kappa* (i.e., the parameter of concentration). A larger kappa value means smaller variance of the sample data; 1/kappa is related to angular standard deviation [42]. If kappa = 0, the distribution is uniform; if values for kappa are large, the distribution approaches a normal distribution.

To find the critical value for the C-statistics in order to detect potential outliers and better approximate a circular normal (von Mises) distribution (which was an assumption of some of the statistical tests used here), we created a simulation program in R (see S1 File). This program uses three parameters (total number of data points, kappa, probability) to simulate a distribution of c-statistics and find a cut-off value. Specifically, the program generated 1000 random samples with a mean of 0, the total number of data points (which depended on the sample size of this group), and the kappa value (which is the estimated kappa of this group) and returned a distribution of the maximum of C-statistics in each of the 1000 samples. We determined a cut-off value of C-statistics using a probability of 90%. We used the “rounded-up” total number of data points of each group for simulation (n = 150 for the two hallway groups with 128 or 124 total data points, 120 for the pole-guided group with 84 total data points). The kappa values used in the simulation were also larger than the observed value since the observed kappa was likely to be underestimated with potential outliers (Table 2). The kappa value used in the simulation was determined by taking the difference between this value and the returned kappa value after exclusion. A kappa value for a stimulation would be acceptable if a) this difference was smaller than 1.5, b) the observed kappa after excluding outliers was improved from the original value, and c) the excluded data points were less than 10% of the total data.

**Table 2:** Outlier proportions and kappa values in outlier detection procedure in Experiments 1–3.

Experiment	Condition	Observed kappa before exclusion	Kappa used in simulation	Observed kappa after exclusion	Outlier datapoints number	Outlier proportion
1a (hallway mixed-design)	Hidden intersection Cross (HI-C)	0.51	0.7	0.74	10	7.81%
	No intersection No cross (NI-NC)	1.71	3.5	2.04	5	2.93%
1b (pole-guided)	Pole-guided cross (PG-C)	2.33	4	2.54	2	2.38%
	Pole-guided No cross (PG-NC)	1.03	1.8	1.34	7	8.33%
1c (hallway blocked-design)	Hidden intersection Cross (HI-C)	0.74	0.75	0.91	6	4.84%
	No intersection No cross (NI-NC)	1.32	2.1	1.66	7	5.65%
2	Hidden intersection Cross (HI-C)	1.16	2	1.44	7	5.47%
	True intersection Cross (TI-C)	1.38	2	1.67	6	4.69%
	No intersection No cross (NI-NC)	7.04	9	7.04	0	0
	False intersection No cross (FI-NC)	7.78	9	8.52	1	0.59%
3	True intersection Cross (TI-C)	1.87	3.2	2.16	4	3.45%
	False intersection No cross (FI-NC)	7.45	8	7.45	0	0

The outlier detection results are shown in Table 2. For details about this outlier detection procedure, see S1 Text and S4 Fig. We return to the issue of outliers later in the results / discussion. In addition, we also analyzed the conditions in which the outlier proportion was greater than 5% without outliers removed and found comparable results to those with the outliers removed (see S1 Text and S5 Fig).

Using the observed pointing directions, we can distinguish the hypotheses from each other for each condition. Here we used a Bayesian regression method based on previous research [45,46]. Specifically, with the pointing angles as the circular response for each hypothesis, we constructed a Bayesian projected normal mixed-effect model (pointing angle ~ hypothesis + subject). The circular mixed-effect model was then estimated via the function *bpnme* in the R package *bpnreg* [47]. We used this function to run a Bayesian Markov Chain Monte Carlo (MCMC) sampler with 1000 iterations for the mixed-effect model to estimate the pointing angles for each hypothesis. With the fitted regression model, we calculated the posterior estimates of the pointing angles for each hypothesis, the circular mean, and 95% higher posterior density (HPD) interval of the pointing angles. Then we used the 95% HPD interval to compare with the predicted pointing range in each hypothesis (S2 Table) and calculated an “overlapping” ratio (range from 0 to 1) for each predicted range. The larger the ratio is, the better the fit of the hypothesis.

We report the regression results (linear coefficients and random effects) in S4 Table. In the regression, pointing angles were decomposed from a vector into sine and cosine components in a two-dimensional coordinate system (Component I and II). The fitted circular regression model showed the linear coefficients for the two components separately. The interpretation of the regression results by the two-component structure is similar to that in the GLM model [45,46]. The scripts behind this analysis have been made publicly available at the Open Science Framework (https://osf.io/3aygm/).

Non-parametric Mardia-Watson-Wheeler tests were applied to compare the circular errors (AE_G_) between groups. All statistical tests were two-tailed with *α* = .05.

### 2.4. Results

**Fig 3:**
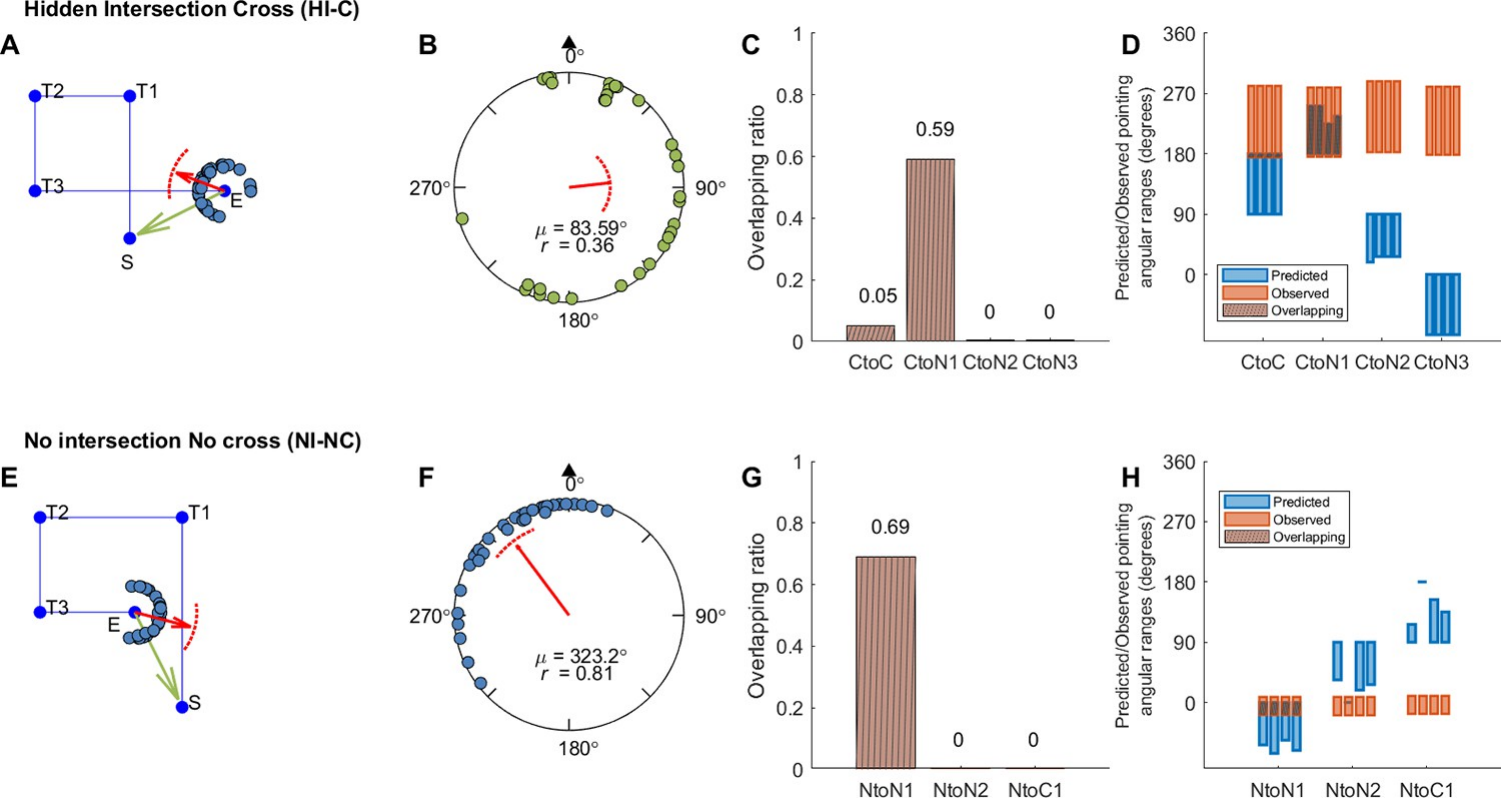
Response pointing directions and hypothesis comparison results in Experiment 1a (hallway mixed-design group). Panel A, E: Example pointing directions in one path (A: Path 3; E: Path 5). Each dot indicates the pointing direction of one participant across trials for the path. The red arrow indicates the circular mean direction of the pointing directions across all participants. The arc above the mean direction indicates the 95% confidence interval of the mean direction. Green arrows indicate the correct pointing direction according to Euclidean geometry. S: Start. T1-3: Turn 1–3. E: End. Panel B, F: Mean pointing error (*μ*; AE_G_) across paths for each condition. The black triangle indicates the pointing direction consistent with Euclidean geometry (0°). *r* is the mean resultant length of all pointing angles. Panel C, G: Mean overlapping ratio between observed and predicted pointing ranges across paths for each hypothesis (Fig 1, S2 Table). Panel D, H: Predicted or observed pointing angular ranges (in degrees) for individual paths. Each blue/orange bar shows the result for one path. Blue bars indicate the predicted ranges according to each hypothesis. Orange bars indicate the estimated 95% highest posterior density (HPD) interval according to the 1000 iterations for the mixed-effect model with the observed pointing angles (see Section 2.3). Brown shaded areas show the overlapping intervals.

**Fig 4:**
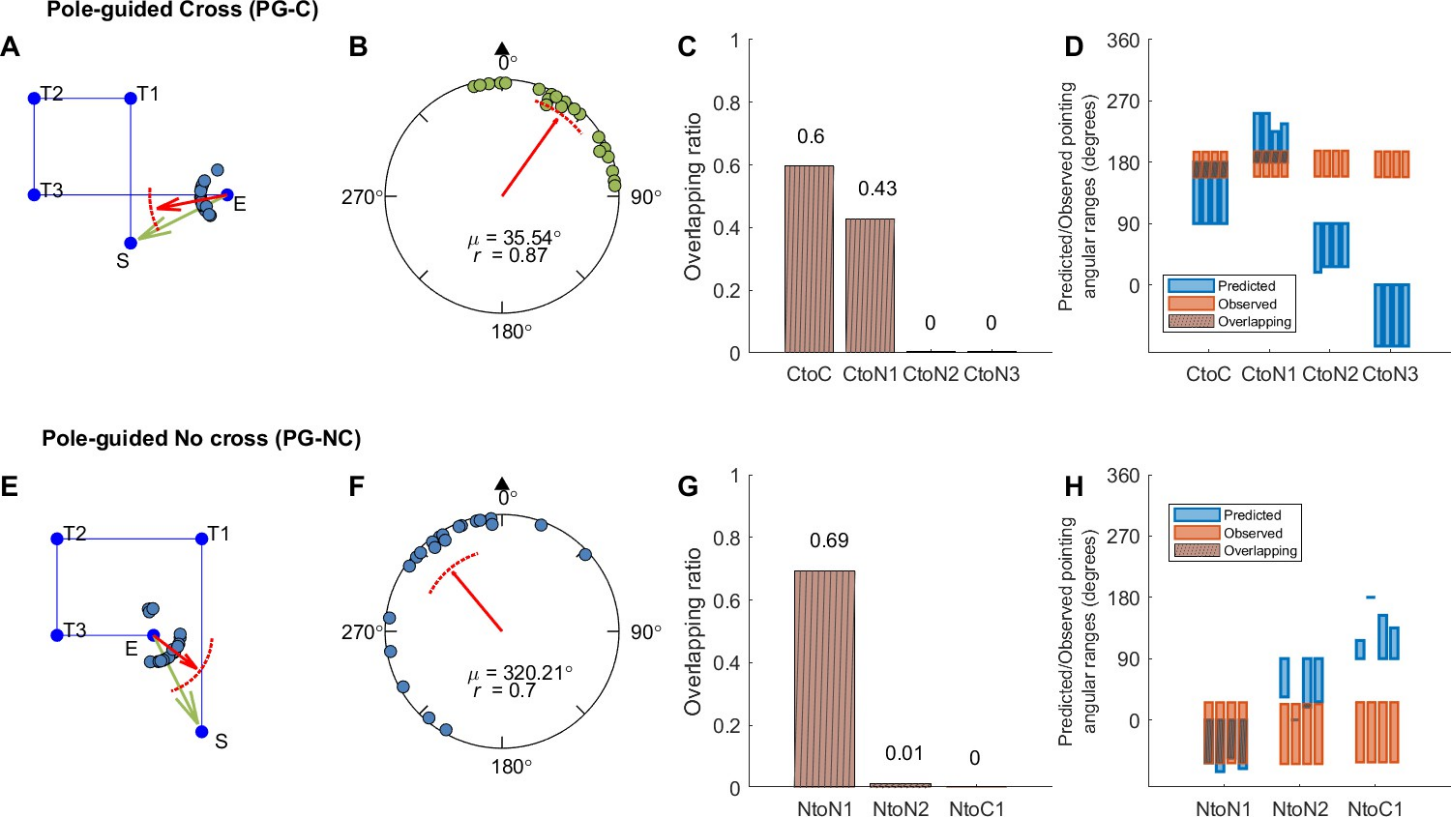
Response pointing directions and hypothesis comparison results in Experiment 1b (pole-guided group). Panel A, E: Example pointing directions in one path (A: Path 3; E: Path 5). Panel B, F: Mean pointing error (AE_G_) across paths for each condition. Panel C, G: Mean overlapping ratio between observed and predicted pointing ranges across paths for each hypothesis (Fig 1 and S2 Table). Panel D, H: Predicted or observed pointing angular ranges (in degrees) for individual paths. Abbreviations and legend are the same as in Fig 3.

**Fig 5:**
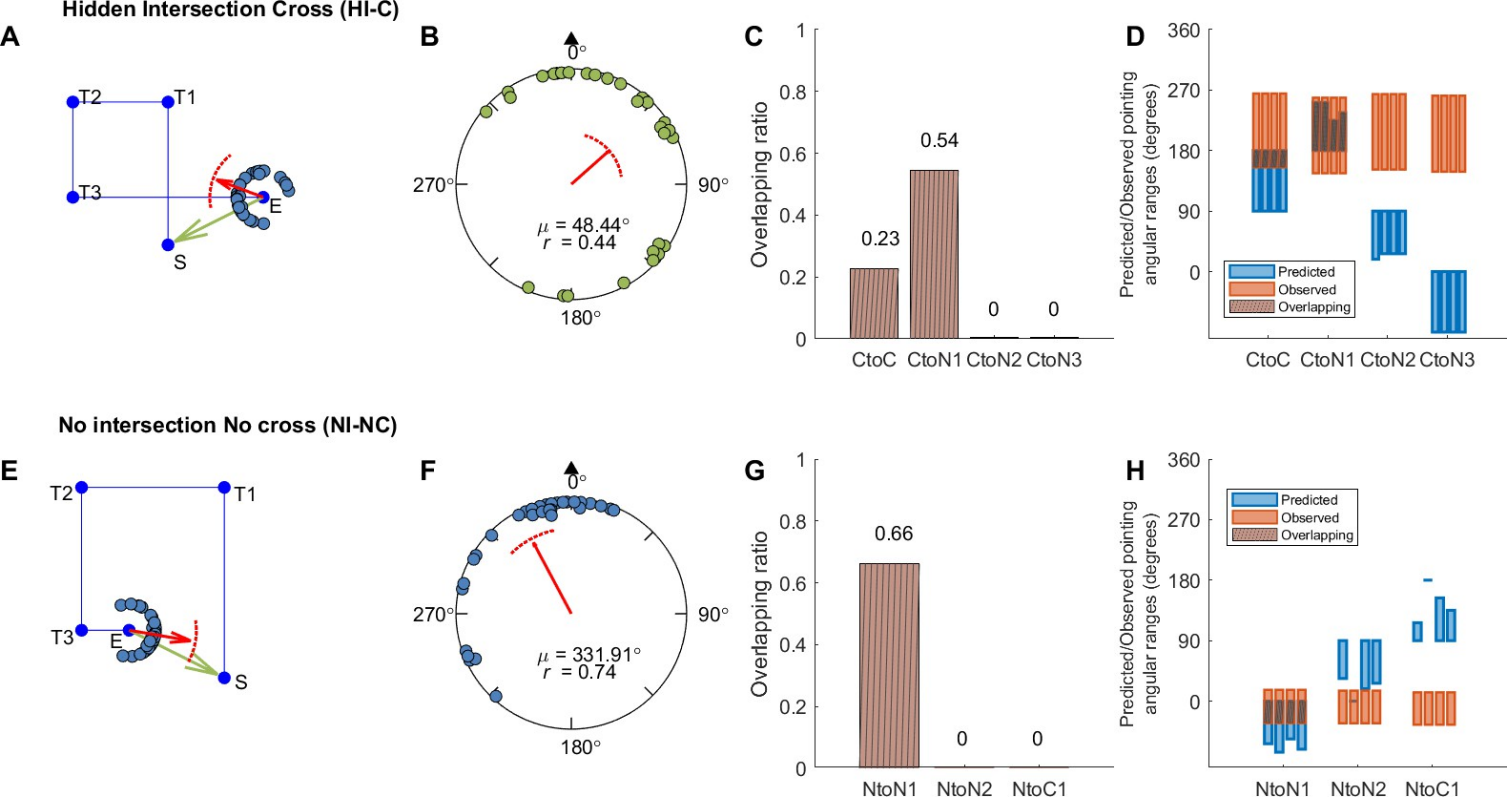
Response pointing directions and hypothesis comparison results in Experiment 1c (hallway blocked-design group). Panel A, E: Example pointing directions in one path (A: Path 3; E: Path 7). Panel B, F: Mean pointing error (AE_G_) across paths for each condition. Panel C, G: Mean overlapping ratio between observed and predicted pointing ranges across paths for each hypothesis (Fig 1, S2 Table). Panel D, H: Predicted or observed pointing angular ranges (in degrees) for individual paths. Abbreviations and legend are the same as in Fig 3.

The response pointing directions for each path and condition are shown in Figs 3A, 3E, 4A, 4E, 5A and 5E, and S1–S3 Figs. We first aimed to determine whether not rendering the visual intersection where it was expected would impact how participants remembered the start location. To do this, we compared pointing in the cross condition between the hallway mixed-design group, who saw hallways but no intersection (Fig 3A and 3B), and the pole-guided group, who simply saw poles guiding their navigation but walked identical paths (Fig 4A and 4B). Notably, the average pointing angle for participants in the “hidden intersection cross” (HI-C) condition was significantly deviated from the correct pointing direction and towards the other side of the expected intersection position on Leg 1, with participants pointing, on average, 83.59° to the clockwise right of their physical start location (Fig 3A and 3B; 95% CI = [41.51°, 125.68°]). In contrast, without visual information, but for the same walked paths (the “pole-guided cross” condition, PG-C; Table 1), participants pointed on average 35.54° to the clockwise right of the start location (Fig 4A and 4B; 95% CI = [19.11°, 51.97°]), suggesting a lower distortion and that the visual manipulation (presence or absence of hallways) had an impact on pointing. This difference was also notable on individual paths when comparing HI-C and PG-C conditions (S1 Fig). Note that responses in the no cross conditions (the “no intersection no cross” (NI-NC) and “pole-guided no cross” (PG-NC) conditions), were also closer to the physical location of their starting point, as in the PG-C condition (NI-NC: mean = 323.2°, 95% CI = [308.58°, 337.83°]; PG-NC: mean = 320.21°, 95% CI = [297.55°, 342.88°]; both closer to 0°/360°; Figs 3E, 3F, 4E and 4F).

To compare the responses between the hallway and pole-guided groups, we examined the angular error (AE_G_) by conducting two-sample Mardia-Watson-Wheeler tests for each condition (cross/no cross; Figs 3B, 3F, 4B and 4F). We found that there was a significant angular difference in pointing responses between the two groups for the cross condition, *W* = 12.66, *p* = .002, but not for the no cross condition, *W* = 0.07, *p* = .966. This further supported the idea that hiding the intersection influenced participants’ memory for the location of the start point within the hallway. In contrast, without the visual hallways, or in non-intersecting paths, participants were significantly closer to their physical start location of 0°. For the analysis about absolute AE_G_, see S1 Text and S6 Fig.

While comparing the hallway with the pole-guided groups and cross with no cross conditions suggested greater memory distortions overall, whether this might have involved remembering uncrossed paths despite having walked crossed paths (or vice versa) remained unclear. To determine which of our hypotheses (Fig 1, S2 Table) fit the observed pointing directions in each condition (see Section 2.3), we conducted a Bayesian projected normal regression model (pointing angle ~ hypothesis + subject) for each condition and each group. The linear coefficients for Component I and II are shown in S3 Table. The random effect variances are shown in S4 Table.

With the fitted regression model, we then compared the 95% HPD interval for each hypothesis with the predicted angular range and calculated the overlapping ratio for each hypothesis and each path. Mean ratios across paths were calculated for each hypothesis (Figs 3C, 3D, 3G, 3H, 4C, 4D, 4G and 4H). For the hidden intersection—cross (HI-C) condition in which no visual intersection was shown to participants while walking, the observed pointing direction was most consistent with the prediction of Cross to No cross (CtoN1) hypothesis (mean ratio = 0.59). This suggests that participants likely employed a representation in which they significantly underestimated L1 such that it was shortened to not intersect with L4 (Fig 1, “CtoN1”). In contrast, for the pole-guided cross (PG-C) condition involving the same paths, the observed data was most consistent with the prediction of Cross to Cross (CtoC) hypothesis (mean ratio = 0.60). This suggests that this hypothesis captured the patterns for the PG-C condition, indicating some distortion, possibly due to noise, for L1 and L4, but keeping the intersection in the representation (Fig 1, “CtoC”). Similarly, for the no cross conditions, the observed pointing direction was most consistent with the prediction of No cross to No cross (NtoN1) hypothesis (mean ratio = 0.69) for the no intersection uncrossed hallway (NI-NC) group while the observed data was also consistent with the prediction of NtoN1 hypothesis (mean ratio = 0.69) for the pole-guided no cross (PG-NC) group (Fig 1, “NtoN1”). These findings again suggest some possible memory distortions in terms of underestimation of L1 but less than what was observed for the HI-C condition in which no intersection was visually presented.

We also found greater pointing variability in the hidden intersection—cross (HI-C) condition compared to the other conditions (NI-NC, PG-C, PG-NC), again, suggesting the influence of the missing intersection. As indicated by the observed length of vector *r* and the kappa values (Figs 3–5, Table 2), we found that the HI-C condition showed more dispersed (variable) responses across participants whereas the other three conditions showed less uniform responses (*r* = .36 for HI-C vs. .81, .87, and .70 for NI-NC, PG-C, and PC-NC, respectively; kappa = 0.74 for HI-C, 2.04, 2.54, 1.34 for NI-NC, PG-C, and PC-NC, respectively; Table 2). To examine the consistency of pointing, Rayleigh tests were conducted for each condition and each group. The results showed that the pointing directions in all four conditions were not uniformly distributed (*p*s < .02). Thus, despite greater pointing variability, responses in the HI-C condition were significantly non-uniform in the pointing pattern, further suggesting that the visual manipulation of hiding the intersection influencing participants’ memory for the start location was not due to disorientation alone.

It is possible that the increase in pointing variability for participants and higher number of outliers in the HI-C condition was not due to increased disorientation but due to the randomization of the conditions. In other words, one condition may have influenced how participants learned the condition immediately after it. For example, after walking a few no intersection—no cross (NI-NC) hallways, participants might have become less sensitive to detect the missing intersection in a crossed hallway in HI-C condition. To attempt to address this issue and test the robustness of our findings, we conducted Experiment 1c which was a separate replication study involving only the HI-C and NI-NC conditions but in a blocked design instead of a randomized/mixed design (Fig 5). For the HI-C condition, participants again pointed to a start location that was away from the intersection location (48.44° deviated clockwise right from the start location; 95% CI = [15.24°, 81.64°]). For the NI-NC condition, the pointing directions again were closer to the correct pointing direction (mean = 331.91°, 95% CI = [314.91°, 348.90°]). Comparing the blocked-design group with the mixed-design hallway group, the confidence intervals overlapped for both the cross and no cross conditions, suggesting overall similar results for the two groups. Rayleigh tests again showed the pointing responses were not uniformly distributed (*p*s < .003). As indicated by the observed length of vector *r* and the kappa values (Table 2), we found more consistent responses in the NI-NC condition than in the HI-C condition. These findings suggest that disorientation did not overly confound our findings in Experiment 1a and suggest a replication of our basic effect showing that the missing intersection, which otherwise would be expected, significantly impacted how participants remembered their paths.

We also performed the circular regression model for this group and compared the 95% HPD intervals with the predicted angular ranges. The observed pointing direction in the hidden intersection—cross (HI-C) condition was most consistent with the prediction of Cross to No cross (CtoN1) hypothesis (mean ratio = 0.54). For the no intersection—no cross (NI-NC) condition, the observed pointing angle was most consistent with the prediction of No cross to No cross (NtoN1) hypothesis (mean ratio = 0.66). These results are overall consistent with the mixed-design hallway group (Experiment 1a) showing that the HI-C condition resulted in greater deviations from the correct pointing directions than the NI-NC condition, although the effect was not as dramatic as when the conditions were completely randomized in Experiment 1a.

### 2.5. Discussion

Experiment 1 revealed that not visually perceiving an expected intersection in a four-segment hallway (HI-C condition) resulted in significant distortions of the experienced (walked) geometry. Participants, on average, pointed to a start location that would be farther away from the intersection they would have expected based on the path they walked. Our modeling suggested the possibility that this distortion involved shortening one leg of the hallways to accommodate for this violation in expectation, creating an uncrossed path from a crossed one. To ensure that these effects were not somehow driven by disorientation, we replicated the same basic findings in a blocked-design version of Experiment 1a using an independent sample of participants. Notably, none of the model fits was perfect (i.e., equal to 1). In the case of the mismatching condition (HI-C), this suggests that idiothetic cues from the walked path likely exerted some influence on their remembered start location. For the other conditions (NI-NC and pole-guided conditions), this suggests that some memory distortions were still present, possibly due to noisy path integration estimates.

**Fig 6:**
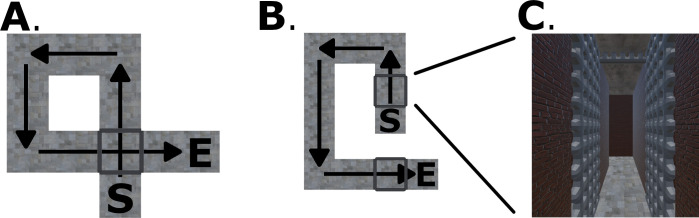
Example paths in Experiment 2 for the (A) true intersection cross (TI-C) and (B) false intersection no cross (FI-NC) conditions. (C) shows a leg of the hallway with the obscuring gates at a false intersection from the participant’s perspective.

One issue is that all crossed path conditions in Experiment 1 involved a more backward-oriented correct pointing direction (> ±90°), whereas all uncrossed paths involved pointing toward a more forward-oriented direction (±0° to 90°). Previous research suggests that the distribution of pointing errors in some participants may have a forward-facing bias, which, in our experiment, could possibly create the appearance of distorted memories for paths [29]. To exclude any possible pointing-forward bias in the no cross condition, we modified the paths in Experiment 2 to make the two conditions more comparable. The predicted pointing directions and pointing distances were matched in each pair of cross and no-cross paths. Additionally, because participants did not see an intersection in the hidden intersection condition (HI-C) but only experienced this “node” as a based on physically crossing paths, it is possible they did not encode this as a node. In this case, it is unclear whether the findings for HI-C condition are completely consistent with the predicted memory distortions as in an uncrossed path. Importantly, we note that the hidden intersection condition showed greater distortions than walking the same path but with idiothetic cues only and no visual hallways in the corresponding pole-guided condition; the pointing patterns between these conditions also differed. Nonetheless, an important test of whether such distortions occur in memory involves whether we can show a gain-of-function effect based on connecting visual nodes that are not otherwise physically connected. In other words, when faced with visually rendered “false” intersections (Fig 6B and 6C) in an uncrossed hallway (as the NI-NC condition), could we create the illusion that participants in fact crossed their paths?.

## 3. Experiment 2

### 3.1. Methods

#### 3.1.1. Participants

We recruited 32 participants (21 female, 11 male) from the University of Arizona undergraduate Psychology program and surrounding Tucson city area whose ages ranged from 18 to 36 years old with a mean age of 20.81. All participants received either course credit for their participation or were compensated at a rate of $20 per hour. All participants had normal or corrected-to-normal color vision, normal or corrected-to-normal hearing, and reported no history of cardio-vascular problems or motion sickness. Written consent was obtained before the experiment, and the methods were approved by the Institutional Review Board (IRB) at the University of Arizona.

#### 3.1.2. Apparatus

The apparatus matched Experiment 1.

#### 3.1.3. Design

All participants were in the hallway navigation group, not in the pole-guided group. Eight new intersecting and eight new non-intersecting hallway models in the experimental program were created (S7 Fig, S5 Table). They matched the same general layout as those used in Experiment 1. However, the total path lengths, pointing angle to the start location from the end location, and Euclidean end-to-start distances were matched for each pair of the paths. For example, Path 1 was the same as Path 5 in terms of the path length and end-to-start distance, and the left-turn version of Path 1 had the same correct pointing angle as the right-turn version of Path 5.

There were four conditions in this experiment, creating a 2 (Intersection visibility: hidden/no intersection, shown true/false intersection) × 2 (Path type: cross, no cross) within-subject design (Table 1), all of which involved visually rendered hallways on the HMD. For half of the trials, the hallways were presented as described in Experiment 1 (HI-C and NI-NC conditions). For the other half, the visibility of the intersections was manipulated. Specifically, for the visibly intersecting hallways, the intersection was presented to the participants during walking, with a grated gate being added to each direction of the intersection (Fig 6). The gates were shown in an “open” position along the current walking direction and a “closed” position for the orthogonal directions. In this condition (“true intersection cross”, TI-C), the participant experienced the intersection as they would in the real world. We included this as a control condition (not present in Experiment 1) that included the same crossed path as the hidden intersection–cross condition (HI-C) but involved rendering the “true” intersection that otherwise should have been present. For the non-intersecting hallways, two false intersections were shown in the first and the fourth legs of the hallway (“false intersection no cross”, FI-NC condition). They served no function (other than as an experimental manipulation) because they were not connected to any other parts of the hallway. In total, each participant experienced 16 trials from each condition in a mixed, randomly determined order. The practice portion was the same as that for the hallway navigation groups in Experiment 1a and 1c.

#### 3.1.4. Procedure

The procedure was almost the same as Experiment 1, except for a few changes. First, the hallway always disappeared when participants were asked to point. This was done to eliminate any possible orientation cues. Second, during the pointing task, after participants had reached the end of the fourth hallway, participants were asked to point to their start position with a virtual laser pointer. The landing position (i.e., the landing coordinates in the virtual environment) of the laser pointer was recorded, providing a measure of both distance and direction of the remembered start location relative to the end point. Third, a confidence rating task was added after the pointing task on each trial. The participants were asked to rate their confidence for their pointing accuracy by adjusting the length of a dark-grey rectangular bar in respect to a separate light-grey bar. The longer the dark-grey bar, the more confidence expressed, and vice versa. The ratio between the dark-grey bar and the light-grey bar was the confidence rating score (ranging from 0 to 1), with the initial value being set to 0.5 (see example videos). For brevity, the results for confidence rating scores were presented in S1 Text and S8 Fig.

### 3.2. Hypotheses

For the hidden intersection—cross (HI-C) and true intersection—cross (TI-C) conditions, we again tested the four hypotheses as in Experiment 1: Cross to Cross (CtoC), Cross to No cross (CtoN1, CtoN2, CtoN3; Fig 1). The predictions according to each hypothesis were modified according to the adjusted path dimensions (S5 Table) and are detailed in S2 Table. Again, there was no overlap between the predicted pointing ranges of the hypotheses.

**Fig 7:**
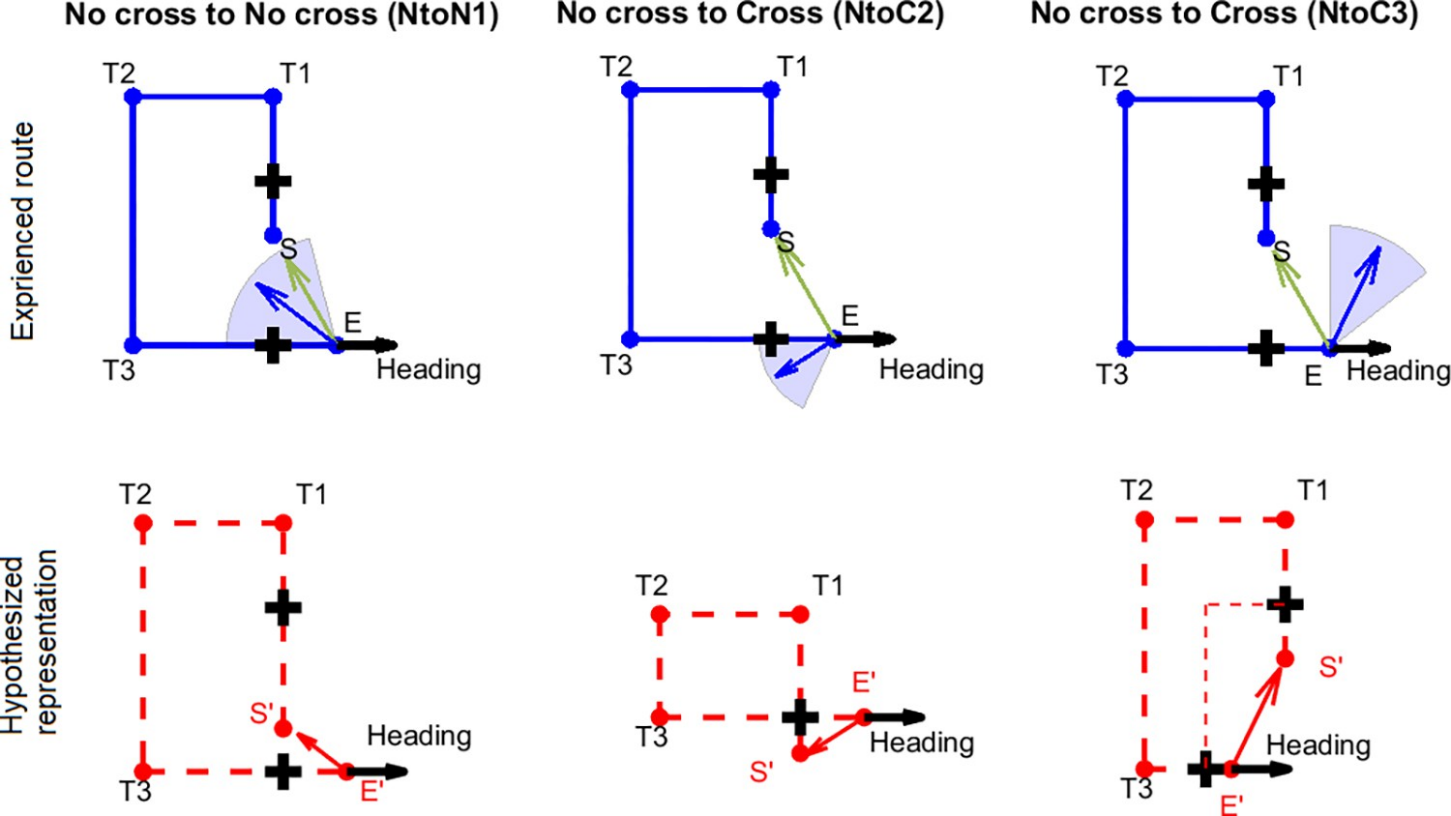
Predicted pointing directions according to each hypothesis for the FI-NC condition in Experiments 2 and 3. The example path is Path 6. Black bold pluses indicate the intersections. Thin red dashed lines (bottom right panel) indicate the extra represented path segments. Other abbreviations and legend are the same as in Fig 1.

The particular focus for Experiment 2 was the false intersection—no cross (FI-NC) condition, which involved adding two false intersections (Fig 7, bold black pluses) to an uncrossed path. For this condition, we proposed three hypotheses. We again tested the No cross to No cross 1 hypothesis (NtoN1) from Experiment 1 with the modification that the L1 was either extended or shortened due to noise. The predicted pointing ranges have been adjusted according to the path dimensions in Experiment 2 (S2 Table, Fig 7, “No cross to No cross (NtoN1)”). Note that the predicted range for the NtoN1 hypothesis includes the geometric correct direction (Fig 7, green arrows). The path’s topology remains the same in memory as in the physical space. In addition, we propose two new hypotheses that both predict a representation of a crossed path for an uncrossed path with false intersections.

#### 3.2.1. No cross to cross 2 (NtoC2)

Since the two intersections were identical, one possibility is that participants would treat the intersections they saw as the same one and use it as an anchor to memorize the path. This would result in a crossed path similar to the true intersection—cross (TI-C) condition, which involved the crossed path but with the intersection shown at its actual crossing location. The path’s topology will be different from the physical space. If participants treated the false intersection—no cross (FI-NC) condition as if the intersection was simply moved forward or backward along L1, participants would underestimate L3 of the path to create a single intersection (i.e., the two intersections were represented as “stacked” with each other) and L4 would be represented as intersecting with L1 (Fig 7, “No cross to Cross (NtoC2)”). In this case, their pointing directions would be more likely to fall in a range between the vectors E’_far_-S and 180° (where E’_far_ is the intersection point of the extended L2 and the orthogonal line through E, i.e., the x coordinate of E’_far_ is the same as E and y coordinate is the same as T1; S2 Table, S9 Fig). It is also possible that the participants overestimated L1 so that L1 intersected with L4, which would be effectively the same as underestimation of L3. We refer to both possibilities as the “No cross to Cross 2 (NtoC2)”.

#### 3.2.2. No cross to cross 3 (NtoC3)

This hypothesis, like NtoC2, involves violations of topology but incorporates the idea that participants might create an illusory crossed path by creating a fifth auxiliary hallway. Accordingly, because participants did not walk the orthogonal directions of the intersections (and only viewed them with a glimpse in passing), they might instead treat the intersections as different from one another and might be likely to believe they were connected by an unexperienced path segment (Fig 7, “No cross to Cross (NtoC3)”, thin red dashed lines). Although such connections are physically impossible according to Euclidean geometry (since the false intersections [F1 and F2 for brevity] were always aligned with L1, Fig 7), it would be possible for the participants to believe this if they represented the intersection’s position on L4 of the path inaccurately. According to this hypothesis, their pointing directions would be more likely to fall in a range between the vectors F2-S (-90°) and T3-S (where F2 is the false intersection on L4; S2 Table). It is also possible that the participants overestimated L2, which would be effectively the same as underestimation of L4. We refer to both possibilities as the “No cross to Cross 3 (NtoC3)” (Fig 7, “NtoC3”).

For the no intersection–no cross (NI-NC) condition, similarly, we fit the data with the three hypotheses as the FI-NC condition (NtoN1, NtoC2, NtoC3). Since there was no geometric violation based on matching of idiothetic and visual cues, we predicted that responses in this condition would be most likely consistent with the NtoN1 hypothesis.

### 3.3. Data analysis

The exclusion criteria and analysis procedures were the same as in previous experiments (see Section 2.3 and Table 2) except that the pointing responses in the left-turn no cross paths (i.e., Path 5–8 in FI-NC and NI-NC; instead of those in right-turn paths in Experiment 1) were flipped due to the modified path shapes in these conditions. Hotelling’s paired tests were applied to compare two conditions of circular errors for the same group of participants. All statistical tests were two-tailed with *α* = .05.

### 3.4. Results

**Fig 8:**
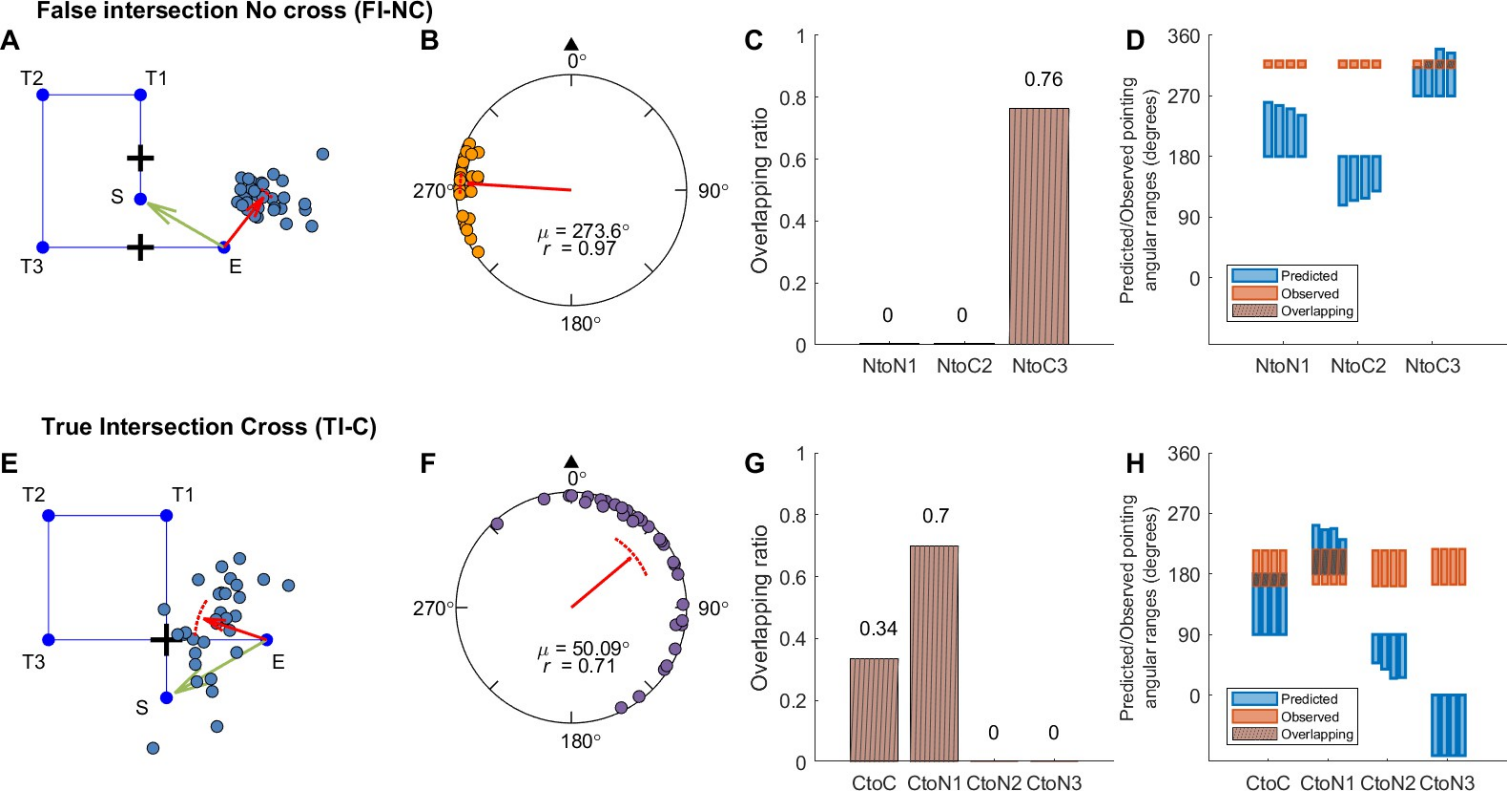
Response pointing directions and hypothesis comparison results in Experiment 2 (FI-NC and TI-C conditions). Panel A, E: Example pointing locations in one path (A: Path 8; E: Path 4). The red arrow indicates the circular mean direction and the distance of the pointing across all participants. Panel B, F: Mean pointing error (AE_G_) across paths for each condition. Panel C, G: Mean overlapping ratio between observed and predicted pointing ranges across paths for each hypothesis (Fig 7, S2 Table). Panel D, H: Predicted or observed pointing angular ranges (in degrees) for individual paths. Abbreviations and legend are the same as in Fig 3.

**Fig 9:**
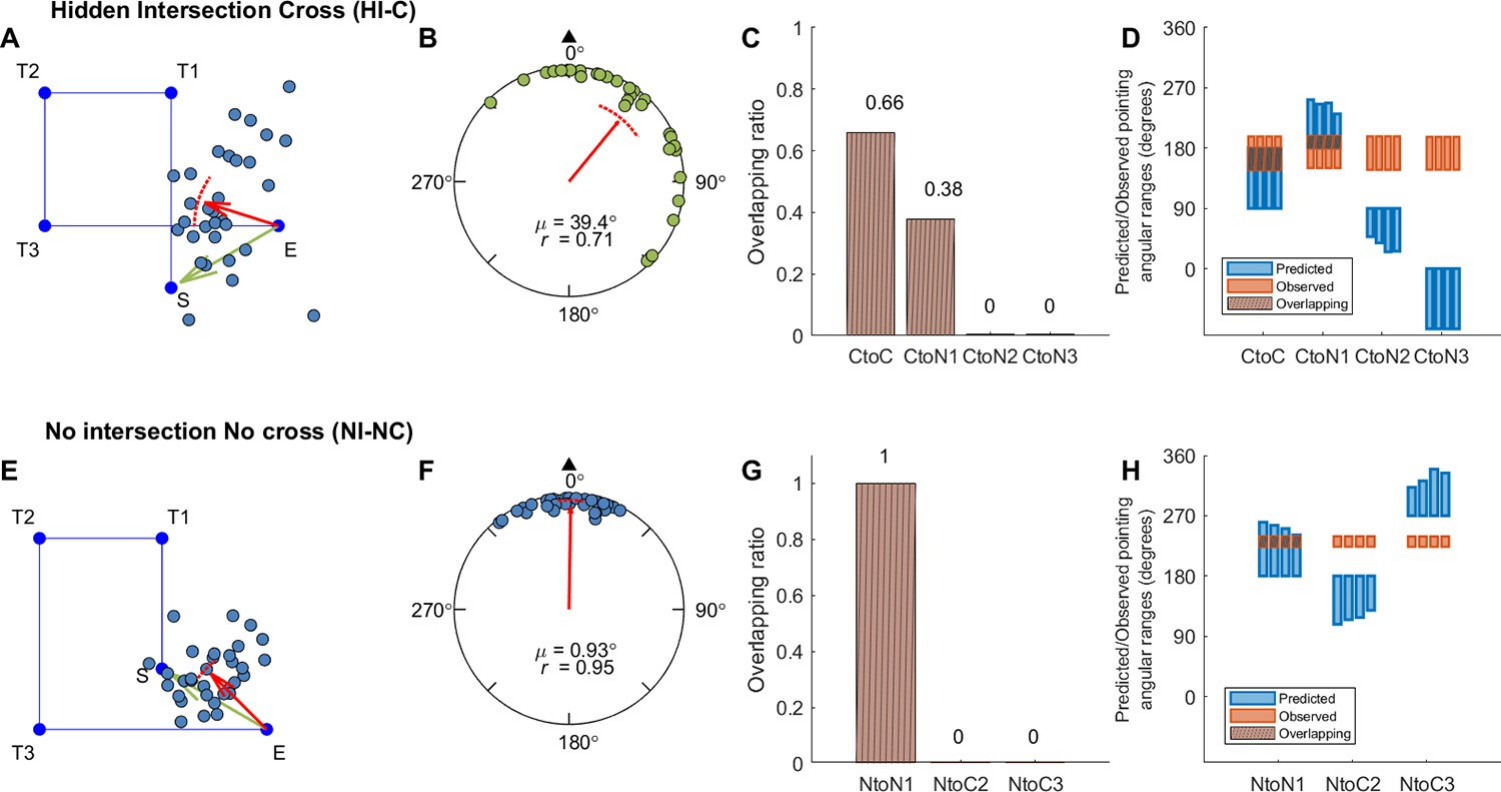
Response pointing directions and hypothesis comparison results in Experiment 2 (HI-C and NI-NC conditions). Panel A, E: Example pointing directions in one path (A: Path 4; E: Path 8). The red arrow indicates the circular mean direction and the distance of the pointing across all participants. Panel B, F: Mean pointing error (AE_G_) across paths for each condition. Panel C, G: Mean overlapping ratio between observed and predicted pointing ranges across paths for each hypothesis (Fig 7, S2 Table). Panel D, H: Predicted or observed pointing angular ranges (in degrees) for individual paths. Abbreviations and legend are the same as in Fig 3.

We first focused on the “false intersection no cross” (FI-NC) and “true intersection cross” (TI-C) conditions (Table 1), as these conditions were not tested in any of our previous experiments. Specifically, FI-NC condition allowed us to determine whether showing a false intersection would result in distortions of participants’ memory for paths, while the TI-C condition served as a situation in which visual and idiothetic cues matched because the intersections were rendered where they should be. The response pointing directions and locations for each path were shown in Figs 8A, 8E, 9A and 9E and S7 Fig. Note that unlike Experiment 1, both pointing direction and distance were plotted. In the FI-NC condition, which was a critical test of our hypothesis regarding memory reconstructions of walked paths based on false intersections, we found that participants pointed at 273.6° counterclockwise left to the correct direction (95% CI = [268.13°, 279.07°]; Fig 8B), significantly deviated from 0°/360°.

To determine whether the manipulation of the false intersections affected participants’ memory for the paths they walked in the FI-NC condition, we conducted Hotelling’s paired tests for AE_G_ (Figs 8B, 8F, 9B and 9F). We first compared the FI-NC and TI-C conditions because in both conditions, participants saw and walked through the “intersection” twice. There was a significant difference in pointing for the FI-NC compared to the TI-C conditions (*F*(1, 30) = 397.8, *p* < .001), suggesting that participants treated these conditions differently. We also compared with NI-NC condition, in which no intersections were shown but involved the same uncrossed path and was identical to the NI-NC condition in Experiment 1. We also found a significant difference between FI-NC and NI-NC (*F*(1, 30) = 1001.151, *p* < .001; NI-NC: mean = 0.93°, 95% CI = [354.30°, 7.56°]; Fig 9F). These findings further support the idea that rendering the “false” intersections affected participants memories for the paths they walked.

As in Experiment 1, we conducted a Bayesian projected normal regression model (pointing angle ~ hypothesis+ subject) for each condition and examined which of our hypotheses (Fig 7, S2 Table) fit the observed pointing directions in each condition. The linear coefficients for Component I and II are shown in S6 Table. The random effect variances are shown in S4 Table. For the false intersection no cross (FI-NC) condition, the observed pointing directions were consistent with the prediction of the No cross to Cross (NtoC3) hypothesis as indicated by the overlapping ratio (mean ratio = 0.76; Fig 8C and 8D). This suggests one possible way in which participants remember the shapes of the paths was with L4 as shorter than the actual final leg that they walked, with the two intersections as distinct but connected (Fig 7, “NtoC3”). This would involve the first intersection doubling back to the second intersection, and therefore that they may have crossed an unseen extraneous hallway they had not walked. Importantly, they could not have experienced this auxiliary hallway based on the axioms of Euclidean geometry because it would involve taking two nodes (the final intersection and their end point) and compressing their distance past zero.

For the true intersection—cross (TI-C) condition, the observed pointing direction was more consistent with the prediction of the Cross to No cross (CtoN1) hypothesis (mean ratio = 0.70) but also was partially consistent with the Cross to Cross (CtoC) hypothesis (mean ratio = 0.34; Fig 8G and 8H). Surprisingly, this suggests a memory distortion involving possibly shortening L1 in the TI-C condition, which may have been due to an unanticipated effect of the hidden intersection—cross condition (HI-C) on the true intersection–cross condition (TI-C), an issue we explore in more depth shortly.

As plotted in Fig 9C and 9D, for the hidden intersection—cross (HI-C) condition (replication from Experiment 1a and 1c, i.e., hallway navigation cross condition), the observed pointing direction was more consistent with the prediction of the CtoC hypothesis, although CtoN1 predictions also were partially consistent (CtoC: mean ratio = 0.66; CtoN1: mean ratio = 0.38). The findings for HI-C suggested that participants pointed in manner such that the start location was away from the crossed path, although the effects, while distorted, showed numerically less of a violation than that in Experiment 1. Again, one possibility is that this is due to effect from the true intersection–cross condition (TI-C), an issue we explore shortly.

For the NI-NC condition (also a replication from Experiment 1 in which uncrossed paths matched in terms of visual and idiothetic cues), the observed pointing direction was consistent with the prediction of the NtoN1 hypothesis (mean ratio = 1; Fig 9G and 9H). This replicated the findings in Experiment 1, suggesting near zero spatial distortions and conformity with Euclidean axioms. Again, Rayleigh tests were conducted for each condition. The results showed that the pointing directions in all conditions were not uniformly distributed (*p*s < .001).

**Fig 10:**
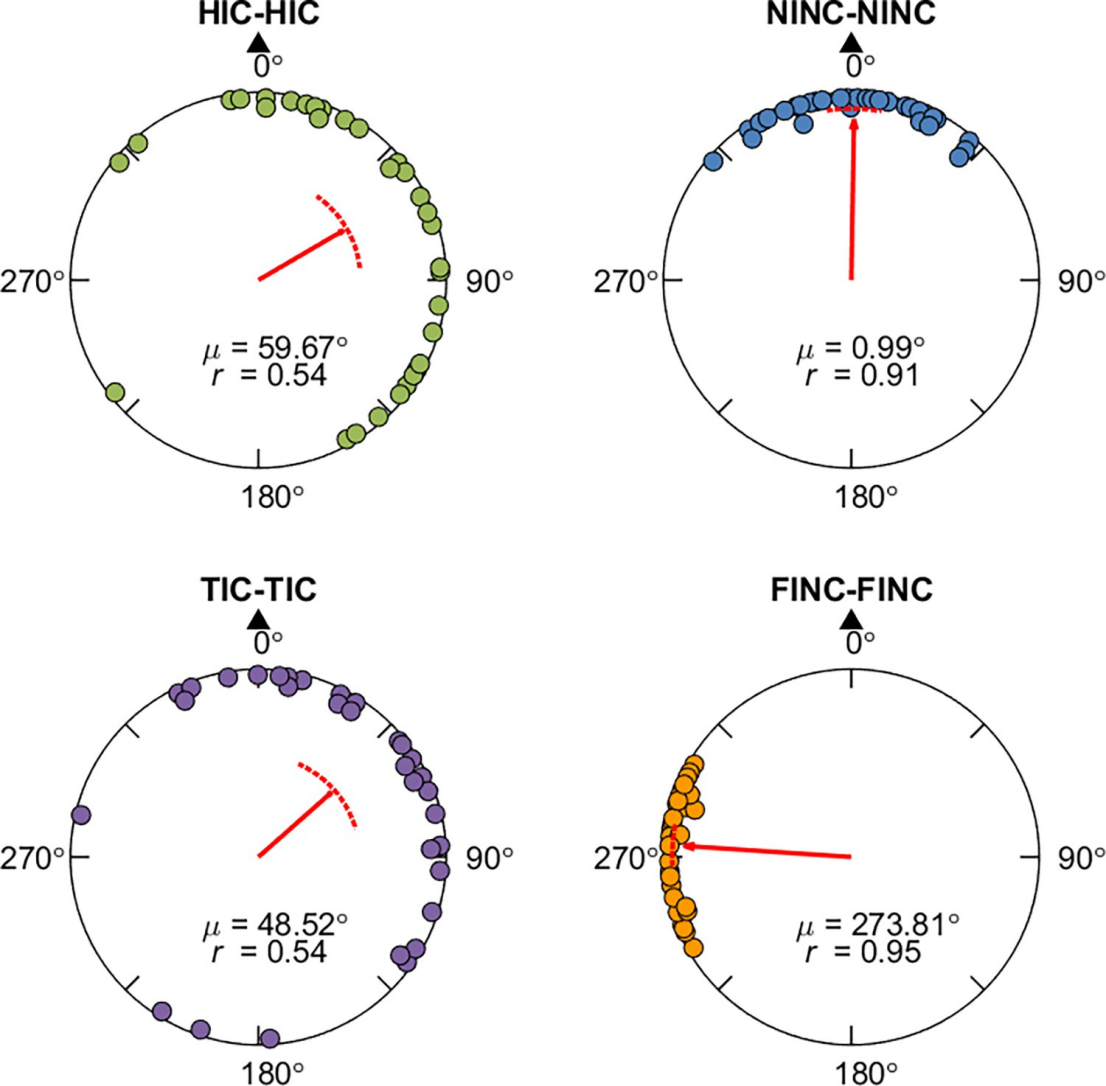
Mean pointing error (AE_G_) across paths for each condition in Experiment 2 with only the trials primed by the same condition. HIC-HIC: HI-C to HI-C condition only. NINC-NINC: NI-NC to NI-NC condition only. TIC-TIC: TI-C to TI-C condition only. FINC-FINC: FI-NC to FI-NC condition only. Abbreviations and legend are the same as in Fig 3.

Returning to the issue of the true intersection—cross (TI-C) and hidden intersection–cross (HI-C) conditions not showing the predicted results, for the TI-C condition, somewhat surprisingly, participants pointed in a manner that was almost indistinguishable from the HI-C condition (HI-C: mean = 39.4°, 95% CI = [21.57°, 57.23°]; TI-C: mean = 50.09°, 95% CI = [32.42°, 67.76°]; Figs 8F and 9B). As the TI-C condition was a condition in which included minimal conflicting cues (i.e., correctly rendered intersection) and the HI-C was a condition in which included conflicting cues (i.e., missing intersections), we investigated why the pointing error was similar. Note that both conditions involved walking crossed paths. Since trials from different conditions were mixed in a random sequence (like in Experiment 1a), we examined the AE_G_ of only the trials primed by the same condition (Fig 10), which would be analogous to the blocked-design experiment (Experiment 1c) we ran as a replication for Experiment 1a. Because of the randomized design, not every participant received equal number of trials priming the same condition. The largest number of trials included for this analysis was eight (in one participant and one condition) whereas the smallest was 0 (in four participants and three conditions). There was a numerical difference between the two conditions in the predicted direction when only including trials primed by the same condition (HIC-HIC: mean = 59.67°, 95% CI = [34.73°, 84.61°]; TIC-TIC: mean = 48.52°, 95% CI = [23.26°, 73.78°]) although an Hotelling’s paired test showed no significant difference, *F*(1, 30) = 0.37, *p* = .697. We return to the issues of the TI-C and HI-C conditions in Experiment 3 using a blocked design.

Finally, we considered the distance at which participants placed the start location. We calculated distance error proportion for each path (error proportion = (correct distance—response distance) / correct distance) and the mean of distance error proportion across paths for each condition (S10 Fig). We found that overall, the participants tended to underestimate the distance to the start (mean = 0.29, SD = 0.20). Using the distance error proportion as a dependent measure, we conducted a 2 (Intersection visibility: Hidden/No intersection, Shown true/false intersection) × 2 (Path type: No cross, Cross) within-subject ANOVA. We found that there was a main effect of Path type, *F*(1, 31) = 14.89, *p* < .001, *η*_*p*_^2^ = .32. The distance error proportion in the cross conditions (TI-C, HI-C: mean = 0.32, SD = 0.20) was significantly greater than that in the no cross conditions (NI-NC, FI-NC: mean = 0.26, SD = 0.20). Neither the main effect of Intersection visibility nor the interaction was significant (both *p*s > .31, *η*_*p*_^2^ < .04). This suggests that simply crossing paths results in some distortions in memory for the distance of start locations, consistent with the greater variability we observed for pointing angular errors in these conditions (Figs 3B, 5B, 8F and 9B).

### 3.5. Discussion

There are two main findings for Experiment 2. First, when experiencing two non-overlapping visual intersections in proximity in the same hallway (FI-NC), participants’ spatial memory distortions appear to violate topology because the start location was remembered as deviated in such a way to possibly accommodate a fifth hallway connecting the two intersections. Consistent with this, the pointing error was significantly different from conditions involving a crossed path and a shown intersection (TI-C) and a no cross condition with no intersection in the same crossed path (NI-NC). The overlapping ratio results suggested the possibility that the pattern of pointing direction involved participants shortening Leg 4 and distorting the two intersections as different but connected ones, although notably, the fit was not perfect (i.e., did not equal 1), suggesting that idiothetic cues still exerted some influence.

Somewhat surprisingly, a second condition that was not in Experiment 1 showing the intersection that was missing in the HI-C condition (true intersection—cross, TI-C) resulted in potential violations of the topological properties of the originally experienced path. It is possible, however, that this may have been partially due to the mixing of four different trial types in the same randomized design. When we isolated TI-C trials that were preceded by only TI-C trials, we found that this condition was closer to zero than when preceded by HI-C trials, although this difference was not statistically significant, possibly due to decreased trials for each condition. In fact, the HI-C condition, when preceded only by HI-C trials, more closely resembled the same results as the HI-C condition in Experiment 1 (further from zero). Notably, the NI-NC condition, which was also presented in Experiment 1, was best fit by a model that assumed no crossing paths, suggesting that this condition was treated according to Euclidean axioms by participants.

## 4. Experiment 3

The purpose of Experiment 3 is to examine the “true intersection—cross” (TI-C) and “false intersection–no cross” (FI-NC) conditions rendered instead in a blocked design. Without the influence of the priming trials, the TI-C condition should show a lower pointing error and should be more likely to fit the prediction of the Cross to Cross (CtoC) hypothesis. Additionally, we predicted replication of FI-NC from Experiment 2. Because the model fits for TI-C were ambiguous in Experiment 2, we significantly increased our sample size in Experiment 3 in the hopes of providing more definitive model fits.

### 4.1. Methods

#### 4.1.1. Participants

We recruited 48 participants (26 female, 22 male) from the University of Arizona undergraduate Psychology program and surrounding Tucson city area whose ages ranged from 18 to 36 years old with a mean age of 22.96. One of them (female) discontinued the experiment in the middle due to cybersickness/motion sickness. Four of the participants (three female and one male) did not complete all the trials due to technical issues with the wireless virtual reality (e.g., HMD disconnection). We obtained complete data from the remaining 43 participants. All participants were compensated at a rate of $20 per hour for their participation. All participants had normal or corrected-to-normal color vision, normal or corrected-to-normal hearing, and reported no history of cardio-vascular problems or motion sickness. Written consent was obtained before the experiment, and the methods were approved by the Institutional Review Board (IRB) at the University of Arizona.

#### 4.1.2. Apparatus

The apparatus was the same as Experiment 1.

#### 4.1.3. Design and procedure

All participants received the TI-C and FI-NC conditions as in Experiment 2 with the following exceptions. First, we used a blocked design instead of a mixed design. Each condition was presented in two blocks of 24 trials in an alternating order. Conditions were counterbalanced across participants. In total, there were 48 trials for each condition. Second, scalp electroencephalography (EEG) recordings were obtained during the behavioral tasks. To focus on the purpose of the current study, here, we only report the behavioral methods and results. For brevity, the results for confidence rating scores were presented in S1 Text and S8 Fig.

### 4.2. Hypotheses

As in Experiment 2, again, we tested four possible models underlying the spatial memory distortions for the true intersection–cross (TI-C) condition: Cross to Cross (CtoC), Cross to No cross (CtoN1, CtoN2, CtoN3; Fig 1, S2 Table). For the false intersection–no cross (FI-NC) condition, we tested three models: No cross to No cross 1 (NtoN1), No cross to Cross (NtoC2), and No cross to Cross (NtoC3; Fig 7, S2 Table).

### 4.3. Data analysis

The exclusion criteria and analysis procedures were the same as in previous experiments (see Section 2.3 and Table 2). Hotelling’s paired tests were applied to compare two conditions of circular pointing errors. All statistical tests were two-tailed with *α* = .05.

### 4.4. Results and discussion

**Fig 11:**
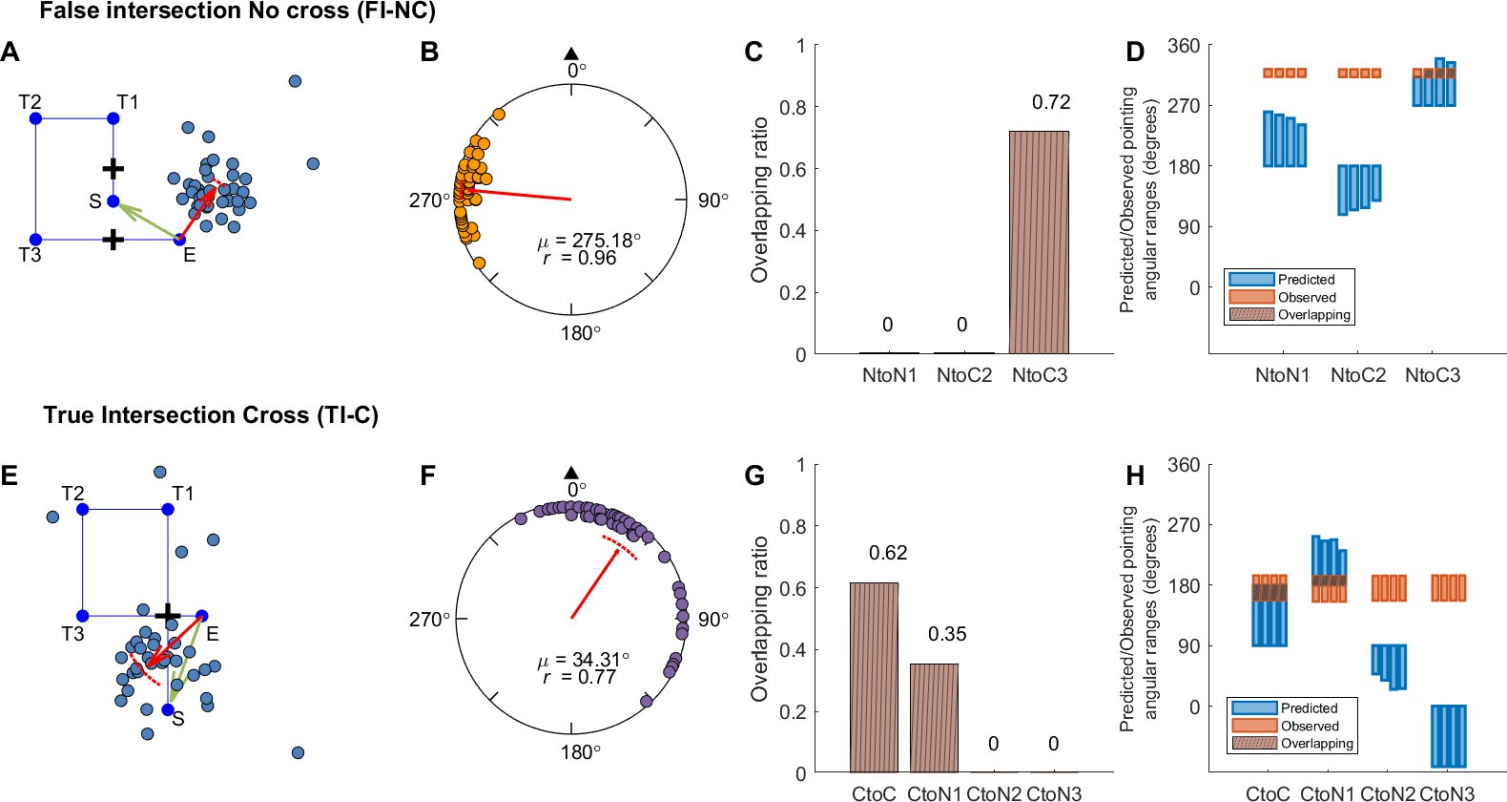
Response pointing directions and hypothesis comparison results in Experiment 3. Panel A, E: Example pointing directions in one path (A: Path 8; E: Path 1). The red arrow indicates the circular mean direction and the distance of the pointing across all participants. Panel B, F: Mean pointing error (AE_G_) across paths for each condition. Panel C, G: Mean overlapping ratio between observed and predicted pointing ranges across paths for each hypothesis (Fig 7, S2 Table). Panel D, H: Predicted or observed pointing angular ranges (in degrees) for individual paths. Abbreviations and legend are the same as in Fig 3.

The response pointing directions and locations for each path were shown in Fig 11A and 11E) and S11 Fig. Rayleigh tests showed that the pointing directions in both conditions were not uniformly distributed (*p*s < .001). In the false intersection no cross (FI-NC) condition, just as in Experiment 2, the participants pointed at 275.18° counterclockwise to the correct direction (95% CI = [270.04°, 280.31°]; Fig 11B), significantly deviated from 0°/360°. In the true intersection—cross (TI-C) condition, the participants pointed at 34.31° clockwise to the correct direction (95% CI = [20.73°, 47.90°]; Fig 11F), which was a numerically smaller pointing error compared to TI-C in Experiment 2 (Figs 8F and 10 “TIC-TIC”; although the Mardia-Watson-Wheeler test did not show a significant difference, *W* = 2.48, *p* = .290). An Hotelling’s paired test showed that there was again a significant difference in AE_G_ between the FI-NC and TI-C conditions (*F*(1, 41) = 717.95, *p* < .001).

As in previous experiments, we conducted a Bayesian projected normal regression model (pointing angle ~ hypothesis+ subject) for each condition and examined which of our hypotheses (Fig 7, S2 Table) fit the observed pointing directions in each condition. The linear coefficients for Component I and II are shown in S7 Table. The random effect variances are shown in S4 Table. We compared the observed pointing directions to the hypothesized angular ranges and found that the observed pointing direction in the TI-C condition was more consistent with the predictions of the Cross to Cross (CtoC) hypothesis (mean ratio = 0.62; Fig 11G and 11H). For the FI-NC condition, the observed pointing direction was consistent with the predictions of the No cross to Cross (NtoC3) hypothesis (mean ratio = 0.72; Fig 11C and 11D). These results replicated the findings for FI-NC from Experiment 2 and suggest that, without hidden intersection—cross (HI-C) condition present in a randomized design, the pointing responses in TI-C condition more closely resembled geometrically accurate pointing.

**Fig 12:**
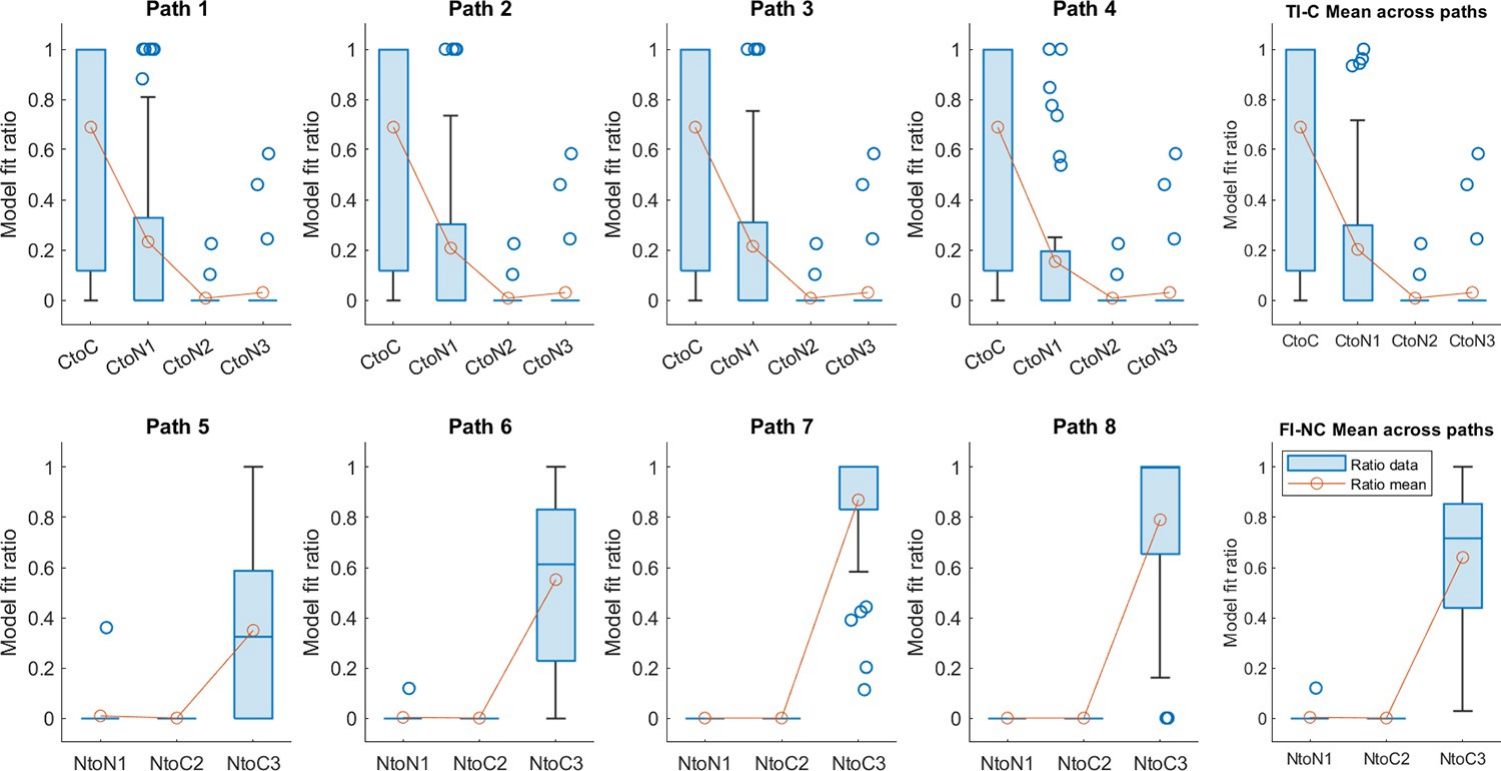
Distribution of model fit ratios for individual participants in Experiment 3. Orange circles show the mean ratios of each hypothesis. Box boundaries show interquartile ranges. Lines inside box show medians. Lower and upper error lines show nonoutlier minimum and maximum values, respectively.

Since Experiment 3 included more trials for each condition (48 trials) than previous experiments (16 trials), we further conducted a Bayesian projected normal regression model (pointing angle ~ hypothesis + path number) on individual trials and examined which of our hypotheses (Fig 7, S2 Table) fit the observed pointing directions for each participant. To include more data points in this analysis, we did not exclude any trials. The mean fit ratio was summarized in Fig 12. Most participants showed the same model fit results as the group-level analysis, showing that the observed pointing directions in TI-C condition were more consistent with the Cross to Cross (CtoC) hypothesis. In contrast, the pointing directions in FI-NC condition were again more consistent with the No cross to Cross (NtoC3) hypothesis. For the TI-C condition, a few participants’ pointing responses (see the outlier dots in column “CtoN1” in Fig 12) were more consistent with the Cross to No cross 1 (CtoN1) hypothesis than the CtoC hypothesis, which suggests significant spatial memory distortions. Specifically, our path shape models suggested that these participants still underestimated L1 even when there was minimal conflicting information (TI-C condition) and no influence from priming trials in another condition (see also Experiment 1b). We return to this issue in the general discussion.

We also examined the pointing distance error by calculating a distance error proportion (S10 Fig). Three participants were excluded from this analysis because their distance error proportions exceeded 2 × SD limit (mean = -3.51, -12.95, -7.19, respectively). Overall, the distance error proportion was low (TI-C: mean = -0.01, median = 0.15, SD = 0.62; FI-NC: mean = -0.05, median = 0.15, SD = 0.71) despite substantial individual differences. Both conditions were not significantly different form 0 (both *p*s > .67, Cohen’s *d*s < 0.10). A paired t test showed that there was no significant difference in the distance error proportion between the two conditions, *t*(39) = 0.71, *p* = .480, Cohen’s *d* = 0.16.

## 5. Experiment 4

The purpose of Experiment 4 is to examine if the experimental conditions rendered in desktop VR (with only visual input) would lead to differences in pointing responses from those with both visual and idiothetic cues. Without idiothetic input from walking, all conditions should show a higher pointing error and variability than previous experiments. But the no intersection—no cross (NI-NC) condition, as the no-conflict, control condition with no crossings, should show more consistent and less variable responses than other conditions.

### 5.1. Methods

#### 5.1.1. Participants

We recruited 28 participants (18 female, 9 male) from the University of Arizona undergraduate Psychology program whose ages ranged from 18 to 22 years old with a mean age of 19.14. Three additional participants (1 female, 2 male) chose to quit the study before completing all trials. All participants were compensated with psychology course credits for their participation. All participants had normal or corrected-to-normal color vision, normal or corrected-to-normal hearing, and reported no history of cardio-vascular problems or motion sickness. Written consent was obtained before the experiment, and the methods were approved by the Institutional Review Board (IRB) at the University of Arizona.

#### 5.1.2. Apparatus

A computer with a refresh rate of 60 Hz was used to present the stimuli. The screen resolution was 1920 × 1080 pixels. An Xbox controller was used for virtual navigation and making responses.

#### 5.1.3. Design and procedure

**Fig 13:**
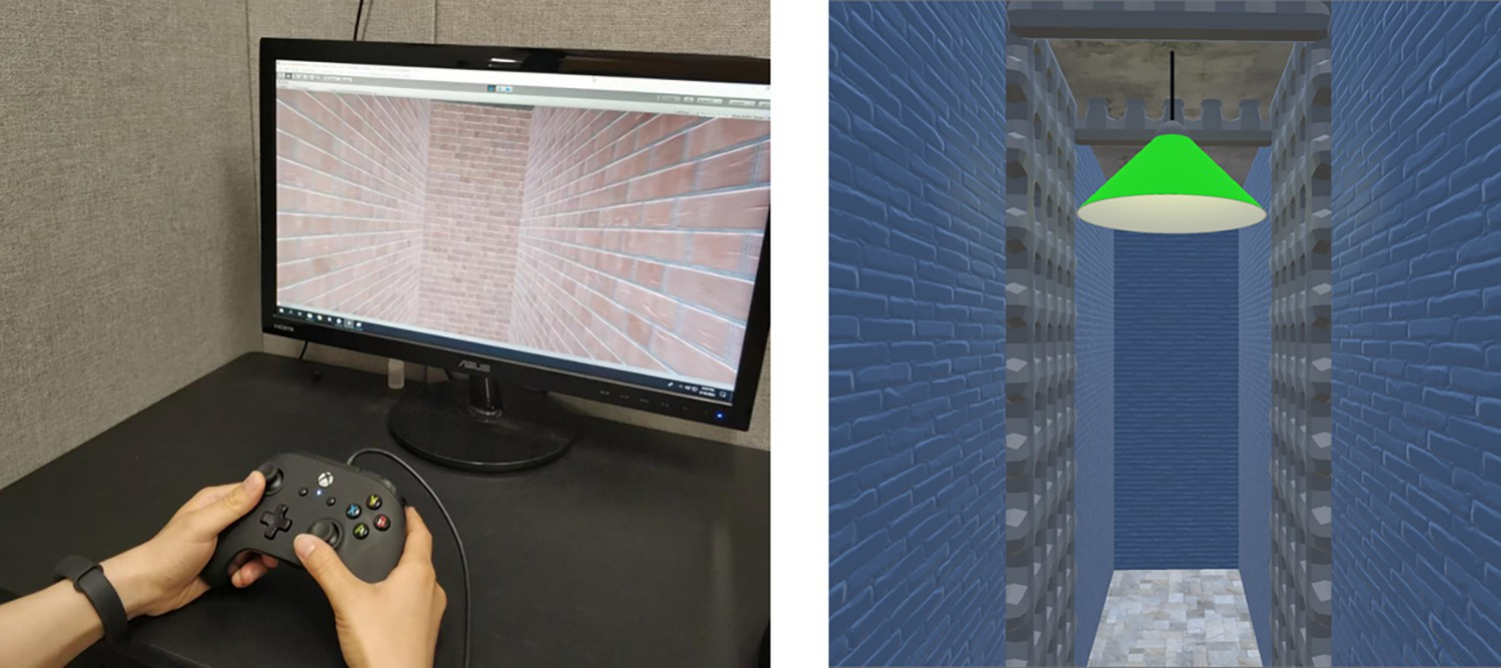
Experimental setup with the Xbox and example views of the intersection in Experiment 4.

All participants received the four conditions in Experiment 2: HI-C, NI-NC, TI-C and FI-NC with the following changes. First, a hanging lamp from the ceiling was added to the intersections in all hallways in the TI-C and FI-NC conditions to improve the visual salience of the intersections (Fig 13). Second, because Experiments 2&3 indicate that the true intersection–cross condition was likely influenced by the hidden intersection—cross (HI-C) condition, we used a blocked design instead of a mixed design. Each condition was presented in a block of 24 trials. Conditions were counterbalanced across participants in a Latin-square order such that a no-cross condition was always followed by a cross condition and vice versa. There were eight testing orders in total:

NINC → TIC → FINC → HIC
TIC → FINC → HIC → NINC
FINC → HIC → NINC → TIC
HIC → NINC → TIC → FINC
NINC → HIC → FINC → TIC
HIC → FINC → TIC → NINC
FINC → TIC → NINC → HIC
TIC → NINC → HIC → FINC

Third, in the disorientation phase, the participants did the verbal counting task as before without physical rotations. After that, the hallway was shown to them and they were placed at the start of the hallway. Fourth, they navigated through the hallway using two joysticks on the Xbox controller (one for forward translation, the other for turning; Fig 13). When participants finished virtual navigation and reached the end of the hallway, they used a joystick on the Xbox controller to indicate which direction the start location was. Only the pointing direction was recorded on each trial. Fifth, in the confidence rating phase, they used the other joystick to adjust the size of the rectangle and indicate their confidence about their pointing. For brevity, the results for confidence rating scores were presented in S1 Text and S8 Fig.

### 5.2. Data analysis

Data from three participants (all female) were excluded from analysis because they failed to follow the instructions. In addition, 3.5% of trials in the remaining 25 participants were missing due to the participants’ incorrect use of the Xbox controller or the experimenter’s error; these trials were therefore excluded from analysis. We did not perform the circular error exclusion procedure as in previous experiments because the conditions showed significant variability and, at least for two conditions, uniform pointing (see Section 5.3). All statistical tests were two-tailed with *α* = .05.

### 5.3. Results and discussion

**Fig 14:**
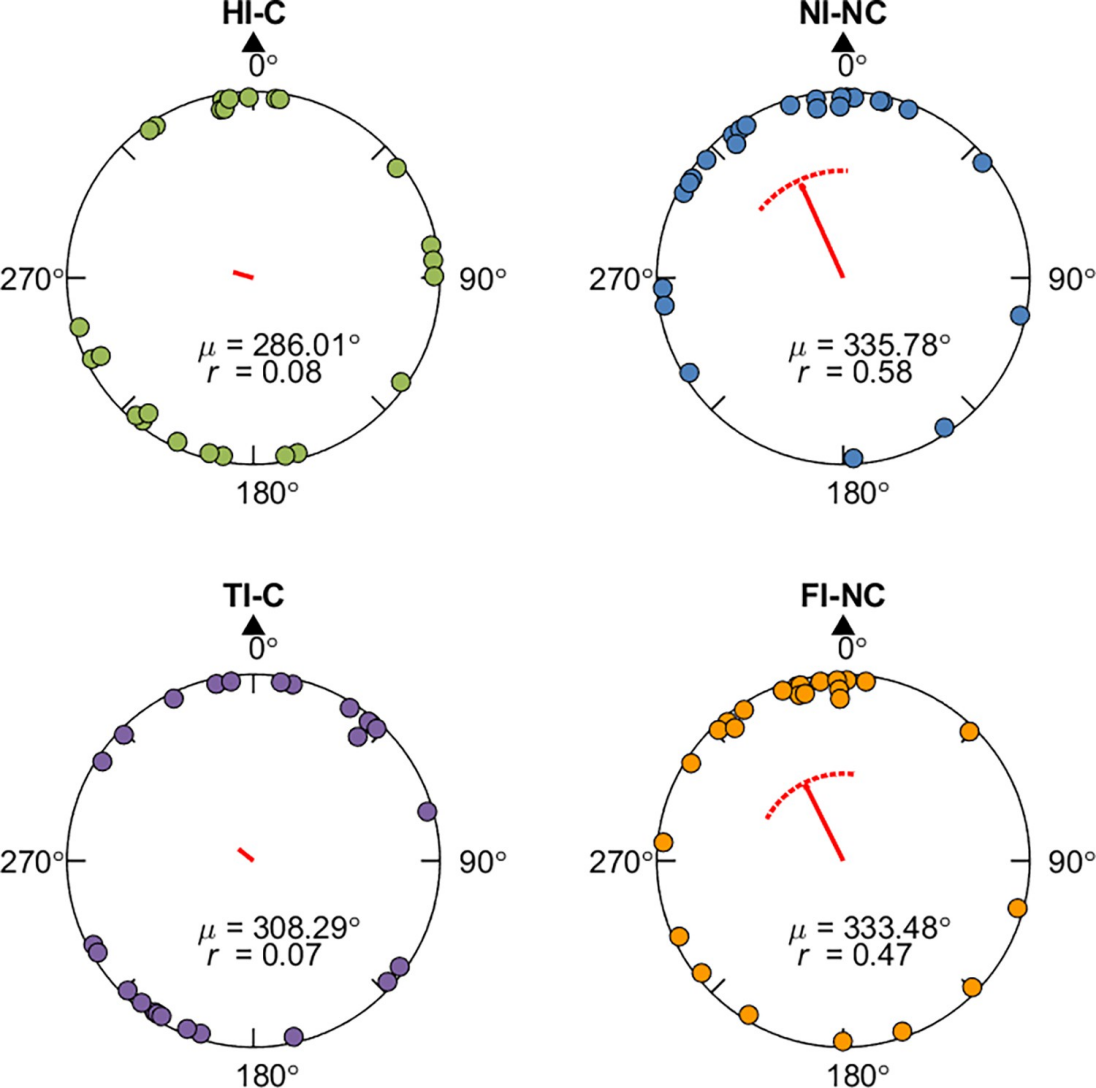
Mean pointing error (AE_G_) across paths for each condition in Experiment 4. Abbreviations and legend are the same as in Fig 3.

The response pointing directions for each path were shown in Fig 14 and S12 Fig. Rayleigh tests showed that the pointing directions in the two uncrossed conditions (NI-NC and FI-NC) were not uniformly distributed (*p*s < .004). Both conditions, however, showed numerically larger variability than the same conditions in previous experiments (Table 2; NI-NC: *r* = .58, kappa = 1.39; FI-NC: *r* = .47, kappa = 1.01). The mean pointing direction in both conditions were close to the correct directions; the pointing errors (AE_G_) were close to 0°/360° (NI-NC: mean = 335.78°, 95% CI = [301.52°, 5.43°]; FI-NC: mean = 335.78°, 95% CI = [310.879°, 0.69°]). An Hotelling’s paired test on AE_G_ showed that there was no significant difference between FI-NC and NI-NC conditions (*F*(1, 23) = 0.77, *p* = .473). Mardia-Watson-Wheeler tests showed that for both conditions, there was a significant difference between the pointing patterns in the current experiment and Experiment 2, respectively (NI-NC: *W* = 7.12, *p* = .028; FI-NC: *W* = 38.36, *p* < .001). There was also a significant difference between the pointing patterns in the current experiment and Experiment 3 for the FI-NC condition, *W* = 37.37, *p* < .001.

By contrast, the pointing directions in the two crossed conditions (HI-C and TI-C) were uniformly distributed (*p*s > .84). Both conditions showed numerically much larger variabilities than the same conditions in previous experiments (Table 2; HI-C: *r* = .08, kappa = 0.18; TI-C: *r* = .07, kappa = 0.16). Mardia-Watson-Wheeler tests showed that for both pointing patterns in both conditions, there was a significant difference between the current experiment and Experiment 2, respectively (HI-C: *W* = 16.26, *p* < .001; TI-C: *W* = 10.13, *p* = .003). There was also a significant difference for the pointing patterns between the current experiment and Experiment 3 for the TI-C condition, *W* = 12.37, *p* = .002.

In Experiment 4, when removing idiothetic cues from navigation and leaving only visual cues, all conditions showed different pointing patterns from Experiments 1–3. Participants showed random pointing responses in the two cross conditions (HI-C and TI-C) yet pointed accurately (near zero error) yet more variably in the two no-cross conditions (FI-NC and NI-NC). In particular, the responses in the false intersection—no cross (FI-NC) condition, which showed large memory distortions in Experiments 2&3, were similar to the no intersection—no cross (NI-NC) condition, which showed little to no distortions throughout all experiments. Such results indicate that although the visual cues in the NI-NC and FI-NC conditions still provided spatial information for traveled length in the hallway, visual cues alone not appear to produce the memory distortions found in Experiments 1–3. Such memory distortions were more likely to be caused by the competition between visual and idiothetic cues rather than visual cues alone.

## 6. General discussion

While there is little doubt that our memories for spatial environments are distorted from the actual experienced environment, the nature of these distortions remains unclear. In particular, whether and how such distortions might involve topological changes in the geometry of the space we experienced remains largely untested. Here, we report that, even in simple geometric patterns like hallways, such topological distortions (e.g., creating an intersection where there is not one) readily occur. We summarize the results of all experiments in Table 3. In Experiment 1, we found that when participants did not encounter an expected intersection when walking a crossed path (a visual intersection in a physically crossed path; hidden intersection—cross, HI-C), they pointed to a start location suggesting a memory distortion of an uncrossed path. Specifically, they pointed as if one segment of the path was distorted in some form (e.g., shortened, as indicated by best fit CtoN1 hypothesis). Yet, when participants were guided to walk the same path with poles instead of the hallways (pole-guided cross, PG-C), relying primarily on idiothetic cues, they showed significantly reduced pointing error and model fitting suggested preservation of a crossed path. In Experiment 2, more strikingly, when two false intersections were shown to the participants in an uncrossed path (false intersection no cross, FI-NC), our results suggested significant distortions in spatial memory involving remembering a crossed path via a fifth (unexperienced) hallway. By contrast, when the uncrossed path was presented without the false intersections (no conflict; no intersection—no cross, NI-NC), participants pointed accurately with few errors (near 0° pointing error), suggesting minimal distortions and largely consistent with the physically experienced geometry. Experiment 3 replicated the findings in the FI-NC condition with a blocked design. Experiment 4 demonstrated that idiothetic cues contributed to the findings related to FI-NC and HI-C. It is noteworthy that the strongest and most consistent distortions we observed were for FI-NC in Experiments 2 and 3 involving full rendering of idiothetic cues. Notably, FI-NC involved rendering two false intersections, rather than for HI-C, which involved removing an expected intersection. At least some of this difference in effect size and consistency may relate to cue salience (i.e., absence vs. presence of an intersection), an issue often noted in the attention and memory literature [48].

**Table 3:** Summary of results in all experiments.

Experiment	Condition	Best fit hypothesis
1a (hallway mixed design)	Hidden intersection Cross (HI-C)	Crossed paths to uncrossed paths (CtoN1)
	No intersection No cross (NI-NC)	Uncrossed paths remain uncrossed (NtoN1)
1b (pole-guided)	Pole-guided cross (PG-C)	Crossed paths remain crossed (CtoC)
	Pole-guided No cross (PG-NC)	Uncrossed paths remain uncrossed (NtoN1)
1c (hallway blocked design)	Hidden intersection Cross (HI-C)	Crossed paths to uncrossed paths (CtoN1)
	No intersection No cross (NI-NC)	Uncrossed paths remain uncrossed (NtoN1)
2 (hallway mixed design)	Hidden intersection Cross (HI-C)	Crossed paths remain crossed (CtoC)
	True intersection Cross (TI-C)	Crossed to uncrossed paths (CtoN1)
	No intersection No cross (NI-NC)	Uncrossed paths remain uncrossed (NtoN1)
	False intersection No cross (FI-NC)	Uncrossed paths to crossed paths (NtoC3)
3 (hallway blocked design)	True intersection Cross (TI-C)	Ambiguous fit (CtoC and CtoN1)
	False intersection No cross (FI-NC)	Uncrossed paths to crossed paths (NtoC3)
4 (desktop hallway blocked design)	Same as Exp 2	N/A

In our experiments, partially distorted spatial memories were the norm, although these distortions differed in nature and likely arose from different underlying mechanisms. Even for hallways in which visual and idiothetic cues matched, which should have provided consistent information for encoding the physical start location (e.g., the true intersection condition in Experiments 2 and 3, TI-C), we still observed distortions in memory, although these were somewhat less pronounced than the conflicting conditions. Specifically, the true intersection condition involved rendering a single intersection (in physical space) at its correct location in terms of the physically crossed path. Surprisingly, though, in Experiment 2, we found a slightly better fit for the cross-to-uncross model (involving deleting an intersection) than the cross-to-cross model (in which the topology is retained). Notably, however, these distortions were less pronounced in Experiment 3 and more consistent with maintaining the physical geometry (i.e., fit to Cross-to-Cross model) in which the true intersection occurred in consecutively in a blocked design and we had a larger sample size, better positioning us to observe smaller effects-sizes. While we did observe some distortions in the pole-guided condition and non-intersecting non-crossed (NI-NC) paths in Experiment 1, these distortions were again less pronounced and fit better with models that assumed preservation of crossed and uncrossed paths. These findings suggest that distortions in spatial memory for the shapes of walked paths are common, although for the conditions involving matching between visual and idiothetic cues, such distortions often comported well with the physically experienced (Euclidean) geometry.

At least some of the distortions we observed, particularly in Experiment 2, were likely the result of influence from the identical physically walked condition, hidden intersection-cross (HI-C) influencing the true intersection–cross condition (TI-C). This was evidenced by a comparison of true intersection-cross (TI-C) trials that were not preceded by HI-C in Experiment 2 and the results from the blocked design in Experiment 3, which included only crossed (TI-C) and uncrossed false intersection–no cross (FI-NC) physically walked paths, suggesting a less distorted memory for the first path and better fit to the “crossed” model. Such “history effects,” in which memories for past trials influence current trials, may involve a regression to the mean phenomenon in which two similar conditions (in this, HI-C and TI-C) interfere in some form with each other when experienced in proximity. Indeed, multiple path integration studies have observed such regression to the mean effects, which likely relates to a tendency to store a running average of the shapes of paths walked across trials [1,27,49]. The findings for true intersection–cross (TI-C) suggest the possibility, to be tested in future studies, that geometric distortions in spatial memory can occur even when visual and idiothetic cues match due to influences from previous (mismatching) trials.

The distortions we observed in the other conditions in which visual and idiothetic cues matched (no intersection–no cross, NI-NC), true intersection—cross condition (TI-C) in Experiment 3, or the pole-guided conditions involving primarily idiothetic cues, were best fit by models assuming Euclidean axioms and no topological changes. Our modeling results suggested that NI-NC (Experiment 1) and the pole-guided conditions involved some possible underestimation of the first leg. Such a finding has also been reported in studies in which participants walk two legs of a triangle and attempt to walk back to the start, termed the triangle completion task. One finding from these studies, particularly for larger triangles, is that participants underestimate the distance they need to walk and overestimate the direction they need to turn [1,27,28,50]. Modeling results suggest that one possible explanation for the underestimation of distance / overestimation of turning angle is under-encoding of the first leg of the triangle [1]. Thus, even under situations in which participants walk a regular shape triangle with idiothetic cues only, they show some evidence of memory distortions similar to what we have shown here in “geometrically consistent” conditions. While the theoretical reasons for this remain unclear, it is possible that these may relate to working memory errors [1]. Importantly, though, it is likely that the mechanisms influencing the memory distortions for NI-NC, TI-C, and the pole-guided conditions likely have different origins from the memory distortions observed for the mismatching conditions FI-NC and HI-C.

While our results overall converged across our multiple experiments to show that participants distorted their memories for paths they walked when visual and idiothetic cues mismatched, another issue is that there were some differences when we tested the same conditions across multiple experiments (i.e., HI-C). For example, in Experiment 1a, we found that participants significantly altered their pointing locations in the HI-C condition (83.59° deviated from the correct geometric response when the hallway was missing the expected intersection), fitting CtoN1 hypothesis in which participants pointed as if there were no intersection (Table 3), yet in the blocked-design version (Experiment 1c) and the HI-C condition in Experiment 2 (both as a replication to HI-C condition in Experiment 1a), our modeling results suggested the possibility that the under-encoding of Leg 1 / over-encoding of Leg 3 was less dramatic (48.44° and 39.4° deviated from 0°, respectively). Notably, the pointing patterns did not differ from each other for HI-C across the three replications (according to their confidence intervals; Experiment 1a HI-C: [41.51°, 125.68°]; Experiment 1c HI-C: [15.24°, 81.64°]; Experiment 2 HI-C: [21.57°, 57.23°]). While it is difficult to speculate on the reasons for a numeric but non-significant difference, one issue to note is that replication is probabilistic rather than deterministic and that the effect sizes when testing the same condition multiple times may either overestimate or underestimate the original effect size [51,52]. Given that our replications were contained within follow-up experiments involving novel manipulations, it is also possible, as mentioned before, that the conditions that the participant had experienced previously had some influence on their pointing patterns in the current trial (i.e., that TI-C influenced HI-C and vice versa in Experiment 2).

Models of spatial navigation which allow for local distortions of path configurations and violations of Euclidean postulates, such as labeled graph theory, appear to provide a reasonable account for our findings [16,21]. For example, labeled graph theory [23] posits that participants encode the intersections as nodes and paths as labeled “lengths.” As such, when a participant unexpectedly encounters an intersection (node) in the false intersection–no cross (FI-NC) condition, they could compress two nodes (the unexpected intersection and the end point), in the process violating the rule that the two nodes must maintain a distance greater than zero. This would allow for a distortion in which the start location is remembered as significantly deviated from its location in physical space (Fig 1), something that would not be possible according to Euclidean (and metric) axioms that their distance must remain greater than zero. Similarly, when walking a path that crosses on itself but not seeing an expected intersection (hidden intersection—cross, HI-C condition), participants remembering a crossed path as an uncrossed path would possibly be consistent with “deleting” a node (the walked crossing point), creating a positive distance from an otherwise zero distance (the deleted node). Because participants pointed at the end of the path, the “moment” the distortions occurred in the mismatching conditions is not directly tested, and thus, it is not clear whether the effects we observed occurred at encoding, retrieval, or both [26]. Our findings from Experiment 4 do suggest that both visual and idiothetic cues are likely necessary for the effects we observed in the mismatching conditions, suggesting that competition between idiothetic and visual cues at encoding likely contributed to our effects.

Our findings, particularly for the mismatching conditions, appear inconsistent with models that operate under Euclidean axioms [39–41]. According to these models, if one path is remembered as distorted, the other parallel paths would also be encoded as distorted in a similar fashion, according to the properties of parallel and equal length lines. Perhaps more problematic, according to metric postulates, the distance between two points must remain positive and the distance between a point and itself must remain zero, a property that was violated, particularly in the FI-NC condition. Another component of cognitive map theory is that path integration information derived from physically ambulating (i.e., idiothetic cues) is primary to spatial representation, with visual landmarks integrated through a gradual learning process [40,53]. It would seem that, in the face of conflict between idiothetic and visual cues, participants should choose the information provided by idiothetic cues and ignore the visual cues.

Alternatively, participants could choose a representation entirely consistent with the visual cues, which, although inconsistent with their physically experienced geometry, could also be considered consistent with Euclidean geometry. According to this perspective, participants could be choosing between two “plausible” cognitive maps, one based on path integration information (e.g., no cross) and one based on the visually rendered “false” intersections (e.g., crossed auxiliary hallway). The real-world analogue of this might be reorientation: if we miss an expected intersection when driving, we simply mentally reposition ourselves based on our current visual information. Notably, though, participants in all of the experiments did not appear to engage in this simpler binary “map” selection process: because none of the model fits were perfect (i.e., 1), all of our results suggest that participants chose a model that partially reconciled the conflicting idiothetic and allothetic cues, thereby creating distortions of both their physically experienced geometry and what might otherwise be plausible based on visual information alone. Also, given that almost all conditions, even the walked paths that involved no cue conflict, showed memory distortions, our findings are more consistent with labeled graph theory because it allows greater flexibility in representation selection / formation. How this relates to real-world situation is less clear but might involve “splitting the difference” between where our we think we are based on our bearing and our best guest based on visual cues.

Is it possible that the distortions we observed resulted from a preference for visual cues (rather than a domination) over idiothetic cues, and in this way, still showed a “bias” toward a visually plausible cognitive map? In Experiment 4, we tested this idea by providing participants with the same visual input (true intersection-cross [TI-C] and false intersection-no cross [FI-NC]; additionally, we added a more salient visual landmark, i.e., the hanging lamp at the intersection) but impoverished idiothetic cues in desktop VR. In this case, the only condition that showed consistency with its corresponding condition in immersive (walking) VR was the no-cross no-intersection (NI-NC) condition. For hidden intersection—cross (HI-C) and true intersection—cross (TI-C) conditions, pointing accuracy was near random. For the false intersection—no cross (FI-NC) condition in Experiment 4, participants pointed in a manner consistent with the experienced path (in other words, little to no distortion), contrary to our findings in Experiments 2&3. Given the differences between our desktop and immersive (walking) conditions, and low distortions in the pole-guided (idiothetic) condition, our findings suggest that the distortions we observed likely arise from competition between idiothetic and visual cues [12,33,34]. This is also notable in terms of our findings for the immersive (walking) VR conditions in Experiments 1 and 2. Even for FI-NC and HI-C, which involved mismatching visual and idiothetic cues, the distortions were not complete and only partially fit by models assuming that the visual cues shortened the first (HI-C) or last path (FI-NC) paths. These findings suggest that even in the face of mismatching information, path integration computations exerted some influence on participants knowledge about the shape of the paths they walked. Importantly, these findings also suggest that the distortions we observed did not simply involve selecting one “map” over another but rather an active process of altering topological aspects of paths in memory to reconcile competing idiothetic and allothetic cues.

## Supporting information

S1 Fig Response pointing directions in Experiment 1a. HI-C: hidden intersection cross condition. NI-NC: no intersection no cross condition. Each dot indicates the pointing direction for one participant. The red arrow indicates the circular mean direction (μ) of the pointing directions across all participants. r is the mean resultant length of all pointing angles. The arc above the mean direction indicates the 95% confidence interval of the mean direction.
(TIF)

S2 Fig Response pointing directions in Experiment 1b. PG-C: Pole-guided Cross condition. PG-NC: Pole-guided No cross condition. Legend is the same as in S1 Fig.
(TIF)

S3 Fig Response pointing directions in Experiment 1c. Abbreviations and legend are the same as in S1 Fig.
(TIF)

S4 Fig An example of outlier detection (Experiment 1a, HI-C). Left: Each dot indicates the circular mean across trials with the same path shape for each participant. The green and blue lines indicate the mean direction of the sample before and after excluding outliers, respectively. Red dots indicate the outliers in this sample. Right: Observed pointing angles as a function of c-statistics. The red vertical line indicates the cut-off value of c-statistic in this sample. The dots at the right side of the red line indicate the outliers in this sample and correspond to the red dots on the left panel.
(TIF)

S5 Fig Response pointing directions and hypothesis comparison results without removing outliers for groups with more than 5% outliers. Panel A, E, I, M: Example pointing directions in one path. A: Path 3. E: Path 5. I: Path 7. M: Path 4. Panel B, F, J, N: Mean pointing error across paths (AE_G_). Panel C, G, K, O: Mean overlapping ratio between observed and predicted pointing ranges across paths for each hypothesis (see Figs 1 and 7 and S2 Table). Panel D, H, L, P: Predicted or observed pointing angular ranges (in degrees) for individual paths. Abbreviations and legend are the same as in Fig 3.
(TIF)

S6 Fig Absolute angular pointing errors (AE_G_). Error bars show the standard errors of the mean in each condition.
(TIF)

S7 Fig Response pointing positions in Experiment 2. TI-C: True Intersection Cross condition. FI-NC: False Intersection No cross condition. The red arrow indicates the circular mean direction (μ) and the distance of the pointing across all participants. Other abbreviations and legend are the same as in S1 Fig.
(TIF)

S8 Fig Confidence rating in Experiments 2–4. Error bars show the standard errors of the mean in each condition.
(TIF)

S9 Fig Examples of the predicted pointing directions according to the No cross to Cross (NtoC2) hypothesis in Experiments 2 and 3. E’_far_: the estimated position of the end if the intersection is estimated to overlap with T1. Other abbreviations and legend are the same as in Fig 7.
(TIF)

S10 Fig Pointing distance error proportion in Experiments 2 and 3. Error bars show the standard errors of the mean in each condition. The dots show the data of individual participants.
(TIF)

S11 Fig Response pointing positions in Experiment 3. Abbreviations and legend are the same as in S7 Fig.
(TIF)

S12 Fig Response pointing directions in Experiment 4. Abbreviations and legend are the same as in S7 Fig.
(TIF)

S1 Table Path dimensions (in meters) in Experiment 1. (DOCX)

S2 Table Predicted angular ranges and mean (in degrees). (DOCX)

S3 Table Circular regression results in Experiment 1. Posterior modes and 95% highest posterior density (HPD) lower (LB) and upper bounds (UB) for the regression coefficients of the angular error for each group and condition.
(DOCX)

S4 Table Random effects in circular regression. Posterior mode and 95% highest posterior density (HPD) lower (LB) and upper bounds (UB) of the random effect variances in regression for each group and condition.
(DOCX)

S5 Table Path dimensions (in meters) in Experiments 2–4. (DOCX)

S6 Table Circular regression results in Experiment 2. Posterior modes and 95% highest posterior density (HPD) lower (LB) and upper bounds (UB) for the regression coefficients of the angular error for each group and condition.
(DOCX)

S7 Table Circular regression results in Experiment 3. Posterior modes and 95% highest posterior density (HPD) lower (LB) and upper bounds (UB) for the regression coefficients of the angular error for each group and condition.
(DOCX)

S1 Text Supplementary results. (DOCX)

S1 File R script for calculating the cutoff value of c-statistics. (R)
