# Correction: Spatial memory distortions for the shapes of walked paths occur in violation of physically experienced geometry

**DOI:** 10.1371/journal.pone.0361361

**Published:** 2026-10-26

**Authors:** Yu K. Du, Andrew S. McAvan, Jingyi Zheng, Arne D. Ekstrom, the PLOS One Editors

Following publication of the article [1], the authors discovered errors in [1] due to a coding error in an analysis script. The errors affect the results of the false intersection-no cross (FI-NC) condition in Experiments 2 and 3 and do not affect the other conditions and groups.

The updated results following correction of the coding error indicate overall patterns that are qualitatively consistent with those reported originally, specifically that the false visual intersection results in a difference from both the true intersection, cross (TI-C) and no intersection, no cross (NI-NC) conditions. The group modeling results for FI-NC are affected, although there is evidence for individual- and path-level variability, which is consistent with the presence of non-Euclidean distortions reported in the original article. This Correction addresses the following specific errors:

In the 3.4 Results subsection of the 3. Experiment 2 section, there is an error in the last sentence of the first paragraph. The correct sentence is: In the FI-NC condition, which was a critical test of our hypothesis regarding memory reconstructions of walked paths based on false intersections, we found that participants pointed at 351.07° counterclockwise left to the correct direction (95% CI = [345.75°, 356.38°]; Fig 8B), significantly deviated from 0°/360°.

In the 3.4 Results subsection of the 3. Experiment 2 section, there are errors in the third and fifth sentences of the second paragraph. The correct paragraph is: To determine whether the manipulation of the false intersections affected participants’ memory for the paths they walked in the FI-NC condition, we conducted Hotelling’s paired tests for AE_G_ (Figs 8B, 8F, 9B and 9F). We first compared the FI-NC and TI-C conditions because in both conditions, participants saw and walked through the “intersection” twice. There was a significant difference in pointing for the FI-NC compared to the TI-C conditions (*F*(1, 30) = 29.24, *p* < .001), suggesting that participants treated these conditions differently. We also compared with NI-NC condition, in which no intersections were shown but involved the same uncrossed path and was identical to the NI-NC condition in Experiment 1. We also found a significant difference between FI-NC and NI-NC (*F*(1, 30) = 11.66, *p* < .001; NI-NC: mean = 0.93°, 95% CI = [354.30°, 7.56°]; Fig 9F). These findings further support the idea that rendering the “false” intersections affected participants memories for the paths they walked.

In the 3.4 Results subsection of the 3. Experiment 2 section, there is an error in the fourth sentence of the third paragraph. The correct sentence is: For the false intersection no cross (FI-NC) condition, the observed pointing directions were consistent with the prediction of the No cross to Cross (NtoC3) hypothesis as indicated by the overlapping ratio (mean ratio = 0.88; Fig 8C and 8D).

There is an error in Fig 10. The AE_G_ (Mean pointing error) results in the FINC-FINC panel are incorrect. Please see the correct Fig 10 included with this Correction.

In the 4.4 Results and discussion subsection of the 4. Experiment 3 section, there is an error in the second sentence of the first paragraph. The correct sentence is: In the false intersection no cross (FI-NC) condition, just as in Experiment 2, the participants pointed at 352.67° counterclockwise to the correct direction (95% CI = [347.48°, 357.87°]; Fig 11B), significantly deviated from 0°/360°.

In the 4.4 Results and discussion subsection of the 4. Experiment 3 section, there is an error in the last sentence of the first paragraph. The correct sentence is: An Hotelling’s paired test showed that there was again a significant difference in AE_G_ between the FI-NC and TI-C conditions (*F*(1, 30) = 25.20, *p* < .001).

In the 4.4 Results and discussion subsection of the 4. Experiment 3 section, there is an error in the fifth sentence of the second paragraph. The correct sentence is: For the FI-NC condition, the observed pointing direction was consistent with the predictions of the No cross to Cross (NtoC3) hypothesis (mean ratio = 0.89; Fig 11C and 11D).

In the 4.4 Results and discussion subsection of the 4. Experiment 3 section, there are errors in the third paragraph. The correct paragraph is: Since Experiment 3 included more trials for each condition (48 trials) than previous experiments (16 trials), we further conducted a Bayesian projected normal regression model (pointing angle ~ hypothesis + path number) on individual trials and examined which of our hypotheses (Fig 7, S2 Table) fit the observed pointing directions for each participant. To include more data points in this analysis, we did not exclude any trials. The mean fit ratio was summarized in Fig 12. Most participants showed the same model fit results as the group-level analysis, showing that the observed pointing directions in TI-C condition were more consistent with the Cross to Cross (CtoC) hypothesis. In contrast, the pointing directions in FI-NC condition were again more consistent with the No cross to No cross 1 (NtoN1) hypothesis. Individual differences, however, were observed as some participants were more consistent with the No cross to Cross (NtoC3) hypothesis and non-Euclidean distortions. For the TI-C condition, a few participants’ pointing responses (see the outlier dots in column “CtoN1” in Fig 12) were more consistent with the Cross to No cross 1 (CtoN1) hypothesis than the CtoC hypothesis, which suggests significant spatial memory distortions. Specifically, our path shape models suggested that these participants still underestimated L1 even when there was minimal conflicting information (TI-C condition) and no influence from priming trials in another condition (see also Experiment 1b). We return to this issue in the general discussion.

The authors provide the following additional analysis of these updated results: A repeated-measure ANOVA using the model fit ratio for the NtoN1 hypothesis indicates that there was a significant difference between the four paths, *F*(1, 3) = 52.76, *p* < .001, *η*_*p*_^2^ = 0.87 (large effect). Planned contrasts showed that the ratios in Path 5 and 6 were significantly higher than those in Path 7 and 8, *t*(42) = 7.22, *p* < .001, Cohen’s *d* = 1.56. This sugges*t*s that some paths resulted in more non-Euclidean distortions than others for the FI-NC condition.

In Section C of S1 Text, the absolute AE_G_ in Experiment 2 and 3 was analyzed (please see the correct S6 Fig, Panel Experiment 2 and 3).

In S1 Text, there were errors in the AE_G_ analysis of Experiments 2 and 3 as a result of the coding errors. These have been addressed in a new, correct version of S1 Text, which is provided with this Correction.

The coding error impacts results in Figs 8, 10–12, S6, S7, S11 as well as Tables 2, 3, S4 and S7. Please see the correct Figs 8, 10–12, S6, S7, and S11, and the correct Tables 2, 3, S4 and S7 included with this Correction.

A member of the Editorial Board reviewed the updated figures and underlying data and determined that the conclusions in [1] are still supported. The corrected analyses do not alter the central interpretation that false visual intersections lead to systematic spatial memory distortions.

**Fig 8:**
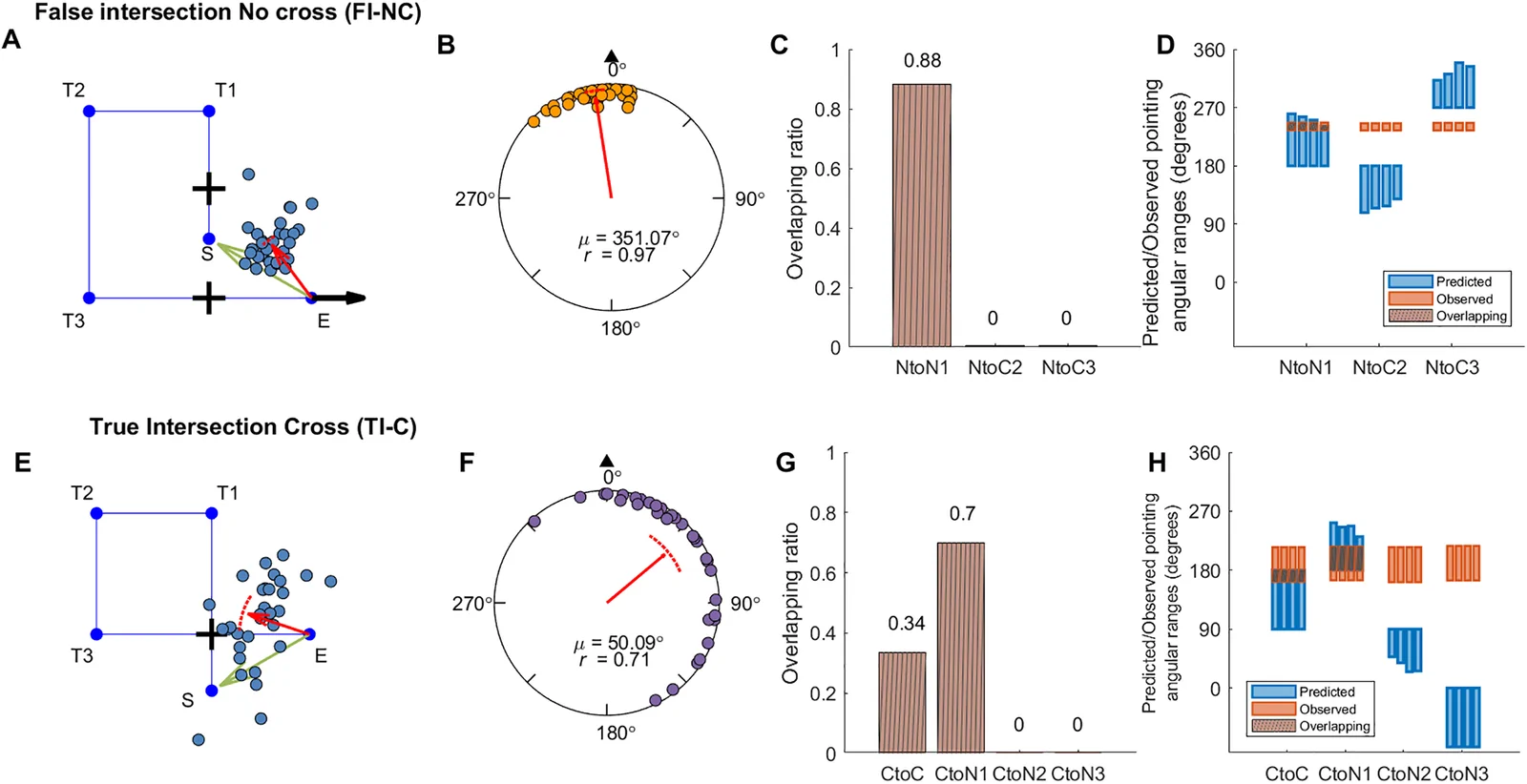
Response pointing directions and hypothesis comparison results in Experiment 2 (FI-NC and TI-C conditions). Panel A, E: Example pointing locations in one path (A: Path 8; E: Path 4). The red arrow indicates the circular mean direction and the distance of the pointing across all participants. Panel B, F: Mean pointing error (AE_G_) across paths for each condition. Panel C, G: Mean overlapping ratio between observed and predicted pointing ranges across paths for each hypothesis (Fig 7, S2 Table). Panel D, H: Predicted or observed pointing angular ranges (in degrees) for individual paths. Abbreviations and legend are the same as in Fig 3.

**Fig 10:**
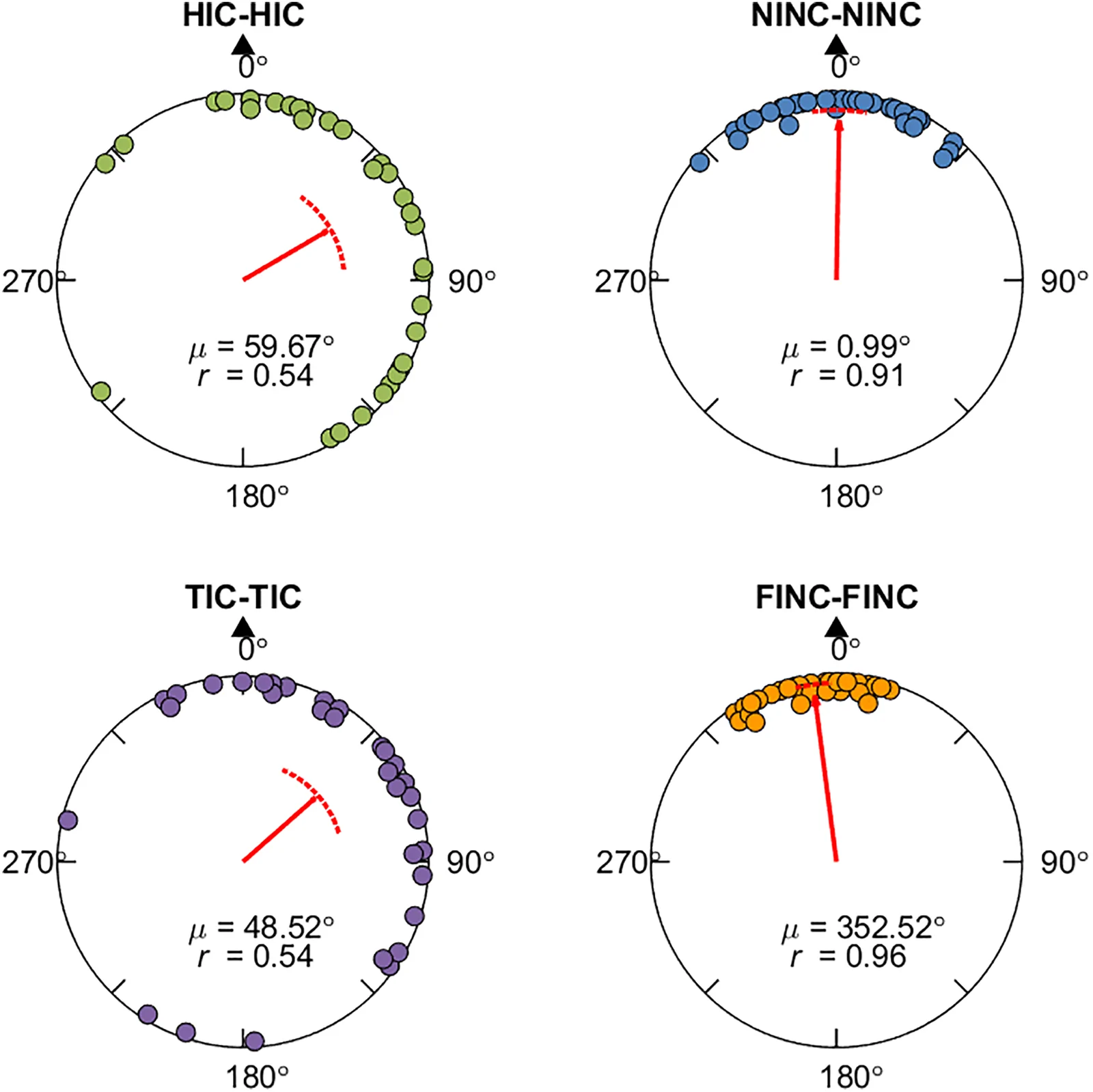
Mean pointing error (AE_G_) across paths for each condition in Experiment 2 with only the trials primed by the same condition. HIC-HIC: HI-C to HI-C condition only. NINC-NINC: NI-NC to NI-NC condition only. TIC-TIC: TI-C to TI-C condition only. FINC-FINC: FI-NC to FI-NC condition only. Abbreviations and legend are the same as in Fig 3.

**Fig 11:**
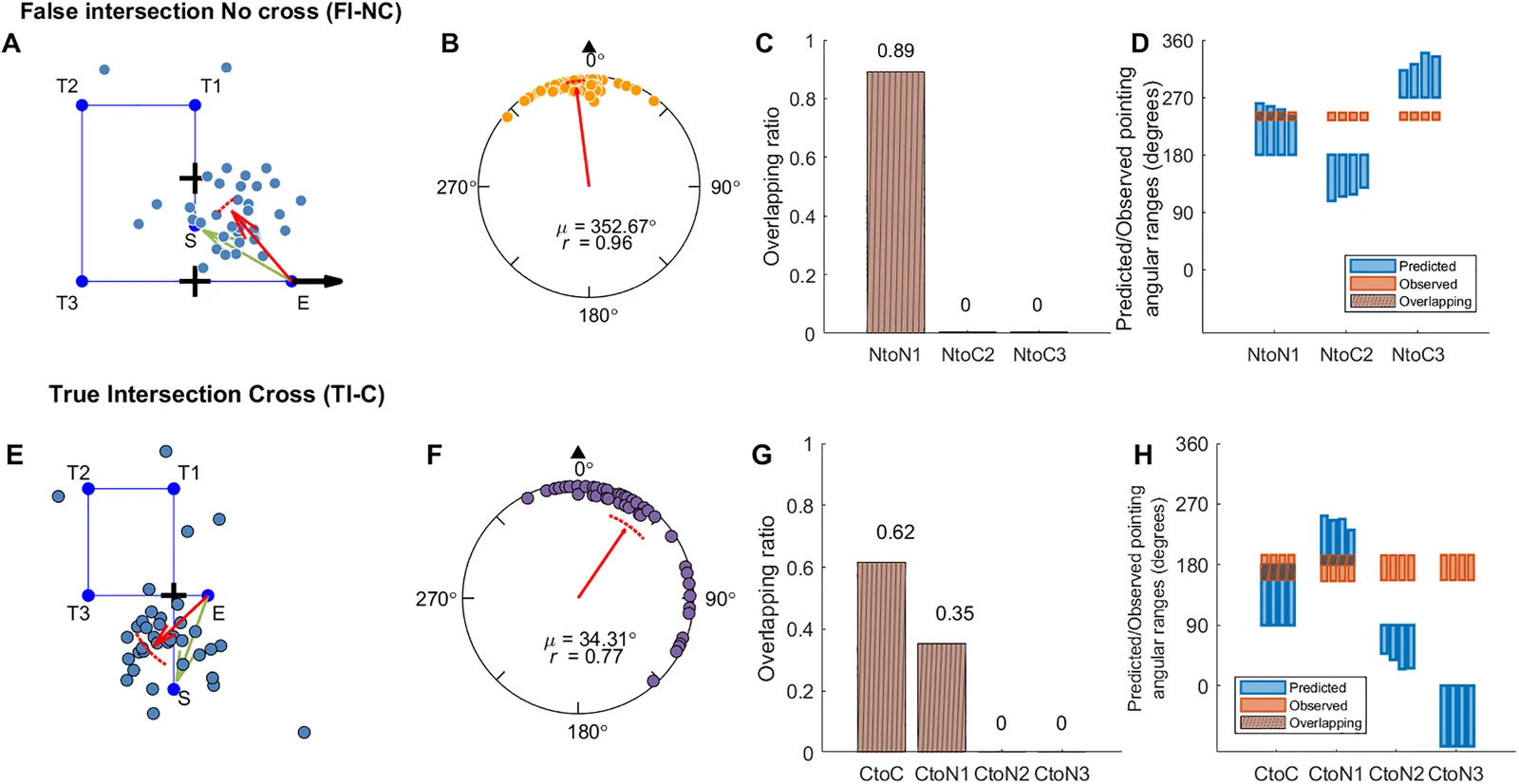
Response pointing directions and hypothesis comparison results in Experiment 3. Panel A, E: Example pointing directions in one path (A: Path 8; E: Path 1). The red arrow indicates the circular mean direction and the distance of the pointing across all participants. Panel B, F: Mean pointing error (AE_G_) across paths for each condition. Panel C, G: Mean overlapping ratio between observed and predicted pointing ranges across paths for each hypothesis (Fig 7, S2 Table). Panel D, H: Predicted or observed pointing angular ranges (in degrees) for individual paths. Abbreviations and legend are the same as in Fig 3.

**Fig 12:**
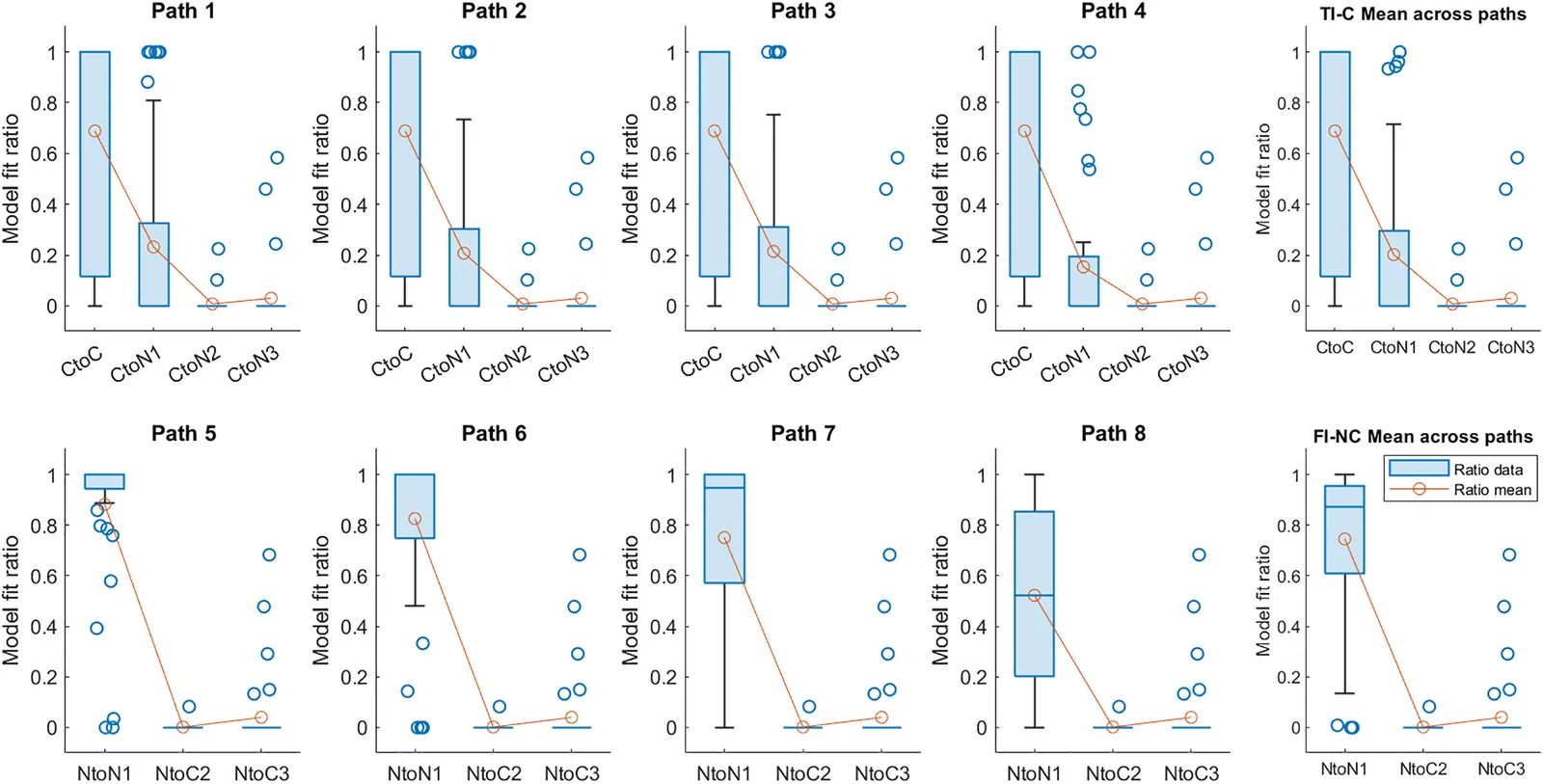
Distribution of model fit ratios for individual participants in Experiment 3. Orange circles show the mean ratios of each hypothesis. Box boundaries show interquartile ranges. Lines inside box show medians. Lower and upper error lines show non-outlier minimum and maximum values, respectively.

**Table 2:** Outlier proportions and kappa values in outlier detection procedure in Experiments 1-3.

Experiment	Condition	Observed kappa before exclusion	Kappa used in simulation	Observed kappa after exclusion	Outlier datapoints number	Outlier proportion
1a (hallway mixed-design)	Hidden intersection Cross (HI-C)	0.51	0.7	0.74	10	7.81%
	No intersection No cross (NI-NC)	1.71	3.5	2.04	5	2.93%
1b (pole-guided)	Pole-guided cross (PG-C)	2.33	4	2.54	2	2.38%
	Pole-guided No cross (PG-NC)	1.03	1.8	1.34	7	8.33%
1c (hallway blocked-design)	Hidden intersection Cross (HI-C)	0.74	0.75	0.91	6	4.84%
	No intersection No cross (NI-NC)	1.32	2.1	1.66	7	5.65%
2	Hidden intersection Cross (HI-C)	1.16	2	1.44	7	5.47%
	True intersection Cross (TI-C)	1.38	2	1.67	6	4.69%
	No intersection No cross (NI-NC)	7.04	9	7.04	0	0
	False intersection No cross (FI-NC)	7.78	9	7.78	0	0
3	True intersection Cross (TI-C)	1.87	3.2	2.16	4	3.45%
	False intersection No cross (FI-NC)	7.84	9	8.96	3	0.58%

**Table 3:** Summary of results in all experiments.

Experiment	Condition	Best fit hypothesis
1a (hallway mixed design)	Hidden intersection Cross (HI-C)	Crossed paths to uncrossed paths (CtoN1)
	No intersection No cross (NI-NC)	Uncrossed paths remain uncrossed (NtoN1)
1b (pole-guided)	Pole-guided cross (PG-C)	Crossed paths remain crossed (CtoC)
	Pole-guided No cross (PG-NC)	Uncrossed paths remain uncrossed (NtoN1)
1c (hallway blocked design)	Hidden intersection Cross (HI-C)	Crossed paths to uncrossed paths (CtoN1)
	No intersection No cross (NI-NC)	Uncrossed paths remain uncrossed (NtoN1)
2 (hallway mixed design)	Hidden intersection Cross (HI-C)	Crossed paths remain crossed (CtoC)
	True intersection Cross (TI-C)	Crossed to uncrossed paths (CtoN1)
	No intersection No cross (NI-NC)	Uncrossed paths remain uncrossed (NtoN1)
	False intersection No cross (FI-NC)	Uncrossed paths remain uncrossed (NtoN1)
3 (hallway blocked design)	True intersection Cross (TI-C)	Ambiguous fit (CtoC and CtoN1)
	False intersection No cross (FI-NC)	Uncrossed paths remain uncrossed (NtoN1)
4 (desktop hallway blocked design)	Same as Exp 2	N/A

## Supporting information

S6 Fig Absolute angular pointing errors (AE_G_). Error bars show the standard errors of the mean in each condition.
(TIF)

S7 Fig TI-C: True Intersection Cross condition. FI-NC: False Intersection No cross condition. The red arrow indicates the circular mean direction (μ) and the distance of the pointing across all participants. Other abbreviations and legend are the same as in S1 Fig.
(TIF)

S11 Fig Response pointing positions in Experiment 3. Abbreviations and legend are the same as in S7 Fig.
(TIF)

S4 Table Random effects in circular regression. Posterior mode and 95% highest posterior density (HPD) lower (LB) and upper bounds (UB) of the random effect variances in regression for each group and condition.
(DOCX)

S7 Table Circular regression results in Experiment 3. Posterior modes and 95% highest posterior density (HPD) lower (LB) and upper bounds (UB) for the regression coefficients of the angular error for each group and condition in Experiment 3.
(DOCX)

S1 Text Supplementary results. (DOCX)
