# Supplementary figures and images for: Spatial memory distortions for the shapes of walked paths occur in violation of physically experienced geometry
Source: PLoS One. 2023 Feb 10;18(2):e0281739. doi: 10.1371/journal.pone.0281739 (PMC9916584; doi:10.1371/journal.pone.0281739)

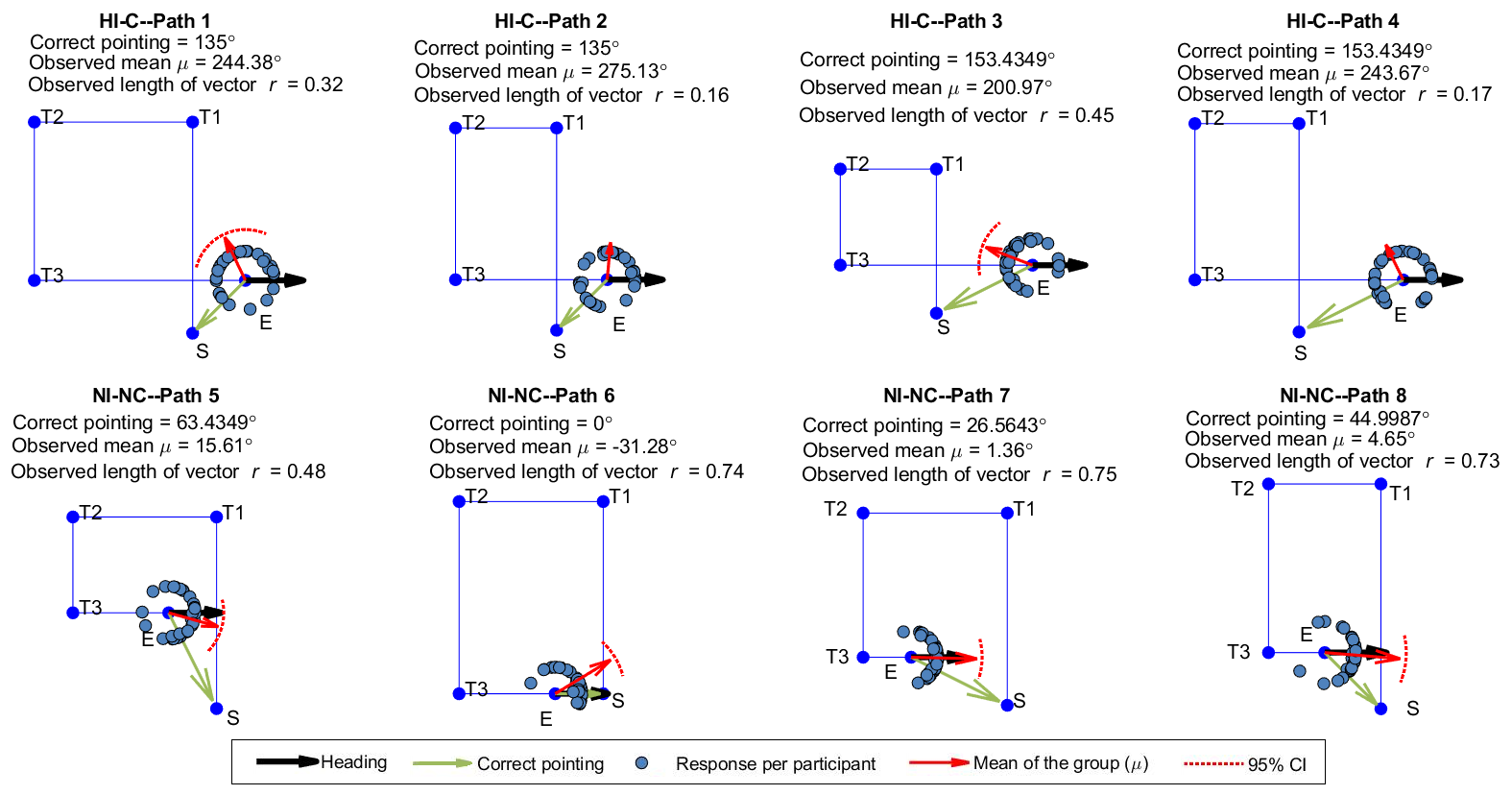

Supplement: S1 Fig — HI-C: hidden intersection cross condition. NI-NC: no intersection no cross condition. Each dot indicates the pointing direction for one participant. The red arrow indicates the circular mean direction (μ) of the pointing directions across all participants. r is the mean resultant length of all pointing angles. The arc above the mean direction indicates the 95% confidence interval of the mean direction. (TIF) [file pone.0281739.s001.tif]

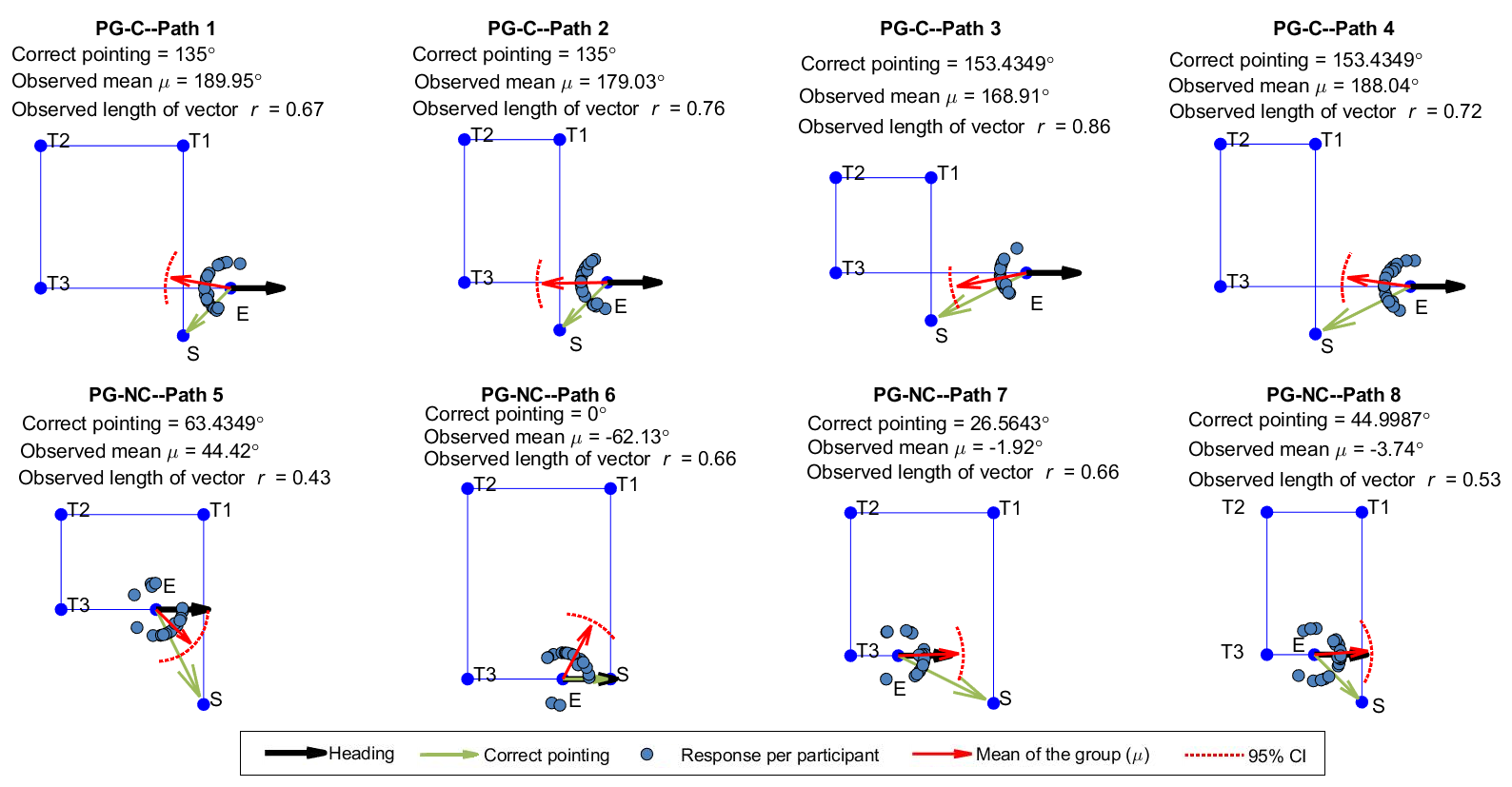

Supplement: S2 Fig — PG-C: Pole-guided Cross condition. PG-NC: Pole-guided No cross condition. Legend is the same as in S1 Fig. (TIF) [file pone.0281739.s002.tif]

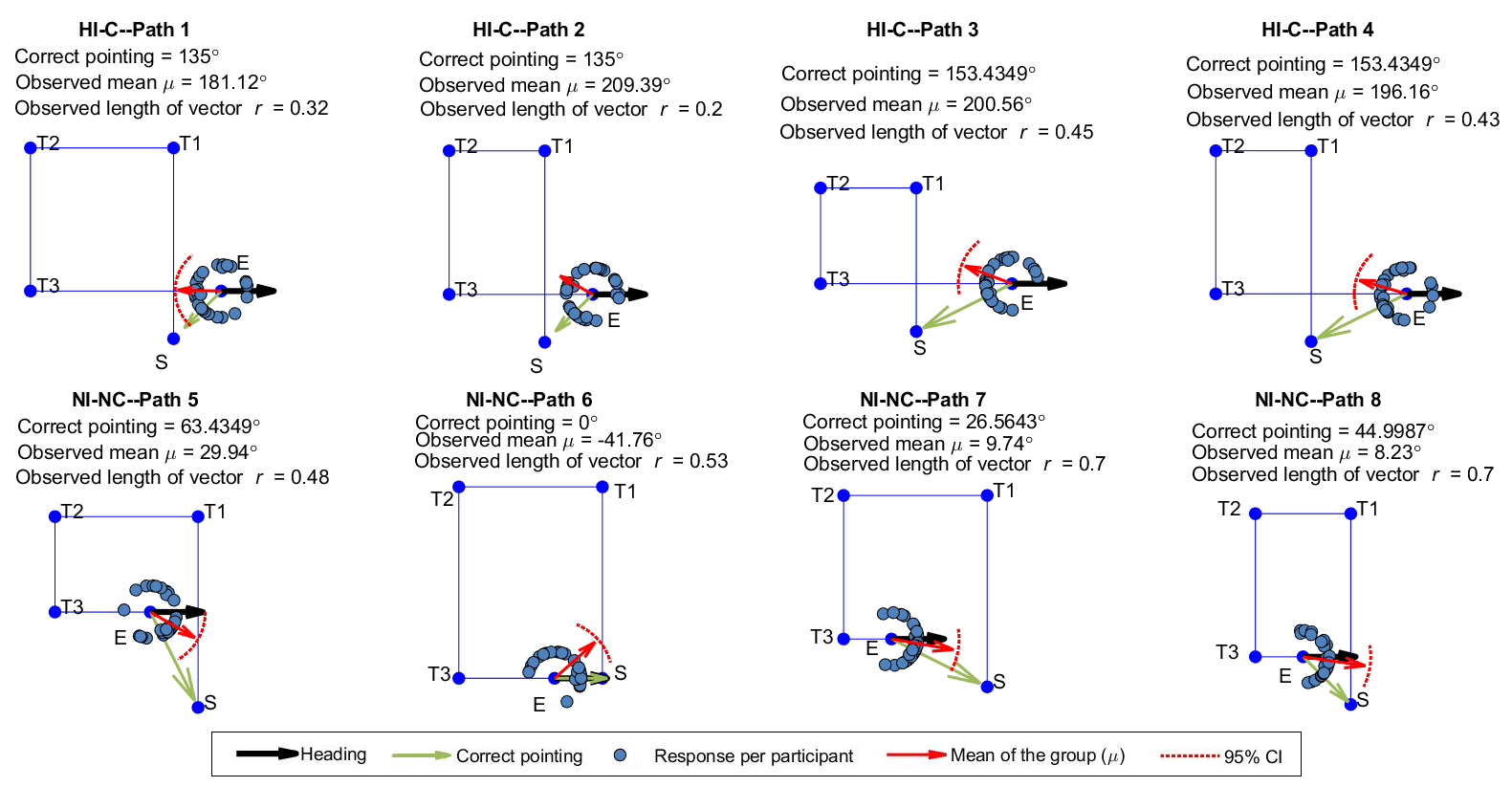

Supplement: S3 Fig — Abbreviations and legend are the same as in S1 Fig. (TIF) [file pone.0281739.s003.tif]

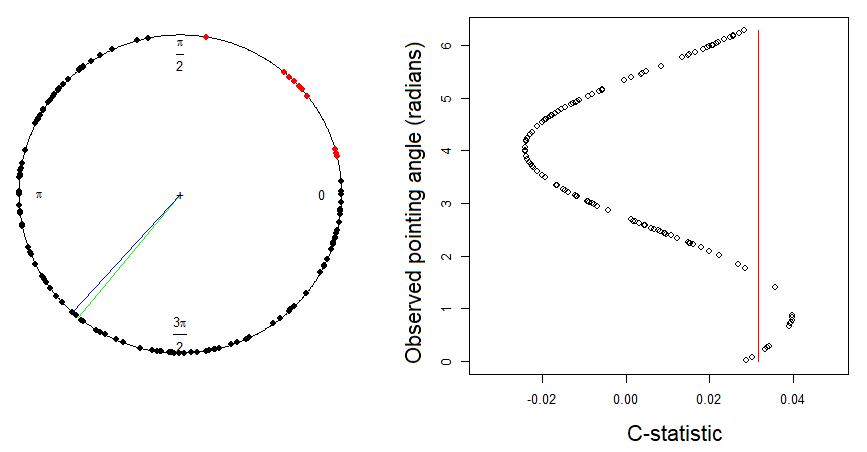

Supplement: S4 Fig — Left: Each dot indicates the circular mean across trials with the same path shape for each participant. The green and blue lines indicate the mean direction of the sample before and after excluding outliers, respectively. Red dots indicate the outliers in this sample. Right: Observed pointing angles as a function of c-statistics. The red vertical line indicates the cut-off value of c-statistic in this sample. The dots at the right side of the red line indicate the outliers in this sample and correspond to the red dots on the left panel. (TIF) [file pone.0281739.s004.tif]

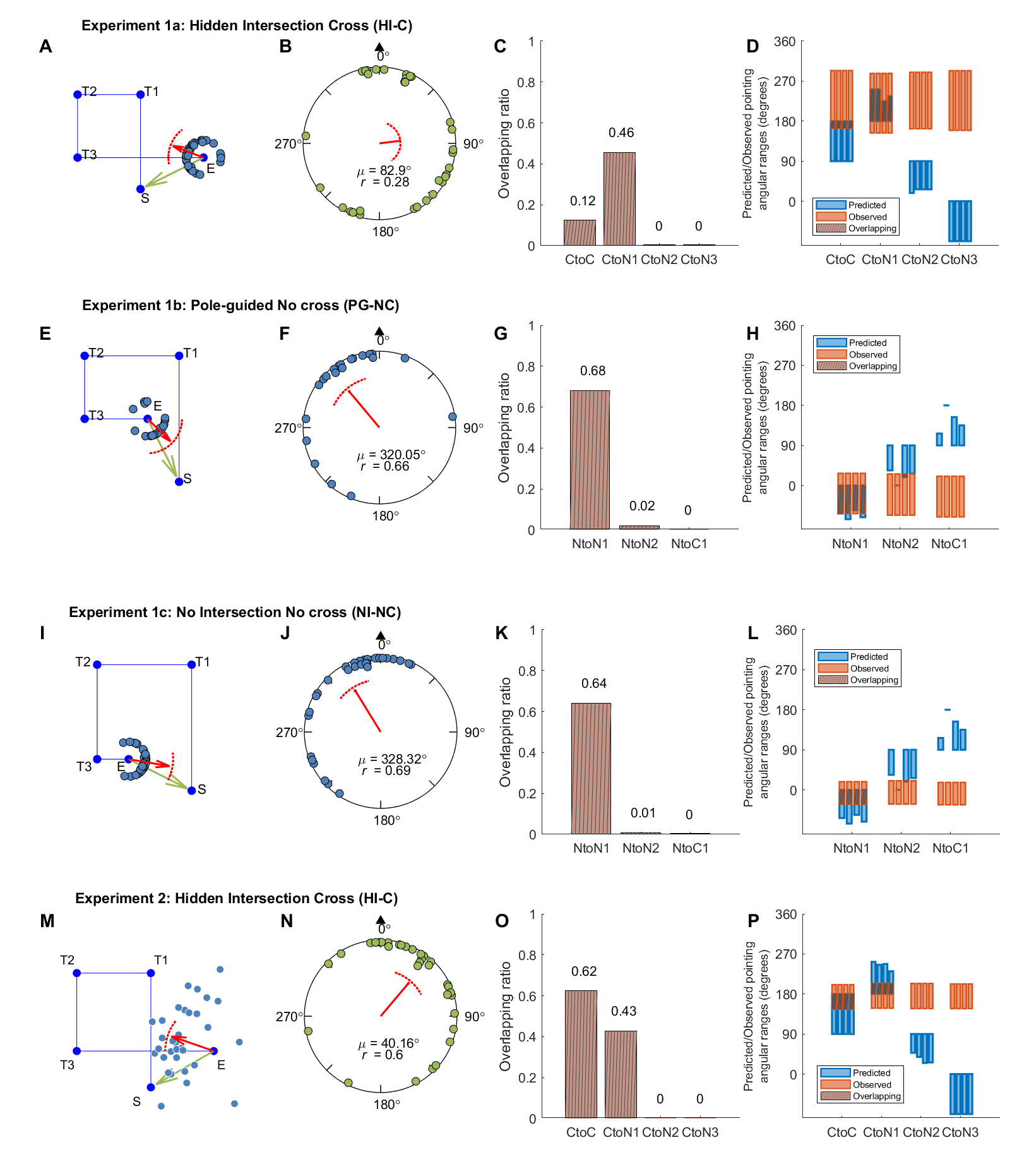

Supplement: S5 Fig — Panel A, E, I, M: Example pointing directions in one path. A: Path 3. E: Path 5. I: Path 7. M: Path 4. Panel B, F, J, N: Mean pointing error across paths (AEG). Panel C, G, K, O: Mean overlapping ratio between observed and predicted pointing ranges across paths for each hypothesis (see Figs 1 and 7 and S2 Table). Panel D, H, L, P: Predicted or observed pointing angular ranges (in degrees) for individual paths. Abbreviations and legend are the same as in Fig 3. (TIF) [file pone.0281739.s005.tif]

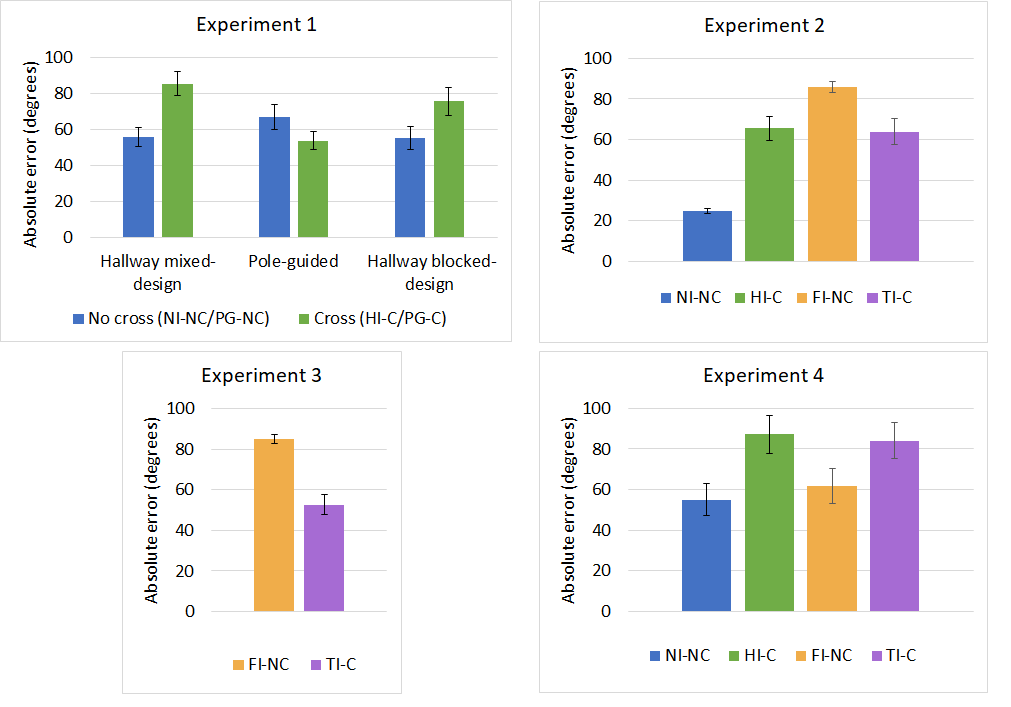

Supplement: S6 Fig — Error bars show the standard errors of the mean in each condition. (TIF) [file pone.0281739.s006.tif]

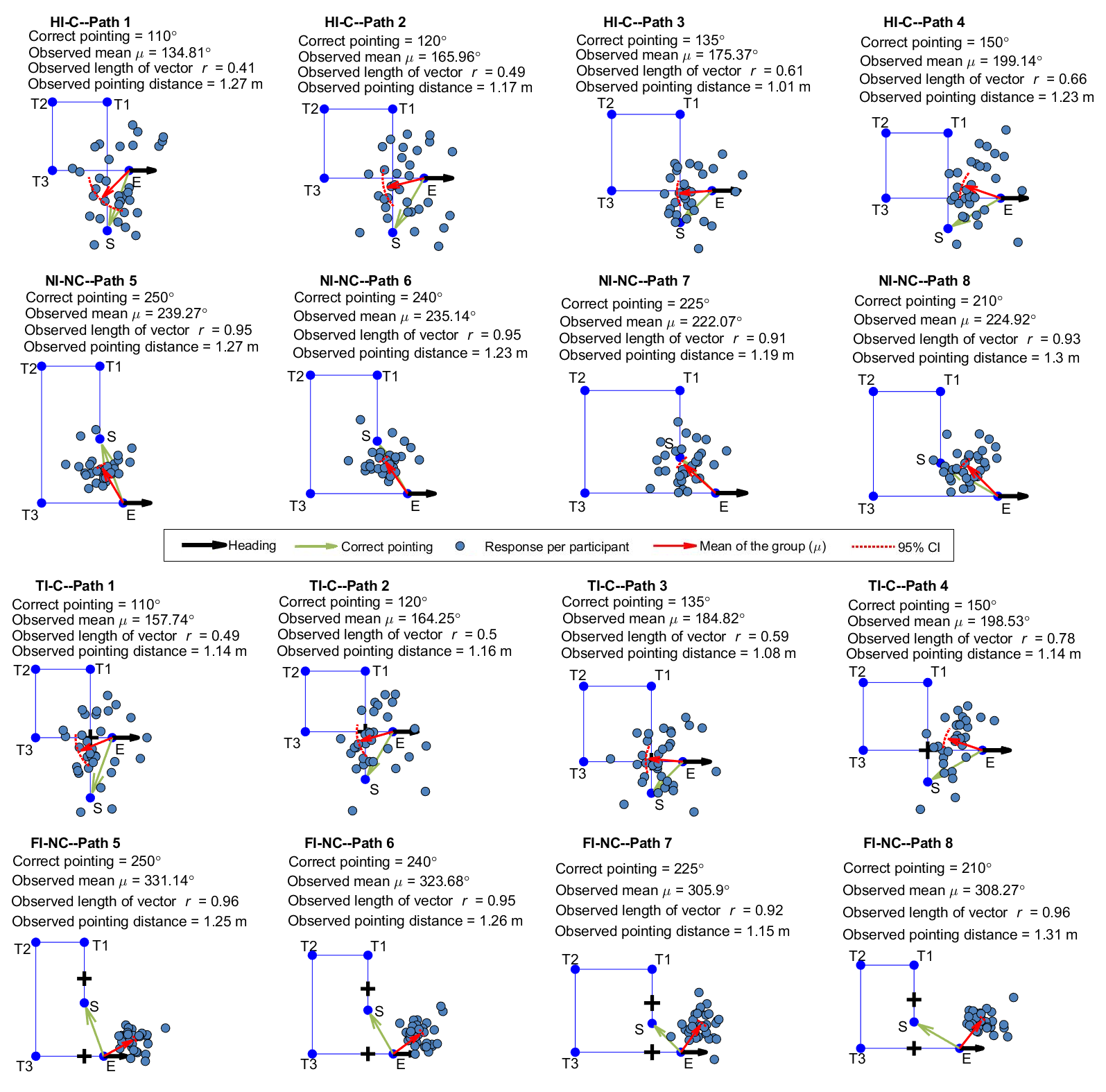

Supplement: S7 Fig — TI-C: True Intersection Cross condition. FI-NC: False Intersection No cross condition. The red arrow indicates the circular mean direction (μ) and the distance of the pointing across all participants. Other abbreviations and legend are the same as in S1 Fig. (TIF) [file pone.0281739.s007.tif]

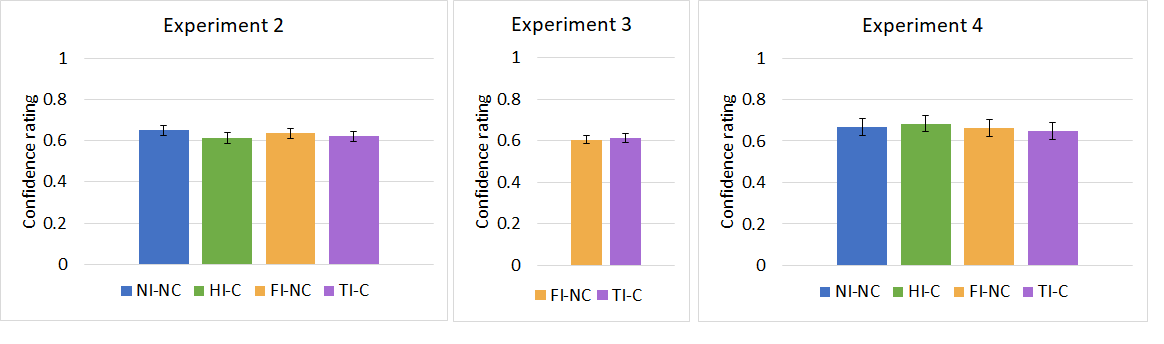

Supplement: S8 Fig — Error bars show the standard errors of the mean in each condition. (TIF) [file pone.0281739.s008.tif]

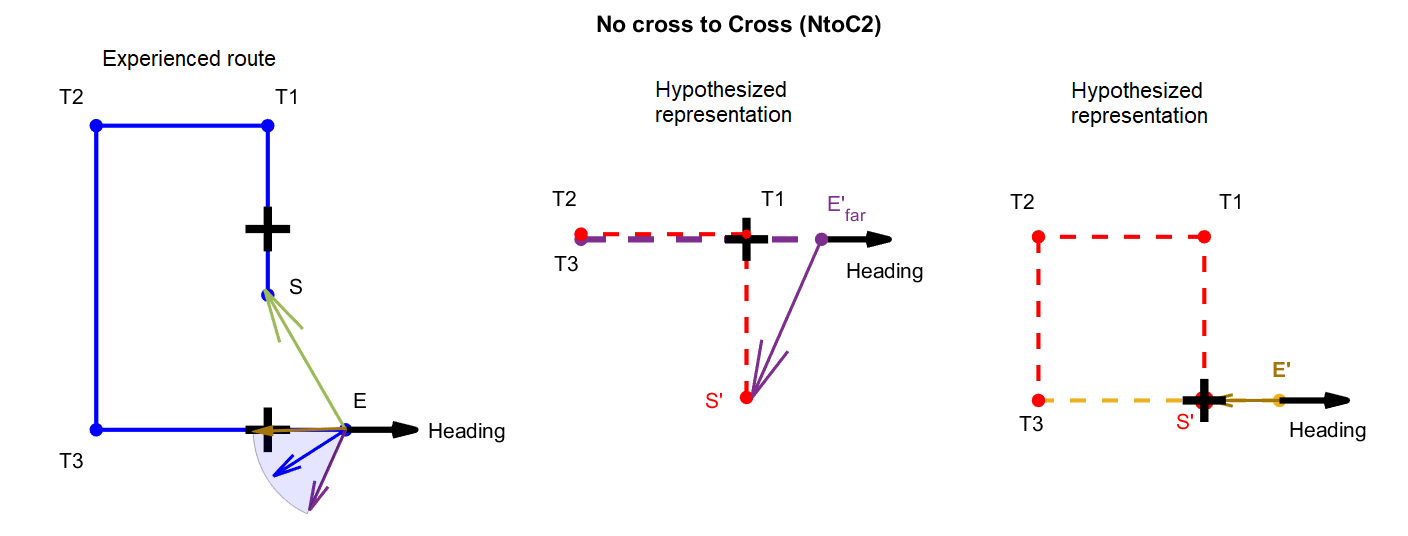

Supplement: S9 Fig — E’far: the estimated position of the end if the intersection is estimated to overlap with T1. Other abbreviations and legend are the same as in Fig 7. (TIF) [file pone.0281739.s009.tif]

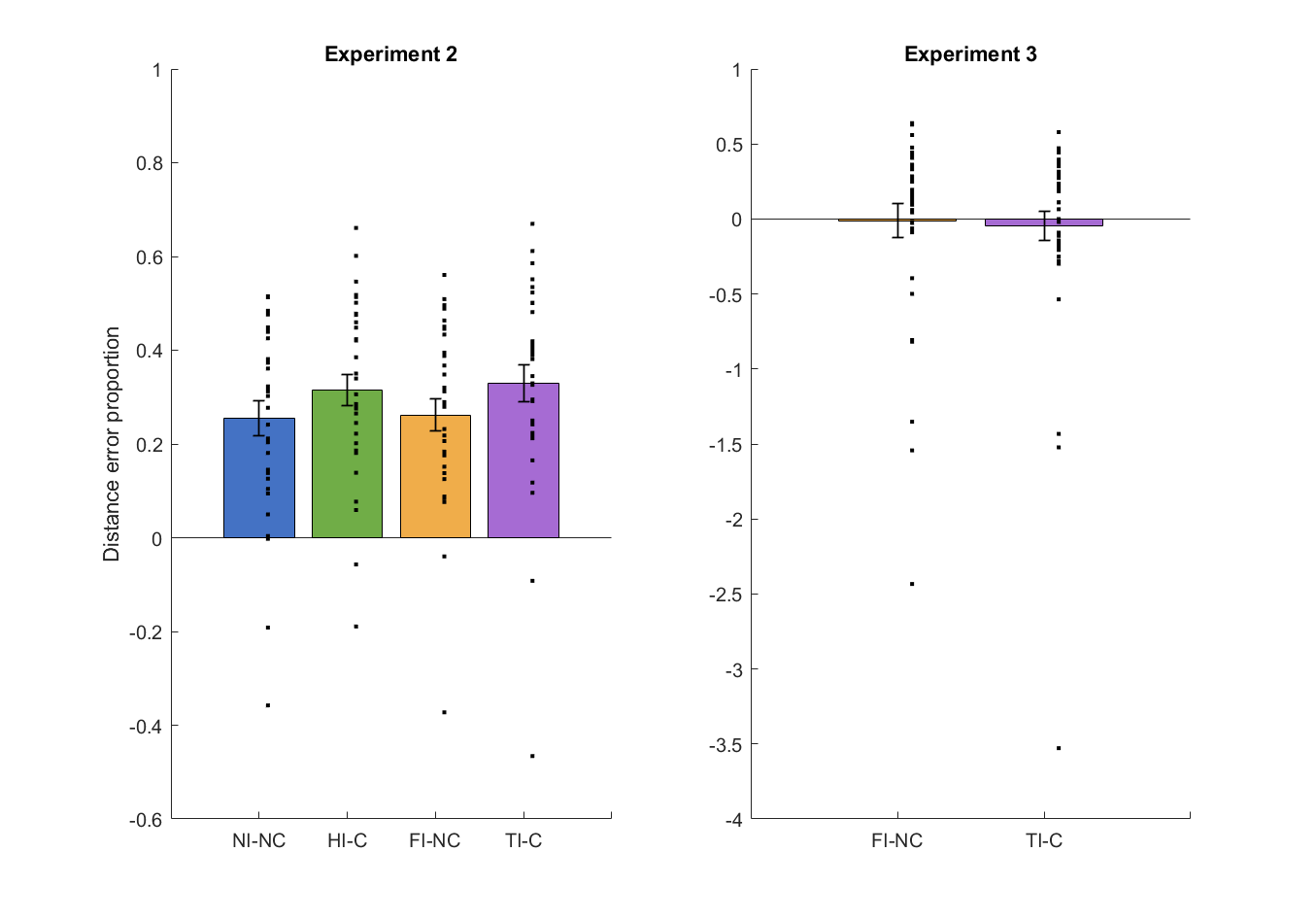

Supplement: S10 Fig — Error bars show the standard errors of the mean in each condition. The dots show the data of individual participants. (TIF) [file pone.0281739.s010.tif]

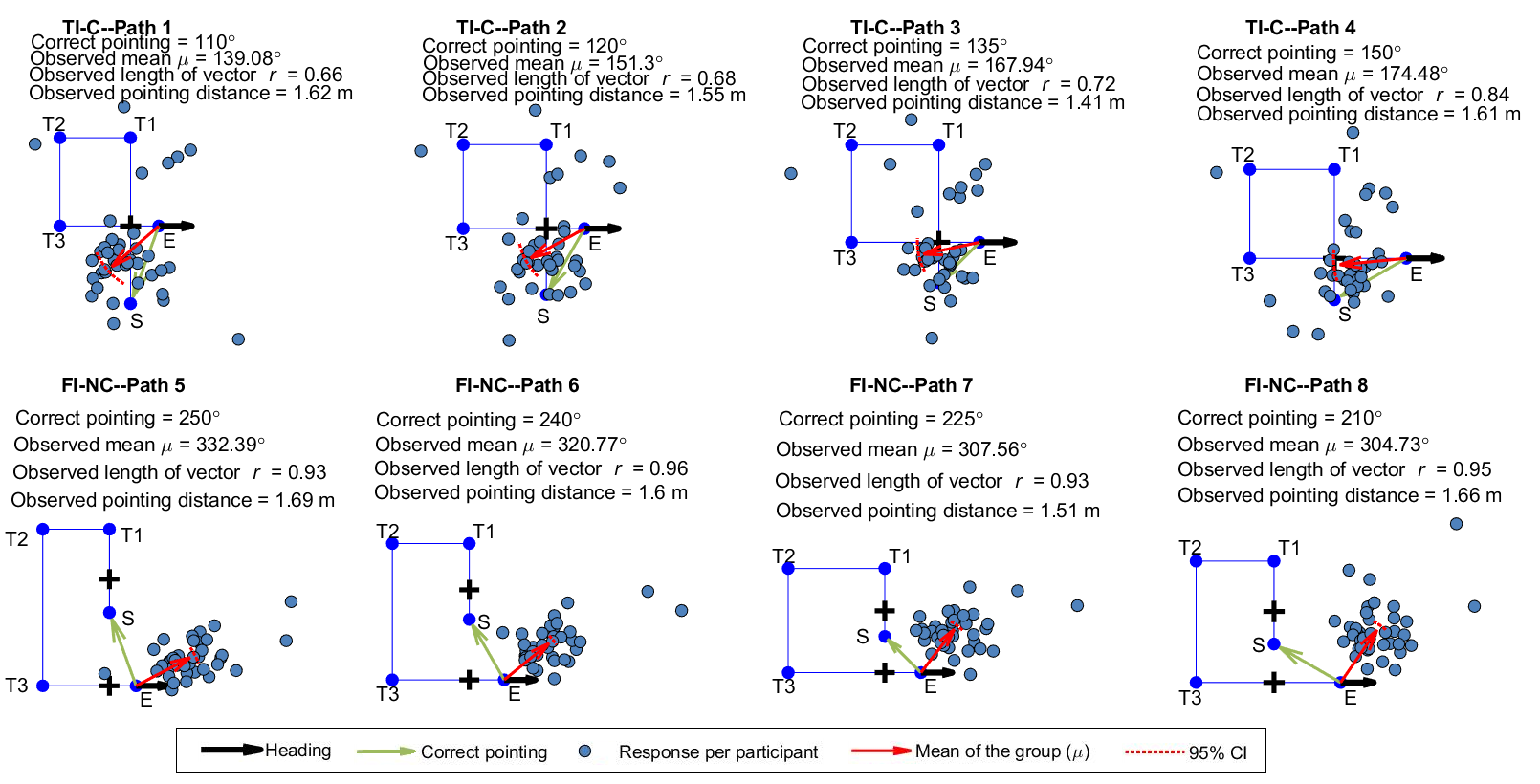

Supplement: S11 Fig — Abbreviations and legend are the same as in S7 Fig. (TIF) [file pone.0281739.s011.tif]

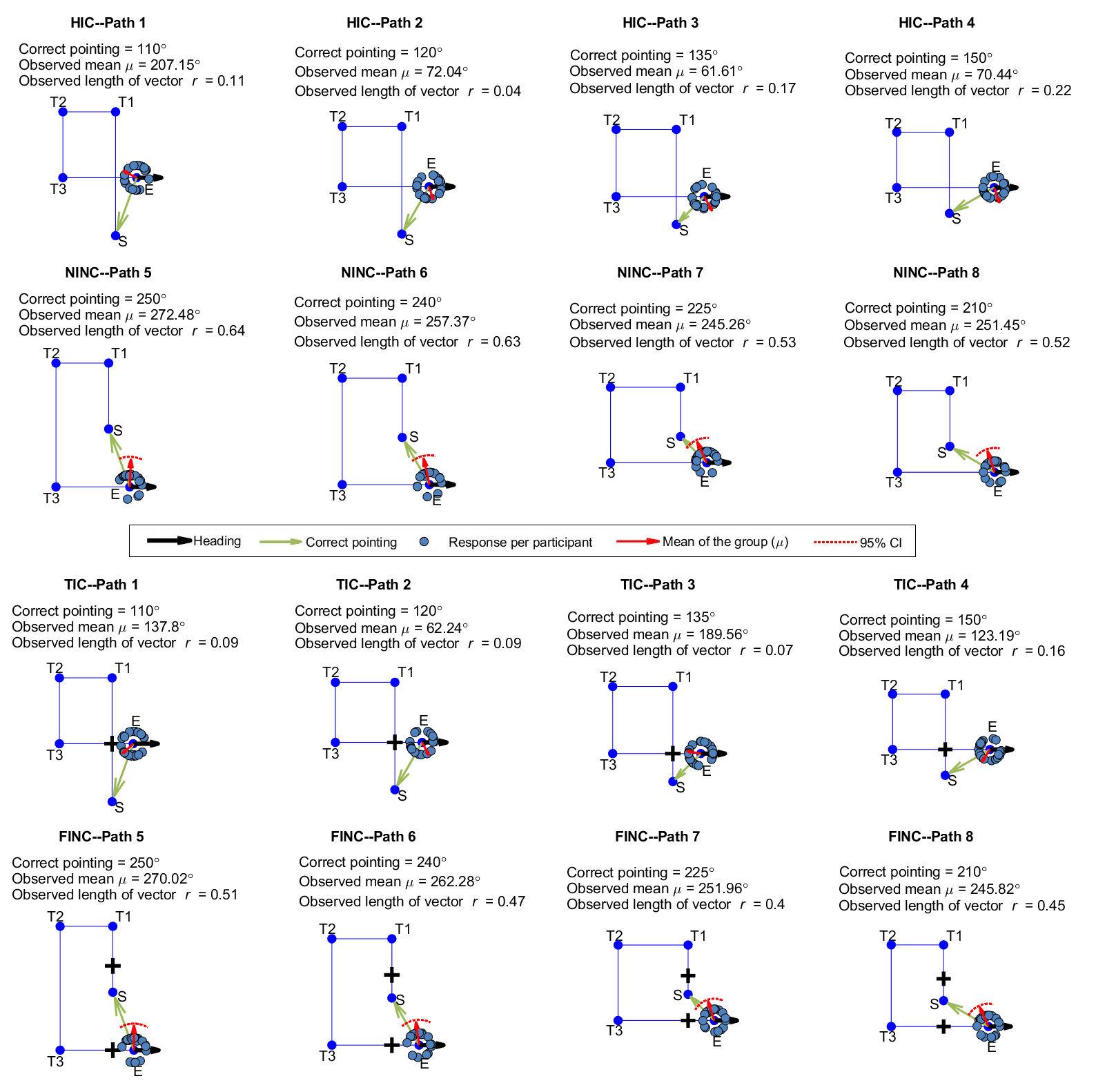

Supplement: S12 Fig — Abbreviations and legend are the same as in S7 Fig. (TIF) [file pone.0281739.s012.tif]
