# Supplementary material for: Spatial memory distortions for the shapes of walked paths occur in violation of physically experienced geometry
Source: PLoS One. 2023 Feb 10;18(2):e0281739. doi: 10.1371/journal.pone.0281739 (PMC9916584; doi:10.1371/journal.pone.0281739)
Supplement: S1 Table — (DOCX) [file pone.0281739.s013.docx]

S1 Table. *Path dimensions (in meters) in Experiment 1.*

| **Path** | **Path type** | **L1** | **L2** | **L3** | **L4** | **Total length** |
| --- | --- | --- | --- | --- | --- | --- |
| 1 | Cross | 3.4 | 2.55 | 2.55 | 3.4 | 11.9 |
| 2 | Cross | 3.4 | 1.7 | 2.55 | 2.55 | 10.2 |
| 3 | Cross | 2.55 | 1.7 | 1.7 | 3.4 | 9.35 |
| 4 | Cross | 3.4 | 1.7 | 2.55 | 3.4 | 11.05 |
| 5 | No cross | 3.4 | 2.55 | 1.7 | 1.7 | 9.35 |
| 6 | No cross | 3.4 | 2.55 | 3.4 | 1.7 | 11.05 |
| 7 | No cross | 3.4 | 2.55 | 2.55 | 0.85 | 9.35 |
| 8 | No cross | 3.4 | 1.70 | 2.55 | 0.85 | 8.50 |

*Note*: L1: From the start (S) to the first turning point (T1). L2: From T1 to the second turning point (T2). L3: From T2 to the third turning point (T3). L4: From T3 to the end (E). See Figure 1 for more details.
