# Supplementary material for: Spatial memory distortions for the shapes of walked paths occur in violation of physically experienced geometry
Source: PLoS One. 2023 Feb 10;18(2):e0281739. doi: 10.1371/journal.pone.0281739 (PMC9916584; doi:10.1371/journal.pone.0281739)
Supplement: S2 Table — (DOCX) [file pone.0281739.s014.docx]

S2 Table. *Predicted angular ranges and mean (in degrees).*

| **Experiment 1** | | | | | | | | | | |
| --- | --- | --- | --- | --- | --- | --- | --- | --- | --- | --- |
| **Path** | **Path type** | **G** | **CtoC range** | **CtoC mean** | **CtoN1 range** | **CtoN1 mean** | **CtoN2 range** | **CtoN2 mean** | **CtoN3 mean** | **CtoN3 range** |
| 1 | Cross | 135° | [90°, 180°] | 135.14° | [180°, 251.57°] | 215.77° | [18.44°, 90°] | 54.07 | [-90°, 0°] | -44.94° |
| 2 | Cross | 135° | [90°, 180°] | 135.03° | [180°, 251.57°] | 215.79° | [26.57°, 90°] | 58.35 | [-90°, 0°] | -44.89° |
| 3 | Cross | 153.43° | [90°, 180°] | 134.90° | [180°, 225°] | 202.48° | [26.57°, 90°] | 58.13 | [-90°, 0°] | -44.87° |
| 4 | Cross | 153.43° | [90°, 180°] | 135.03° | [180°, 236.31°] | 208.01° | [26.57°, 90°] | 58.39 | [-90°, 0°] | -44.96° |
| **Path** | **Path type** | **G** | **NtoN1 range** | **NtoN1 mean** | **NtoN2 range** | **NtoN2 mean** | **NtoC1 range** | **NtoC1 mean** |  |  |
| 5 | No cross | 63.43° | [-63.43°, 0°] | -31.68° | [33.69°, 90°] | 61.85° | [90°, 116.56°] | 103.33° |  |  |
| 6 | No cross | 0° | [-75.96°, 0°] | -37.72° | [0°, 0°] | 0° | [180°, 180°] | 180° |  |  |
| 7 | No cross | 26.56° | [-56.31°, 0°] | -28.11° | [18.43°, 90°] | 54.32° | [90°, 153.44°] | 121.61° |  |  |
| 8 | No cross | 45° | [-71.56°, 0°] | -35.92° | [26.56°, 90°] | 58.48° | [90°, 135.00°] | 112.43° |  |  |
| **Experiment 2 and 3** | | | | | | | | | | |
| **Path** | **Path type** | **G** | **CtoC range** | **CtoC mean** | **CtoN1 range** | **CtoN1 mean** | **CtoN2 range** | **CtoN2 mean** | **CtoN3 range** | **CtoN3 mean** |
| 1 | Cross | 110° | [90°, 180°] | 135.17° | [180°, 252.24°] | 216.25° | [47.71°, 90°] | 68.75° | [-90°, 0°] | -45.03° |
| 2 | Cross | 120° | [90°, 180°] | 135.27° | [180°, 245.57°] | 212.77° | [38.48°, 90°] | 64.26° | [-90°, 0°] | -45.19° |
| 3 | Cross | 135° | [90°, 180°] | 135.17° | [180°, 247.37°] | 213.72° | [24.94°, 90°] | 57.54° | [-90°, 0°] | -45.31° |
| 4 | Cross | 150° | [90°, 180°] | 134.77° | [180°, 231.07°] | 205.45° | [26.12°, 90°] | 58.12° | [-90°, 0°] | -44.80° |
| **Path** | **Path type** | **G** | **NtoN1 range** | **NtoN1 mean** | **NtoC2 range** | **NtoC2 mean** | **NtoC3 range** | **NtoC3 mean** |  |  |
| 5 | No cross | 250° | [180°, 260.33°] | 220.35° | [107.76°, 180°] | 143.85 | [270°, 312.29°] | 291.15° |  |  |
| 6 | No cross | 240° | [180°, 255.65°] | 217.83° | [114.68°, 180°] | 147.40 | [270°, 321.85°] | 295.98° |  |  |
| 7 | No cross | 225° | [180°, 250.85°] | 215.52° | [118.02°, 180°] | 149.06 | [270°, 339.47°] | 304.46° |  |  |
| 8 | No cross | 210° | [180°, 241.26°] | 210.81° | [128.75°, 180°] | 154.24 | [270°, 333.73°] | 301.73° |  |  |

*Note*: G: Correct pointing directions according to Euclidean geometry. See Figure 1 and 7 for details about the hypotheses.
