# Supplementary material for: Spatial memory distortions for the shapes of walked paths occur in violation of physically experienced geometry
Source: PLoS One. 2023 Feb 10;18(2):e0281739. doi: 10.1371/journal.pone.0281739 (PMC9916584; doi:10.1371/journal.pone.0281739)
Supplement: S3 Table — Posterior modes and 95% highest posterior density (HPD) lower (LB) and upper bounds (UB) for the regression coefficients of the angular error for each group and condition. (DOCX) [file pone.0281739.s015.docx]

S3 Table. *Posterior modes and 95% highest posterior density (HPD) lower (LB) and upper bounds (UB) for the regression coefficients of the angular error for each group and condition in Experiment 1.*

| **Group** | **Condition** | **Hypothesis** | **Component I** | | | **Component II** | | |
| --- | --- | --- | --- | --- | --- | --- | --- | --- |
|  |  |  | **Mode** | **LB HPD** | **UB HPD** | **Mode** | **LB HPD** | **UB HPD** |
| Hallway mixed-design | HI-C | CtoC | -0.07 | -0.32 | 0.36 | -0.01 | -0.33 | 0.31 |
|  |  | CtoN1 | -0.89 | -1.71 | 0.24 | -1.09 | -2.01 | 0.03 |
|  |  | CtoN2 | 0.03 | -0.31 | 0.35 | -0.01 | -0.36 | 0.30 |
|  |  | CtoN3 | -0.02 | -0.31 | 0.34 | 0.05 | -0.30 | 0.33 |
|  | NI-NC | NtoN1 | 2.74 | 1.88 | 3.62 | -0.19 | -0.83 | 0.41 |
|  |  | NtoN2 | -0.09 | -0.36 | 0.35 | 0.05 | -0.29 | 0.30 |
|  |  | NtoC1 | -0.04 | -0.36 | 0.37 | -0.0004 | -0.32 | 0.28 |
| Pole-guided | PG-C | CtoC | -0.09 | -0.43 | 0.44 | 0.07 | -0.37 | 0.34 |
|  |  | CtoN1 | -2.35 | -2.87 | -1.93 | 0.10 | -0.64 | 0.94 |
|  |  | CtoN2 | -0.001 | -0.44 | 0.47 | -0.06 | -0.36 | 0.35 |
|  |  | CtoN3 | -0.01 | -0.39 | 0.46 | 0.02 | -0.38 | 0.35 |
|  | PG-NC | NtoN1 | 0.97 | 0.43 | 1.64 | -0.50 | -1.32 | 0.62 |
|  |  | NtoN2 | 0.01 | -0.42 | 0.39 | 0.04 | -0.40 | 0.38 |
|  |  | NtoC1 | -0.02 | -0.35 | 0.36 | 0.05 | -0.37 | 0.40 |
| Hallway blocked-design | HI-C | CtoC | 0.04 | -0.36 | 0.34 | -0.04 | -0.33 | 0.33 |
|  |  | CtoN1 | -1.58 | -2.88 | -0.13 | -0.79 | -2.22 | 0.64 |
|  |  | CtoN2 | 0.07 | -0.37 | 0.34 | 0.02 | -0.35 | 0.31 |
|  |  | CtoN3 | -0.09 | -0.35 | 0.35 | 0.01 | -0.30 | 0.37 |
|  | NI-NC | NtoN1 | 2.16 | 1.52 | 2.73 | -0.25 | -1.18 | 0.68 |
|  |  | NtoN2 | 0.002 | -0.40 | 0.31 | 0.01 | -0.31 | 0.31 |
|  |  | NtoC1 | 0.03 | -0.36 | 0.33 | -0.05 | -0.31 | 0.30 |

*Note*: Modes and LB/UB HPD are calculated according to the 1000 iterations for the mixed-effect model (see main text Section 2.3). See Figure 1 and 7 for details about the hypotheses.
