# Supplementary material for: Spatial memory distortions for the shapes of walked paths occur in violation of physically experienced geometry
Source: PLoS One. 2023 Feb 10;18(2):e0281739. doi: 10.1371/journal.pone.0281739 (PMC9916584; doi:10.1371/journal.pone.0281739)
Supplement: S4 Table — Posterior mode and 95% highest posterior density (HPD) lower (LB) and upper bounds (UB) of the random effect variances in regression for each group and condition. (DOCX) [file pone.0281739.s016.docx]

S4 Table. *Posterior mode and 95% highest posterior density (HPD) lower (LB) and upper bounds (UB) of the random effect variances in regression for each group and condition.*

| **Experiment** | **Condition** | **Mode** | **LB HPD** | **UB HPD** |  |
| --- | --- | --- | --- | --- | --- |
| 1a (hallway mixed-design) | HI-C | 0.61 | 0.54 | 0.70 |  |
|  | NI-NC | 0.23 | 0.18 | 0.29 |  |
| 1b (pole-guided) | PG-C | 0.13 | 0.11 | 0.17 |  |
|  | PG-NC | 0.34 | 0.29 | 0.43 |  |
| 1c (hallway blocked-design) | HI-C | 0.58 | 0.52 | 0.65 |  |
|  | NI-NC | 0.29 | 0.25 | 0.37 |  |
| 2 | HI-C | 0.31 | 0.26 | 0.39 |  |
|  | TI-C | 0.30 | 0.27 | 0.38 |  |
|  | NI-NC | 0.05 | 0.04 | 0.06 |  |
|  | FI-NC | 0.03 | 0.02 | 0.04 |  |
| 3 | TI-C | 0.25 | 0.22 | 0.27 |  |
|  | FI-NC | 0.04 | 0.03 | 0.05 |  |

*Note*: Modes and LB/UB HPD are calculated according to the 1000 iterations for the mixed-effect model (see main text Section 2.3).
