# Supplementary material for: Spatial memory distortions for the shapes of walked paths occur in violation of physically experienced geometry
Source: PLoS One. 2023 Feb 10;18(2):e0281739. doi: 10.1371/journal.pone.0281739 (PMC9916584; doi:10.1371/journal.pone.0281739)
Supplement: S5 Table — (DOCX) [file pone.0281739.s017.docx]

S5 Table. *Path dimensions (in meters) in Experiments 2-4.*

| **Path** | **Path type** | **L1** | **L2** | **L3** | **L4** | **Total length** |
| --- | --- | --- | --- | --- | --- | --- |
| 1 | Cross | 3.99 | 1.70 | 2.12 | 2.38 | 10.2 |
| 2 | Cross | 3.49 | 1.93 | 1.95 | 2.82 | 10.2 |
| 3 | Cross | 3.12 | 1.98 | 2.20 | 2.90 | 10.2 |
| 4 | Cross | 2.89 | 1.87 | 1.97 | 3.47 | 10.2 |
| 5 | No cross | 2.12 | 1.70 | 3.99 | 2.38 | 10.2 |
| 6 | No cross | 1.93 | 1.96 | 3.47 | 2.84 | 10.2 |
| 7 | No cross | 1.73 | 2.45 | 2.65 | 3.37 | 10.2 |
| 8 | No cross | 1.98 | 1.86 | 2.90 | 3.45 | 10.2 |

*Note*: L1: From the start (S) to the first turning point (T1). L2: From T1 to the second turning point (T2). L3: From T2 to the third turning point (T3). L4: From T3 to the end (E). See Figure 7 for more details.
