# Supplementary material for: Spatial memory distortions for the shapes of walked paths occur in violation of physically experienced geometry
Source: PLoS One. 2023 Feb 10;18(2):e0281739. doi: 10.1371/journal.pone.0281739 (PMC9916584; doi:10.1371/journal.pone.0281739)
Supplement: S6 Table — Posterior modes and 95% highest posterior density (HPD) lower (LB) and upper bounds (UB) for the regression coefficients of the angular error for each group and condition. (DOCX) [file pone.0281739.s018.docx]

S6 Table. *Posterior modes and 95% highest posterior density (HPD) lower (LB) and upper bounds (UB) for the regression coefficients of the angular error for each group and condition in Experiment 2.*

| **Condition** | **Model** | **Component I** | | | **Component II** | | |
| --- | --- | --- | --- | --- | --- | --- | --- |
|  |  | **Mode** | **LB HPD** | **UB HPD** | **Mode** | **LB HPD** | **UB HPD** |
| HI-C | CtoC | 0.01 | -0.33 | 0.32 | -0.03 | -0.31 | 0.29 |
|  | CtoN1 | -1.83 | -2.22 | -1.15 | 0.10 | -0.57 | 0.94 |
|  | CtoN2 | -0.03 | -0.32 | 0.36 | -0.02 | -0.32 | 0.32 |
|  | CtoN3 | 0.07 | -0.34 | 0.30 | -0.03 | -0.28 | 0.33 |
| NI-NC | NtoN1 | 0.04 | -0.43 | 0.40 | 0.11 | -0.44 | 0.50 |
|  | NtoC2 | -3.32 | -4.09 | -2.56 | -4.19 | -5.03 | -3.39 |
|  | NtoC3 | -0.07 | -0.47 | 0.37 | -0.08 | -0.52 | 0.44 |
| TI-C | CtoC | 0.03 | -0.37 | 0.32 | -0.01 | -0.30 | 0.32 |
|  | CtoN1 | -2.18 | -3.15 | -1.70 | -0.25 | -1.53 | 0.70 |
|  | CtoN2 | 0.02 | -0.38 | 0.33 | -0.01 | -0.31 | 0.32 |
|  | CtoN3 | -0.07 | -0.33 | 0.31 | -0.05 | -0.32 | 0.29 |
| FI-NC | NtoN1 | 0.06 | -0.48 | 0.44 | 0.03 | -0.44 | 0.42 |
|  | NtoC2 | 3.08 | 2.66 | 3.63 | -2.86 | -3.45 | -2.42 |
|  | NtoC3 | 0.06 | -0.48 | 0.43 | -0.01 | -0.48 | 0.41 |

*Note*: Modes and LB/UB HPD are calculated according to the 1000 iterations for the mixed-effect model (see main text Section 2.3). See Figure 1 and 7 for details about the hypotheses.
