# Supplementary material for: Spatial memory distortions for the shapes of walked paths occur in violation of physically experienced geometry
Source: PLoS One. 2023 Feb 10;18(2):e0281739. doi: 10.1371/journal.pone.0281739 (PMC9916584; doi:10.1371/journal.pone.0281739)
Supplement: S7 Table — Posterior modes and 95% highest posterior density (HPD) lower (LB) and upper bounds (UB) for the regression coefficients of the angular error for each group and condition. (DOCX) [file pone.0281739.s019.docx]

S7 Table. *Posterior modes and 95% highest posterior density (HPD) lower (LB) and upper bounds (UB) for the regression coefficients of the angular error for each group and condition in Experiment 3.*

| **Condition** | **Model** | **Component I** | | | **Component II** | | |
| --- | --- | --- | --- | --- | --- | --- | --- |
|  |  | **Mode** | **LB HPD** | **UB HPD** | **Mode** | **LB HPD** | **UB HPD** |
| TI-C | CtoC | 0.02 | -0.34 | 0.28 | -0.06 | -0.25 | 0.30 |
|  | CtoN1 | -4.22 | -5.06 | -3.22 | 0.51 | -1.07 | 1.76 |
|  | CtoN2 | 0.02 | -0.34 | 0.32 | -0.01 | -0.25 | 0.31 |
|  | CtoN3 | 0.03 | -0.32 | 0.34 | 0.04 | -0.28 | 0.28 |
| FI-NC | NtoN1 | 0.06 | -0.38 | 0.41 | 0.05 | -0.36 | 0.39 |
|  | NtoC2 | 3.91 | 3.21 | 4.75 | -3.39 | -4.00 | -3.02 |
|  | NtoC3 | 0.03 | -0.49 | 0.38 | -0.02 | -0.40 | 0.41 |

*Note*: Modes and LB/UB HPD are calculated according to the 1000 iterations for the mixed-effect model (see main text Section 2.3). See Figure 1 and 7 for details about the hypotheses.
