# Supplementary material for: Spatial memory distortions for the shapes of walked paths occur in violation of physically experienced geometry
Source: PLoS One. 2023 Feb 10;18(2):e0281739. doi: 10.1371/journal.pone.0281739 (PMC9916584; doi:10.1371/journal.pone.0281739)
Supplement: S1 Text — (DOCX) [file pone.0281739.s020.docx]

**Supplementary Text**

**A. Outlier detection procedure for pointing angles**

We detected outliers for pointing angles after averaging across trials for each path and each participant. This procedure intends to exclude outliers using the observed c-statistics. The c-statistic can indicate the discordancy of any circular data point (Abuzaid, 2010; Collett, 1980). A larger value of c-statistic means that the data point is farther away from the mean and more likely to be an outlier. The distribution of c-statistics greatly depends on the variance of the sample which is indicated by the statistic *kappa*. A larger kappa value means smaller variance of the sample data; 1/kappa is related to angular standard deviation (Batschelet, 1981). If kappa = 0, the distribution is uniform; if values for kappa are large, the distribution approaches a normal distribution.

To find the cut-off value of the observed c-statistics in order to exclude potential outliers, we created a simulation program in R (see supplementary material “calc_cstats_cut_off.R”). This simulation program generated 1000 random samples with a mean of 0, the observed sample size, and the observed kappa, and returned a distribution of the maximum of c-statistics in each of the 1000 samples. Using a probability of 90%, we determined a cut-off value of c-statistics.

In the simulation, we always “rounded up” the observed kappa and the observed sample sizes. For example, in Experiment 1a the hallway mixed-design group cross condition (HI-C), the observed kappa before exclusion was 0.51. There were 128 data points for this group. Since the observed kappa was very likely to be underestimated with potential outliers, we used a kappa value of 0.7 that was larger than the observed kappa in the simulation. We used a sample size of 150 that was also larger than the observed sample size in the simulation. The observed kappa after excluding outliers was 0.74, which was improved from the original value and not far away from the used kappa 0.7.

The kappa value used in the simulation was determined by getting the difference between this value and the returned kappa value after exclusion. A kappa value would be acceptable if a) this difference was smaller than 1.5, b) the observed kappa after excluding outliers was improved from the original value, and c) the excluded data points were less than 10% of the total data.

In Experiment 1a, the hallway mixed-design group HI-C condition excluded 7.81% of the data as outliers. However, this is due to the larger variability of the original observed data in this group (S4 Fig). The cut-off value of c-statistic was 0.03182839 (we are using more than two decimals here just to show the value more explicitly). Therefore, we excluded the 10 data points whose c-statistics were larger than this value (red dots in the plot). It is very clear that the outliers largely influenced the distribution of the sample data (both mean and variance).

**B. The groups in which more than 5% outliers were excluded showed comparable results before and after excluding outliers**

For the groups in which more than 5% outliers were excluded, we also present the results without removing the outliers.

**a. Experiment 1a HI-C.** The average pointing angle was deviated from the start position 82.90° to the clockwise right of the actual starting location (Figures A, B in S5 Fig; 95% CI = [20.84°, 144.96°]). Comparisons with the hypothesis predictions indicates that the observed pointing direction was consistent with the prediction of the Cross to No cross (CtoN1) hypothesis (mean ratio = 0.46; Figures C, D in S5 Fig), which is consistent with the results after removing the outliers.

**b. Experiment 1b PG-NC.** The average pointing angle was deviated from the start position 320.05° to the counterclockwise of the actual starting location (Figures E, F in S5 Fig; 95% CI = [295.77°, 344.34°]). Comparisons with the hypothesis predictions indicates that the observed pointing direction was consistent with the prediction of the No cross to No cross (NtoN1) hypothesis (mean ratio = 0.68; Figures G, H in S5 Fig), which is consistent with the results after removing the outliers.

**c. Experiment 1c NI-NC.** The average pointing angle was deviated from the start position 328.32° to the counterclockwise of the actual starting location (Figures I, J, in S5 Fig; 95% CI = [309.53°, 347.12°]). Comparisons with the hypothesis predictions indicates that the observed pointing direction was consistent with the prediction of the No cross to No cross (NtoN1) hypothesis (mean ratio = 0.64; Figures K, L in S5 Fig), which is consistent with the results after removing the outliers.

**d. Experiment 2 HI-C.** The average pointing angle was deviated from the start position 40.16° to the clockwise of the actual starting location (Figures M, N in S5 Fig; 95% CI = [18.23°, 62.09°]). Comparisons with the hypothesis predictions indicates that the observed pointing direction was consistent with the predictions of the Cross to Cross (CtoC) and Cross to No cross (CtoN1) hypothesis (mean ratio = 0.62, 0.43, respectively; Figures O, P in S5 Fig), which is consistent with the results after removing the outliers.

**C. Participants showed a higher absolute angular error (AE_G_) in the HI-C and FI-NC conditions**

Since the absolute AE_G_ were linear data and did not follow the same circular distribution as AE_G_, we did not exclude any outliers.

**a. Experiment 1.** We first examined the two mixed-designed groups (Experiment 1a vs 1b). Using the absolute AE_G_ as dependent measure (S6 Fig), we conducted a 2 (Group: Hallway, Pole-guided) × 2 (Path type: No cross, Cross) mixed ANOVA. We found that there was a significant interaction effect, *F*(1, 51) = 15.96, *p* < .001, *η_p_*^2^ = .24. Neither of the main effects were significant (both *p*s > .12, *η_p_*^2^ < .05). Further analysis showed that for the cross condition, the hallway group (mean = 85.57°, SD = 39.13) showed a larger error than the pole-guided group (mean = 53.80°, SD = 22.40), *t*(51) = 3.37, *p* = .001, Cohen’s *d* = 0.95, whereas for the no cross condition, the two groups did not show any significant difference (hallway: mean = 55.89°, SD = 28.95; pole-guided: mean = 66.97°, SD = 31.65), *t*(51) = 1.31, *p* = .195, Cohen’s *d* = 0.37.

For the hallway blocked-designed group (Experiment 1c), using the absolute AE_G_ as dependent measure, a paired *t* test was conducted. There was a significant difference between the conditions, *t*(30) = 2.83, *p* = .008, Cohen’s *d* = 0.72. Participants showed a significantly higher error in the HI-C condition (mean = 75.70°, SD = 43.75) than in the NI-NC condition (mean = 55.17°, SD = 36.26), which is consistent with the result in the hallway mixed-designed group, *t*(31) = 3.82, *p* < .001, Cohen’s *d* = 0.67.

**b. Experiment 2.** Using the absolute AE_G_ as dependent measure, we conducted a 2 (Intersection visibility: Hidden, Shown) × 2 (Path type: No cross, Cross) within-subject ANOVA. We found that there was a significant main effect of Intersection visibility, *F*(1, 31) = 293.27, *p* < .001, *η_p_*^2^ = .90. Neither of the main effect of Path type nor the interaction was significant (both *p*s > .05, *η_p_*^2^ < .11). Planned comparisons showed that for the no cross condition, participants showed larger errors when the intersections were shown (FI-NC; mean = 86.00°, SD = 14.88) than when they were hidden (NI-NC; mean = 24.63°, SD = 7.04), *t*(31) = 30.55, *p* < .001, Cohen’s *d* = 7.64, whereas for the cross condition, there was no significant difference (TI-C: mean = 63.94°, SD = 35.18; HI-C: mean = 65.58°, SD = 33.31), *t*(31) = 0.70, *p* = .489, Cohen’s *d* = 0.18.

**c. Experiment 3.** Using the absolute AE_G_ as dependent measure, we conducted a paired-sample *t* test and found that participants showed a significantly higher error in the FI-NC condition (mean = 84.89°, SD = 14.92) than in the TI-C condition (mean = 52.68°, SD = 33.28), *t*(42) = 7.36, *p* < .001, Cohen’s *d* = 1.59, which is consistent with the result in Experiment 2.

**d. Experiment 4.** Using the absolute AE_G_ as dependent measure, we conducted a 2 (Intersection visibility: Hidden, Shown) × 2 (Path type: No cross, Cross) within-subject ANOVA. We found that there was a significant main effect of Path type, *F*(1, 24) = 7.23, *p* = .013, *η_p_*^2^ = .23. The crossed path trials (mean = 85.68°, SD = 42.77) showed larger errors than uncrossed path trials (mean = 58.32°, SD = 39.88). Neither of the main effect of Intersection visibility nor the interaction was significant (both *p*s > .37, *η_p_*^2^ < .04). There was no significant difference between the HI-C and TI-C conditions (*t*(24) = 0.46, *p* = .649, Cohen’s *d* = 0.09) or between NI-NC and FI-NC conditions (*t*(24) = 1.45, *p* = .161, Cohen’s *d* = 0.29).

Overall, across all experiments, participants showed higher absolute pointing errors in conditions in which conflicting information were presented (HI-C and FI-NC).

**D. Experiments 2-4: Participants were less confident in their response in the cross conditions**

If the confidence rating (CR) on one trial equaled 0.5 (the initial value), this trial was excluded from analysis. In Experiment 2, one participant was excluded from this analysis because 15 trials of their CR responses were excluded. Other than this participant, there were 14 trials excluded in total.

Using CR as dependent measure (S8 Fig), we conducted a 2 (Intersection visibility: Hidden/No, Shown) × 2 (Path type: No cross, Cross) within-subject ANOVA. We found that there was a significant main effect of Path type, *F*(1, 30) = 5.78, *p* = .023, *η_p_*^2^ = .16. The CR in the cross condition (mean = 0.62, SD = 0.15) was lower than that in the no cross condition (mean = 0.64, SD = 0.14). Neither of the main effect of Intersection visibility nor the interaction was significant (both *p*s > .07, *η_p_*^2^ < .11). Further analysis showed that there was no significant difference between the two cross conditions (HI-C vs. TI-C), *t*(31) = 0.84, *p* = .410, Cohen’s *d* = 0.21. Participants were less confident about their pointing responses in the cross conditions than in the no-cross conditions.

In Experiment 3, there were 38 trials excluded due to CR = 0.5 and no participants were excluded. A paired-sample *t* test showed that there was no significant difference between the two conditions, *t*(42) = 1.08, *p* = .287, Cohen’s *d* = 0.23. The confidence rating in the TI-C condition (mean = 0.61, SD = 0.14) was at the same level as in the FI-NC condition (mean = 0.61, SD = 0.13).

In Experiment 4, there were 129 trials (5.38%) excluded due to CR = 0.5. Using CR as dependent measure (S8 Fig), we conducted a 2 (Intersection visibility: Hidden/No, Shown) × 2 (Path type: No cross, Cross) within-subject ANOVA. Neither of the main effects nor the interaction was significant (all *p*s > .09, *η_p_*^2^ < .12). The confidence ratings in all conditions were at the same level (HI-C: mean = 0.68, SD = 0.19; NI-NC: mean = 0.67, SD = 0.21; TI-C: mean = 0.65, SD = 0.20; FI-NC: mean = 0.66, SD = 0.21).
