# Supplementary material for: Correction: Spatial memory distortions for the shapes of walked paths occur in violation of physically experienced geometry
Source: PLoS One. 2026 Oct 26;21(10):e0361361. doi: 10.1371/journal.pone.0361361 (PMC13704848; doi:10.1371/journal.pone.0361361)
Supplement: Supplementary file 2 [file pone.0361361.s005.docx]

S7 Table. *Posterior modes and 95% highest posterior density (HPD) lower (LB) and upper bounds (UB) for the regression coefficients of the angular error for each group and condition in Experiment 3.*

| **Condition** | **Model** | **Component I** | | | **Component II** | | |
| --- | --- | --- | --- | --- | --- | --- | --- |
|  |  | **Mode** | **LB HPD** | **UB HPD** | **Mode** | **LB HPD** | **UB HPD** |
| TI-C | CtoC | 0.06 | -0.40 | 0.40 | 0.05 | -0.34 | 0.34 |
|  | CtoN1 | -3.76 | -5.07 | -2.84 | 0.2 | -1.86 | 2.00 |
|  | CtoN2 | -0.05 | -0.40 | 0.38 | 0.01 | -0.36 | 0.32 |
|  | CtoN3 | -0.0007 | -0.37 | 0.42 | 0.06 | -0.33 | 0.32 |
| FI-NC | NtoN1 | 0.04 | -0.37 | 0.32 | -0.07 | -0.53 | 0.45 |
|  | NtoC2 | -3.26 | -3.87 | -2.58 | -5.94 | -6.86 | -4.83 |
|  | NtoC3 | -0.06 | -0.33 | 0.34 | -0.03 | -0.49 | 0.44 |

*Note*: Modes and LB/UB HPD are calculated according to the 1000 iterations for the mixed-effect model (see main text Section 2.3). See Figure 1 and 7 for details about the hypotheses.
